# Phenological and Geographical Effects on Phenolic and Triterpenoid Content in *Vaccinium vitis-idaea* L. Leaves

**DOI:** 10.3390/plants10101986

**Published:** 2021-09-23

**Authors:** Gabriele Vilkickyte, Lina Raudone

**Affiliations:** 1 Laboratory of Biopharmaceutical Research, Institute of Pharmaceutical Technologies, Lithuanian University of Health Sciences, Sukileliu av. 13, LT-50162 Kaunas, Lithuania; lina.raudone@lsmuni.lt; 2 Department of Pharmacognosy, Lithuanian University of Health Sciences, Sukileliu av. 13, LT-50162 Kaunas, Lithuania

**Keywords:** *Vaccinium vitis-idaea* L., lingonberry leaves, HPLC-PDA, seasonal and geographical variation, phenolics, triterpenoids

## Abstract

Lingonberry leaves have been proposed as a potential raw material for nutraceutical products and functional food due to the richness of phenolic and triterpenic compounds. However, contents of these bioactive compounds tend to vary greatly with physiological, climatic, and edaphic conditions, resulting in lingonberry leaves’ nutritional-pharmaceutical quality changes. In this context, we examined the effects of seasonal and geographical factors on phenolic and triterpenoid contents in lingonberry leaves. Quantitative and qualitative differences between samples were determined using validated HPLC-PDA methods. A total of 43 bioactive compounds were found at a detectable level throughout the year in young and old lingonberry leaves, with the highest contents of most compounds observed in samples collected in autumn–first half of spring. This suggests the potential to exploit the continuous biosynthesis for a longer harvesting season. Considerable variations in phytochemical profiles of lingonberry leaves, obtained from 28 locations in Lithuania, were found. Correlation analyses revealed significant negative correlations between contents of particular constituents and sunshine duration, temperature, and precipitation, and positive correlation with air humidity, longitudes, and altitudes of collecting locations and macronutrients in soil. These results suggest that harsh weather is favorable for most identified compounds and it may be possible to achieve appropriate accumulation of secondary metabolites by adjusting edaphic conditions. Taken together, the accumulation of phenolics and triterpenoids in lingonberry leaves highly depends on phenological and geographical factors and the influence of both variables differ for the particular compounds due to different metabolic processes in response to stresses.

## 1. Introduction

Lingonberry (*Vaccinium vitis-idaea* L.; family *Ericaceae*), otherwise known as cowberry, partridgeberry, and foxberry, is an evergreen dwarf shrub with underground rhizomes widely distributed throughout Northern, Central European, Canadian, and Russian countries’ forests. Lingonberry is extremely hardy, tolerating cold climate, drought, and diverse habitats—from exposed, dry slopes and peat soils to ombrotrophic bogs [1,2,3,4]. Fruits of lingonberry are gaining notoriety as “superfoods”, because of health benefits deriving from their consumption and their significant economic importance [5]. However, due to the limited seasonal availability of fresh fruits, leaves have been proposed as a potential alternative raw material for functional foods [6]. It was pointed out that *Vaccinium* leaves possess even higher biological activity than fruits, making lingonberry leaves an interesting resource of pharmaceutical ingredients [4,7]. An increasing number of studies have shown that extracts of lingonberry leaves have astringent, antitussive, urinary tract antiseptic, diuretic, neuroprotective, antioxidant, and anti-inflammatory effects, and may inhibit cancer cell growth [3,6,8]. Due to the promising health benefits, mainly associated with the high content of secondary metabolites—phenolics and triterpenoids—lingonberry leaves acquired prominent pharmaceutical potential and breeding value [1,9]. The phenolic compounds in lingonberry leaves are composed of arbutin derivatives, proanthocyanidins, catechins, flavonols, and phenolic acids, whereas triterpenic profile includes triterpenoid acids, neutral triterpenoids, and sterols. They can occur at different levels and vary over time and space considerably due to chemophenetic differences [10,11,12,13].

Many variables have been proven to have an impact on plant physiological processes and the yield of secondary metabolites. Abiotic factors include exposure to the light, temperature, soil properties, macronutrients, moisture, water availability, and the altitude of the growing site, whereas biotic factors includeplant coverage, species richness, and evenness, as well as interactions with neighboring plants, herbivores, and pathogens [14,15,16]. Since different abiotic and biotic conditions have been considered as possible reasons for the chemodiversity among the same species growing at different geoclimates, recent years have witnessed renewed attention to the influence of these factors on secondary plant metabolism [14,15]. Other sources of secondary metabolite variance observed among plants are different physiological and developmental stages and growing season length, which is closely related to environmental factors [16]. The understanding of plant physiological and metabolic processes during the phenological cycle are key aspects, serving for the optimization of plant quality traits [17]. Although it is well known that various factors can affect the concentration of bioactive compounds in lingonberry leaves, knowledge of climatic and edaphic conditions that influence phenolic and triterpenoid accumulation is still lacking. The possible compositional changes in bioactive compounds during vegetation would have scientific value in terms of the metabolic pathways of these compounds.

In the light of these concerns, the present study aimed to investigate the effects of growing location and phenological growth stage on phenolic and triterpenoid content in lingonberry leaves. To date, very little information is available on the accumulation of phenolics and triterpenoids in wild-growing lingonberry leaves in correlation to the geographical habitat, environmental conditions, and soil quality parameters. In addition, to our knowledge, this is the first comprehensive study investigating metabolic processes of bioactive compounds in lingonberry leaves throughout the year.

## 2. Results and Discussion

### 2.1. Seasonal Variation of Phenolic and Triterpenic Compounds in Lingonberry Leaves

#### 2.1.1. Phenological Profiling by HPLC-PDA

A total of 43 compounds, involving major groups of secondary metabolites—phenolic and triterpenic compounds—were found in extracts of lingonberry leaves. Being an evergreen species, lingonberry keeps its leaves thick and waxy even during the coldest months [4,15]. Our results showed that most compounds were detected at a quantifiable level throughout the year thus proving continuous biosynthesis and storage of secondary metabolites in lingonberry leaves. Contents of all compounds varied along the year and different groups of phenolic and triterpenic compounds showed different patterns of seasonal variation (accumulation patterns of particular constituents in young and old lingonberry leaves, collected throughout one year, are presented in Supplementary Tables S1–S7). The content of secondary metabolites varies due to modifications in gene expression or their encoded protein activity involved in secondary metabolism in the presence of different developmental stages, stresses, and adaptation to them [18]. The production of different compounds is not evenly affected by these factors, depending on the main biosynthesis pathway. The current understanding of metabolic pathways of all compounds has not been comprehensively defined yet and is still the topic of genetic studies. However, it has now been proposed that phenolic compounds synthesis is a combinatorial biosynthesis, including shikimate and acetate pathways, whereas triterpenic compounds are basically formed by the mevalonate pathway with some alternative routes and many different enzymes involved [19,20,21]. Since phenological and physiological factors influence the biosynthesis of plant secondary metabolites, seasonality and harvest time stand as critical factors determining the chemical composition [22].

**Figure 1:**
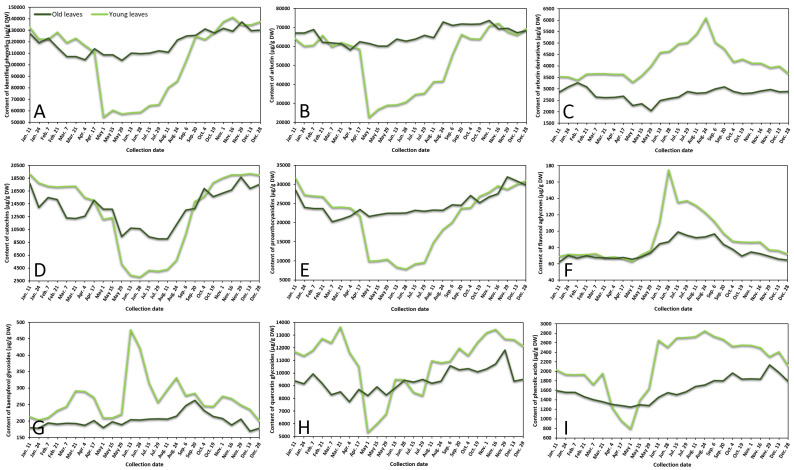
Variation in sums of (**A**) all identified phenolics, (**B**) arbutin, (**C**) arbutin derivatives, (**D**) catechins, (**E**) proanthocyanidins, (**F**) flavonol aglycones, (**G**) kaempferol glycosides, (**H**) quercetin glycosides, and (**I**) phenolic acids (μg/g DW) in young (light line) and old (dark line) lingonberry leaves during one year testing period. Abbreviations of months have been defined in Appendix A.

The variance of sum contents of different subgroups of phenolics in young and old lingonberry leaves, collected during one year period, is shown in Figure 1. The most important contributor to the leaves extracts was arbutin, accounting for 42–60% of total identified phenolics at different collection dates. Strong correlation between arbutin level and total identified phenolics (*r* = 0.987, *p* < 0.05) resulted in similar fluctuation curves (Figure 1A,B). The content of arbutin in old lingonberry leaves was quite stable with the lowest coefficient of variation (CV)—7%, compared to other identified phenolic compounds. Nevertheless, the highest (*p* < 0.05) levels were detected at the end of the summer and autumn. Levels of arbutin in young current-year lingonberry leaves were similar to those found in old previous-year leaves, except for a notable decrease in amount during the May–August months. These basic findings are partly consistent with a previous study showing that arbutin level is highest in autumn, suddenly decreasing during the burst of buds and slowly increasing during flowering [23].

Similar accumulation and content decreasing patterns were obtained for other important contributors—catechins (the sum of contents of (+)-catechin and (–)-epicatechin) and proanthocyanidins (procyanidins A1, A2, A4, B1, B2, B3, C1), which made up to 15% and 24% of total identified phenolics, respectively (Figure 1D,E). These groups were characterized by high CV, with the greatest variation (50%) of catechins in current-year lingonberry leaves. The highest (*p* < 0.05) levels of most proanthocyanidins and catechins were observed in 16 November–11 January samples, while the content of these compounds was about 1.5-fold and 3-fold lower in old and young lingonberry leaves taken on 1 May–24 August, respectively. These results go beyond the previous report about increasing biosynthesis of flavanol monomers and oligomers in lingonberry leaves from May to September, and which found the greatest amounts in autumn [24].

In contrast to these accumulation patterns, arbutin derivatives (hydroquinone and 2-*O*-caffeoylarbutin), minor contributors—flavonol aglycones (kaempferol, quercetin) and kaempferol glycosides (nicotiflorin, astragalin, afzelin)—were present in an intermittently increasing trend from 1 May till 24 August in samples of young leaves, whereas in previous-year lingonberry leaves they appeared in more constant mode with the highest (*p* < 0.05) concentrations on 7 February (3266.0 μg/g dry weight (DW)), 15 July (99.1 μg/g DW), and 20 September (262.7 μg/g DW) in case of arbutin derivatives, flavonol aglycones, and kaempferol glycosides, accordingly (Figure 1C,F,G). Similarly, Bujor et al. [24] reported that arbutin derivative—2-*O*-caffeoylarbutin in lingonberry leaves exhibited a seasonal increase, that started in May.

The metabolic pattern of quercetin glycosides (quercitrin, quercetin-HMG-rhamnoside, 6′′-*O*-acetylisoquercitrin, rutin, hyperoside, isoquercitrin, reynoutrin, guaiaverin, and avicularin) and phenolic acids (chlorogenic, cryptochlorogenic, neochlorogenic, and *p*-coumaric acids) was markedly distinguished by the lowest (*p* < 0.05) amounts in the 1 May samples of current-year leaves and sharply increased up to 13,443.2 μg/g DW on 16 November in case of quercetin glycosides and up to 2851.3 μg/g DW on 24 August in case of phenolic acids (Figure 1H,I). In old lingonberry leaves, levels of quercetin glycosides (CV = 10%) ranged between 7725.2 μg/g DW on 4 April and 11,823.3 μg/g DW on 29 November, and the content of phenolic acids (CV = 15%) was in a range of 1245.8–2135.4 μg/g DW (1 May–29 November). The same increasing tendency of flavonol glycosides was obtained previously when assessing contents of flavonol glycosides in leaves of another *Vaccinium* member—bilberry— in May, July, and September [25].

Overall, in many cases levels of phenolics in old lingonberry leaves fluctuated but showed no discernible trend, whereas phenolics in current-year leaves showed sharply increases or decreases, outlining a major annual effect during the growth season. The observed changes in phenolic compounds during spring–summer seasons, preeminent in the beginning of May could be attributed to the production of new current-year leaves and intensified biosynthesis of secondary metabolites. A similar annual effect was reached by Sommavilla et al. [26], which analyzed seasonal variation of phenolics in leaves of *Celtis australis* L. It seems likely that the initial level of phenolics may be even higher than in previous-year leaves or be significantly lower with a slow increasing tendency, highly depending on the group of phenolic compounds. Seasonal variance is expected to be more stable in previous-years leaves due to slower metabolism processes, decay of some compounds, and also because young leaves, which are on the upper position of the stem, are more likely to be affected by UV-screening, acclimatization to light conditions, and other biotic and abiotic elicitors [27]. A recent study calculated about 1.5–8-fold lower CVs of most phenolics in old leaves, compared to young leaves. CV in young leaves varied between 14 and 111%, whereas in old leaves it ranged between 7 and 104% with the highest variation of hydroquinone in both cases, followed by *p*-coumaric acid, kaempferol, nicotiflorin, procyanidin A4, rutin, and (+)-catechin. It can thus be suggested that the metabolism of these compounds is strongly affected by environmental and phenological factors. It was reported in the literature that with the growth of evergreen plants, the content and yield of secondary metabolites tend to be higher and higher [18]. Our results showed that average annual amounts of particular compounds in old leaves were slightly higher (1.1–1.2-fold) than those in young leaves in cases of arbutin, catechins, and proanthocyanidins, and lower (1.2–1.5-fold) when considering amounts of arbutin derivatives, flavonols, and phenolic acids. The reason for these partly contradictory results is still not entirely clear, but it can be supported by variable decay processes of compounds and different adaptations to weather conditions.

**Figure 2:**
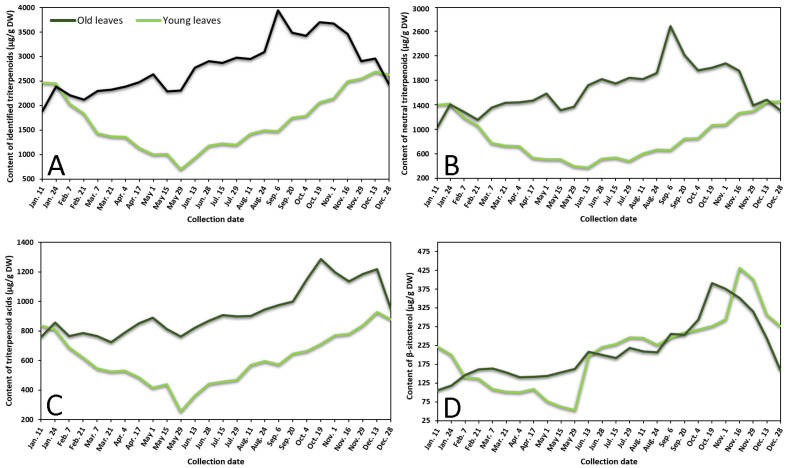
Variation in sums of (**A**) all identified triterpenoids, (**B**) neutral triterpenoids, (**C**) triterpenoid acids, and (**D**) β-sitosterol (μg/g DW) in young (light line) and old (dark line) lingonberry leaves during one year testing period.

Contents of neutral triterpenoids, triterpenoid acids, sterols, and total identified triterpenoids in lingonberry leaves were also variable according to collection time (Figure 2 represents the variance of sum contents of different subgroups triterpenoids in young and old lingonberry leaves, collected during a one-year period). The accumulation pattern of total identified triterpenoids was mainly influenced by contents of neutral triterpenoids (the sum of contents of betulin, erythrodiol, uvaol, lupeol, α-amyrin, β-amyrin, and friedelin), which made up 40–69% of total identified triterpenoids at different collection dates (*r* = 0.972, *p* < 0.05) and can be considered as a prevailing group in lingonberry leaves with the principal component—α-amyrin (Figure 2A,B). The highest (*p* < 0.05) contents of most neutral triterpenoids in young leaves emerged in colder months (16 November–7 February) with the greatest total content of 1472.0 μg/g DW on 28 December, whereas the accumulation peak in old leaves was reached earlier—on 6 September (total content of 2714.7 μg/g DW). The lowest (*p* < 0.05) levels of neutral triterpenoids from 29 May–13 June and 28 December–11 January were observed in young and old leaves, respectively. These findings correlate favorably with Szakiel et al. [12] and further support the concept that the content of neutral triterpenoids is considerably higher in old leaves during warm months, while in winter, content in young leaves reaches or even surpasses neutral triterpenoids level in old leaves.

Very similar fluctuation patterns of triterpenoid acids (maslinic, corosolic, betulinic, oleanolic, and ursolic acids) harvested throughout one year were found in young and old lingonberry leaves, but contents were more stable—approx. 1.5 and 1.3-fold lower values of CV, respectively, compared to neutral triterpenoids, were calculated (Figure 2C). The maximum sum values of triterpenoid acids with predominant ursolic acid out of this group occurred on 13 December (927.0 μg/g DW) and the lowest one was registered on 29 May (255.1 μg/g DW) in the case of current-year lingonberry leaves. Meanwhile, concentrations in old lingonberry leaves ranged between 724.8 and 1285.5 μg/g DW in samples taken on 21 March and 19 October, respectively. Rahajanirina et al. [28] indicated that the biosynthesis of triterpenoid acids, as well as other triterpenoids, may be enhanced during the cold and rainy months, while summer may be less favorable for biosynthetic processes.

Further data would be needed to determine exactly how the phenological stage affects the accumulation of all sterols, found in lingonberry leaves, but our preliminary study of variance of β-sitosterol, which has been reported to be the main compound out of this group [29] suggest that biosynthesis of sterols intensifies in late autumn. The highest (*p* < 0.05) concentration of sterols (β-sitosterol) was observed from 19 October–1 November (up to 390.7 μg/g DW) and 16–29 November (up to 431.2 μg/g DW) in samples of old and young lingonberry leaves, accordingly (Figure 2D). Different periods of year were outlined because of low contents; about a 4-fold lower amount of β-sitosterol, compared to its highest value, was found in samples harvested on 11 January in case of old lingonberry leaves and 8-fold lower on 29 May in the case of current-year leaves.

Our findings on seasonal variance clearly propose that biosynthesis of most triterpenoids appears to be most intensive in current-year leaves during late autumn–winter months, before production of new vegetative buds, whereas old leaves display an increasing tendency until the winter. The fact that old leaves are located in the bottom of the stem—usually beneath the snowpack during winter should be taken into consideration [27]. Some researchers claim that triterpenoid accumulation in leaves during snow covering acquires an important protective role against herbivores and fungal pathogens [30]. It is also noteworthy that the average annual amounts of all tested groups of triterpenoids in previous-year leaves were higher (1.2–1.8-fold) than those found in current-year leaves, reaching a similar level only in December–January. In contrast to phenolics, biosynthesis of triterpenoids seems to have an increasing trend with the increase of growth years, with likely slower decay processes and sustained metabolic activity. In addition, a greater variance of triterpenoids was found. Mean values of CV were twice and nearly 1.5-fold higher in old and young lingonberry leaves, accordingly. The highest variance due to phenological stages of the following individual triterpenoids was established: lupeol (CV = 70%), erythrodiol (68%), and maslinic acid (65%), while the most constant values were observed for ursolic (31%) and oleanolic acids (36%), suggesting different accumulation and adaptation processes to the growing season. Considering similarities to phenolics, the same annual increasing pattern of triterpenoids could be seen from May.

#### 2.1.2. Hierarchical Cluster Analysis of Phenolic and Triterpenic Compounds

**Figure 3:**
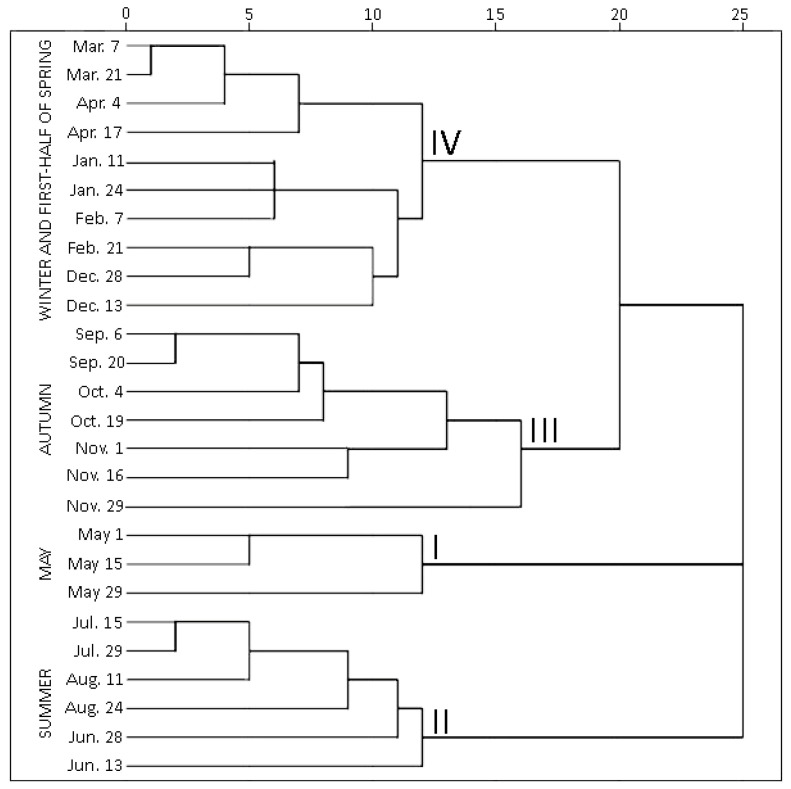
Dendrogram estimating contents of phenolic and triterpenic compounds in lingonberry leaves based on their collection date.

Hierarchical cluster analysis (HCA) was performed to highlight variations by grouping individual contents of phenolic and triterpenic compounds in samples of young and old lingonberry leaves in accordance with collection dates. Statistically significant differences were determined among four clusters (Figure 3). The first cluster was composed exclusively of samples collected in May—the month which is associated with swelling and bursting of vegetative and flower buds [23]. The samples forming this cluster were characterized by the lowest (*p* < 0.05) contents of all tested secondary metabolites, only kaempferol glycosides, neutral triterpenoids in previous-year leaves, and catechins in both-year leaves were considered as second-lowest content groups. This may be explained by the initial low content of secondary metabolites in new growing leaves in May, whereas the lowest levels in old leaves can be related to intensified secondary metabolites allocation and transfer via organs [31]. The second cluster grouped all samples, taken in summer (15 July–24 August)—reproductive growth period according to Bandzaitiene et al. [23]. These samples were distinguished by the lowest concentrations of catechins and the highest contents of flavonol aglycones in young and old lingonberry leaves, also by the maximum sum values of arbutin derivatives, kaempferol glycosides, and phenolic acids in current-year leaves. This indicates rapid accumulation of these compounds right away of leaves production. Cluster three included lingonberry leaves harvested in autumn (6 September–29 November), which is associated with the end of the lingonberry reproductive growth period [23]. These samples can be characterized by the highest levels of most tested compounds—proanthocyanidins, phenolic acids, kaempferol glycosides, arbutin derivatives, and catechins in previous-year leaves, also arbutin, quercetin glycosides, triterpenoid acids, and sterols in both-year leaves, thus pointing out optimum lingonberry leaf harvesting time. To the fourth cluster, samples taken on winter and first-half of spring (13 December–17 April) were attributed and distinguished by the highest levels of catechins, proanthocyanidins, and neutral triterpenoids in young leaves, and the lowest amounts of flavonol aglycones, kaempferol glycosides, and neutral triterpenoids in previous-year leaves. Different levels of particular phenolics during the cold snowy season suggest unequal acclimation to harsh weather conditions. The average amounts of identified phenolics and triterpenoids were approx. 1.5-fold higher in samples collected during the autumn–first half of spring, compared to the second half of spring–summer months. In general, samples of 3rd and 4th clusters can be outlined by their rich content of secondary metabolites, whereas samples of 1st and 2nd clusters by considerably lower amounts. This phenomenon is most notable when assessing variation of total identified constituents content (sums of identified phenolics and triterpenoids, which highly correlated with each other; *r* = 0.746, *p* < 0.05), regardless of the age of leaves (Supplementary Figure S1).

#### 2.1.3. Environmental Factors Analysis

**Figure 4:**
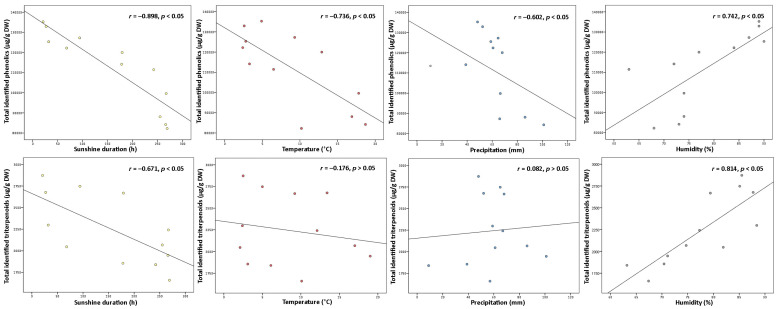
Correlations between sunshine duration (h), temperature (°C), precipitation (mm), and humidity (%) throughout the year with total identified phenolic and triterpenoid contents (µg/g DW) in lingonberry leaves.

Variation in phenolic and triterpenoids content may be affected not only by phenological stage and age of leaves but additionally by seasonal factors—temperature, sunshine duration, precipitation, humidity, and plant defense mechanisms to climatic conditions [32,33]. In response to weather fluctuations or negative abiotic factors, plants are able to adapt to the changes and trigger variation in secondary metabolites by their displacement between tissues and intensified accumulation [34]. Since environmental factors are crucial determinants for the changes in plant secondary metabolites, the contents of total identified (Figure 4) and individual compounds were examined for any correlations to known climatic conditions.

Among all abiotic factors, light plays an irreplaceable role in regulating plant phenology due to its direct relation to photosynthetic activity [35]. Light provides the energy, which is required for photosynthesis, promoting plant growth, and inducing or regulating plant metabolism through photoreceptors [32]. Most commonly moderate light intensity has a stimulatory effect on the formation of secondary metabolites, however, the effect can be different, depending on the plant, organ, and group of compounds [18]. The data analyses showed that levels of total phenolics and triterpenoids were negatively correlated with the temperature throughout the year (*r* = −0.898 and −0.671, respectively, *p* < 0.05). The strongest negative correlation to sunshine duration was found with proanthocyanidins and catechins from the phenolics group and with triterpenic acids from the triterpenoids group (*r* = −0.926 and −0.883, −0.790 respectively, *p* < 0.05). It is still controversial why plants growing under shading conditions may experience an increase of secondary metabolites. Excessive levels of solar radiation may reduce carbon metabolism and cause photoinhibition by impairing the photosynthetic reaction centers of the chloroplasts [35]. Among all identified compounds, only kaempferol glycosides and flavonol aglycones correlated positively with sunshine duration (*r* = 0.683 and 0.611, respectively, *p* < 0.05), suggesting greater demand for light energy for their biosynthesis, compared to other constituents. In line with our study, a shorter sunshine duration caused a double reduction in the content of flavonol aglycones in aerial parts of *Xantium* spp. earlier [36]. Correlation results for sunshine duration indicated that it may be possible to adjust the desirable accumulation of secondary metabolites by adjusting light and dark regimes.

Plants’ growth and development are also directly linked with air temperature, which regulates the responding genes. A temperature range of 17–25 °C is generally considered optimal for the maintenance of plant cells and productivity; however, each plant species may exhibit optimum growth and metabolism under different temperatures [18,32]. Some plants, like lingonberry, can survive low-temperature stress, tolerate –40 °C or even lower temperatures, by inducing different physiological, biochemical, and molecular changes and thus increasing levels of particular compounds [4,14,21]. Cold acclimation phenomenon in our study can be most clearly evidenced by the negative correlation of total identified phenolics with air temperature (*r* = −0.736, *p* < 0.05) and the strongest correlations with catechins, proanthocyanidins, and arbutin out of the phenolic group (*r* > −0.700, *p* < 0.05). In line with our results, an adverse secondary metabolism response to higher temperature was reported to be expected in grapes, which are widely known as a rich source of proanthocyanidins and catechins [37]. Concerning the content of total triterpenoids, no significant correlation was found to temperature, only betulin as an individual triterpenoid was distinguished by a strong positive correlation (*r* = 0.781, *p* < 0.05).

Drought stress is known as a further key environmental factor profoundly affecting plant metabolism by regulating carbon allocation from the roots and nutrient circulation [38]. Severe water deficit has been considered to reduce plant development and decrease biomass production, but as reported recently, plants may indeed accumulate higher contents of secondary metabolites as a response to drought [14,39]. A moderate negative correlation was found between total phenolics content and precipitation level throughout the year (*r* = −0.602, *p* < 0.05) with the strongest correlation with catechins out of this group (*r* = −0.766, *p* < 0.05). It was reported that deficient water uptake induced high increases in contents of (+)-catechin and (–)-epicatechin in *Crataegus* spp. leaves [40], thus supporting the idea that drought may promote the production of tannins in plants. Some positive significant correlations between arbutin derivatives, kaempferol glycosides, phenolic acids, and flavonol aglycones to precipitation level (*r* up to 0.866, *p* < 0.05) were have also been found, suggesting that high water uptake is favorable for circulation and metabolism of these compound. Meanwhile, rainfall level was not closely linked to the contents of triterpenoids, and this statement is consistent with what has been found in a previous study by Alqahtani et al. [22].

Even though air humidity has more impact on the composition of volatile compounds, it also can shift the metabolism of other secondary metabolites even in perennial plants with wax layer leaves, as in lingonberries [38]. High humidity can reduce growth rate, the biomass of leaves, and bud size, and result in an earlier burst of buds, but simultaneously may lower hydrophobicity of leaf surface, exposing the leaves for fungal pathogen attacks [41]. Hence, positive changes in contents of phenolics and triterpenoids under higher air humidity can be explained as a defense mechanism. This concept ties well with our study, wherein strong positive correlations were found between total phenolic or triterpenoid contents and air humidity (*r* = 0.742, and 0.814, respectively, *p* < 0.05) with strongest correlations to contents of proanthocyanidins, arbutin, and triterpenoid acids (*r* > 0.700, *p* < 0.05).

### 2.2. Geographical Variation of Phenolic and Triterpenic Compounds in Lingonberry Leaves

#### 2.2.1. Phytogeographical Profiling by HPLC-PDA

**Figure 5:**
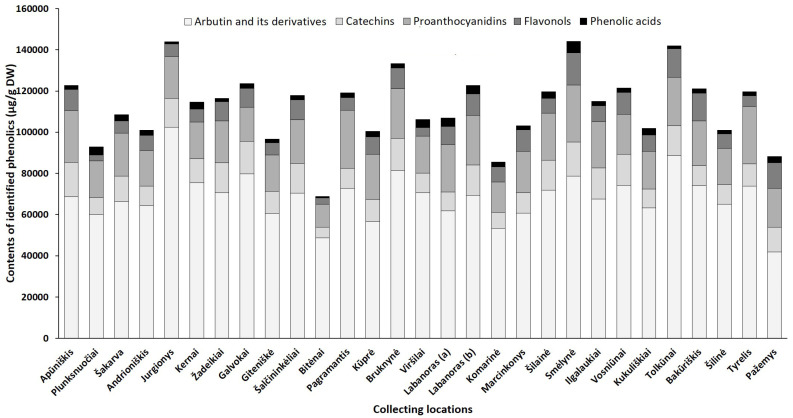
Sum contents of phenolics (μg/g DW) in lingonberry leaves, collected in different habitats.

**Figure 6:**
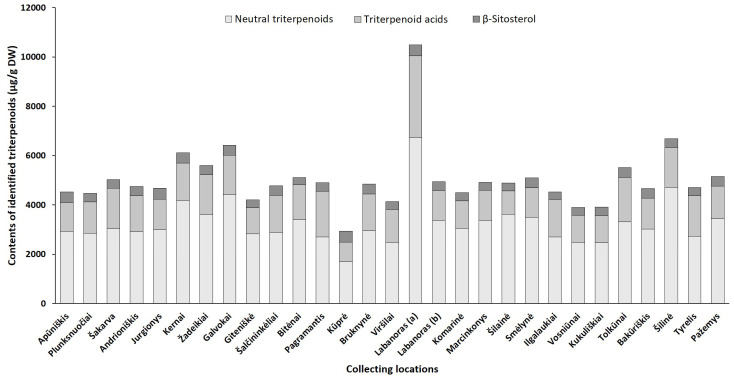
Sum contents of triterpenoids (μg/g DW) in lingonberry leaves, collected in different habitats.

Although harvest time is a critical factor for the good agricultural practice of plants, geographical/location effects, which are closely related to physiological and environmental factors may also profoundly alter the overall phytochemical profile [21]. Hence, we quantified phenolic and triterpenic compounds in the samples collected from different regions in Lithuania (accumulation patterns of particular constituents in lingonberry leaves, collected at different locations, are presented in Supplementary Tables S8–S14, while Figure 5 and Figure 6 represent the variance of sum contents of different subgroups of phenolics and triterpenoids, respectively).

Arbutin, well-known for diuretic, antiseptic properties [42], was the prevailing compound in the phenolic profiles, contributing 46–68% of identified phenolics in all tested lingonberry leaf samples (CV = 17%) with the highest (*p* < 0.05) content determined in a sample collected from Jurgionys forest and 2.4-fold lower level in Pažemys sample (Figure 5). Arbutin-related compounds varied significantly (CV = 41%) among samples from different collecting locations, with predominance in samples from Galvokai, Kernai, and Jurgionys. *V. vitis-idaea* L. var. *leucocarpum* Asch. et Magnus sample from Labanoras (a) forest was notably distinguished by the lowest (*p* < 0.05) content of 2-*O*-caffeoylarbutin and this characteristic seems to be not habitat, but genotype-dependent, because the amount of 2-*O*-caffeoylarbutin in the sample of typical variety from the same forest (Labanoras (b)) was 77-fold higher.

Catechins made up to 14% of total identified phenolics with the greatest amounts in samples from Smėlynė and Apūniškis forests and approx. 3.2-fold lower content in the Bitėnai sample. *V. vitis-idaea* var. *leucocarpum* sample (Labanoras (a)) was also notably distinguished by a very low (40.7 µg/g DW) content of (–)-epicatechin. The distribution of proanthocyanidins was similar to catechins in samples from different collecting locations, ranging between 11,162.3 µg/g DW (Bitėnai sample) and 28,093.3 µg/g DW (Pagramantis sample) with predominant compounds—procyanidin A1 and B3. The richness of these compounds is closely associated with the antioxidant and anticancer properties of lingonberry leaves [8].

The complex of flavonols consisted of flavonol aglycones and glycosides, with the highest average contribution of avicularin (20%), followed by hyperoside (17%) and quercetin-HMG-rhamnoside (16%). The chemical biodiversity of flavonols strongly reflects the metabolic plasticity of plants and their adaptation to local growing conditions [43]. The sum of flavonols was in a range of 2825.7–15,681.0 µg/g DW and predominated in samples of Smėlynė, Tolkūnai, Bakūriškis, and Pažemys (made up to 14% of identified phenolics), whereas the lowest contribution was found in samples of Plunksnuočiai and Bitėnai (about 3% of identified phenolics). Some samples were clearly distinguished by the characteristic contents of particular compounds out of the flavonol group, such as lingonberry leaves from Marcinkonys forest, which were characterized by the highest (*p* < 0.05) level of flavonol aglycones, and the Labanoras (a) sample, which was almost void of lingonberry phytochemical marker—quercetin-HMG-rhamnoside. It may be pointed out that contents of nicotiflorin, astragalin, and rutin were most highly habitat dependent (CV up to 83%), only trace levels of these compounds were found in the Viršilai sample and about 120.2-fold higher content was found in the Smėlynė sample.

The greatest amount of identified phenolic acids, which made up only 1–4% of total identified phenolics and can be considered as minor constituents, was found in a sample of Smėlynė forest (5601.1 µg/g DW), and was 7.1-fold lower in the Bitėnai sample, with the highest variation of predominant chlorogenic acid (CV = 73%). The level of phenolic acids was not clearly genetic-dependent, because significant differences between samples of Labanoras (a) and Labanoras (b) were determined only when assessing contents of *p*-coumaric acid; 2.4-fold lower content in *V. vitis-idaea* var. *leucocarpum* was found, compared to typical variety.

The triterpenoid profile was predominated by neutral triterpenoids with the significantly greatest total amount determined in a sample from Labanoras (a) (6735.1 µg/g DW) and a 4-fold lower content in the typical variety Kūprė sample (Figure 6). The predominant neutral triterpenoid α-amyrin, which demonstrates a strong anti-inflammatory effect [44], accounted for 15 to 51% of total identified triterpenoids in samples from Šakarva and Galvokai, respectively. Interestingly, the samples from Plunksuočiai and Šakarva forest, which were distinguished by the lowest (*p* < 0.05) content of α-amyrin, were characterized by the significantly highest content of β-amyrin, implying that different environmental factors affect the biosynthesis of these compounds. Different biosynthesis pathways of these compounds also should be noted [45]. The highest variance out of neutral triterpenoids was found for friedelin (CV = 121%), whose content was in a range of 54.0–2020.2 µg/g DW, in samples from Šakarva and Kernai, accordingly.

The quantitative profiles of triterpenoid acids were also location-specific with a similar accumulation pattern to neutral triterpenoids. *V. vitis-idaea* var. *leucocarpum* sample was distinguished by the highest (*p* < 0.05) contents of all triterpenoids acids and the total amount of these reached up 3312.3 µg/g DW. Ursolic acid was the prevailing triterpenoid acid in all tested samples with the highest contribution in the Pagramantis sample (29% to total identified triterpenoids). The content ratio of oleanolic and ursolic was found to be from 1:1.2 to 1:5.8 for lingonberry leaves, collected from Labanoras (a) and Apūniškis, respectively. A study by Szakiel et al. [12] showed more constant 1:2–1:2.2 ratios in Finnish and Polish lingonberry leaves. Minor triterpenoid acids, such as maslinic, corosolic, and betulinic acid, varied significantly (CV up to 131%), just trace levels of them were found in the Viršilai sample, while the highest (*p* < 0.05) contents in Labanoras (a) sample, followed by samples from Šilinė and Tolkūnai.

The accumulation pattern of the main sterol of lingonberry leaves—β-sitosterol was quite similar among different samples (CV = 12%) with content ranging between 285.8 and 441.1 µg/g DW in samples collected from Bitėnai and Kūprė, respectively. Despite a determined high level of β-sitosterol (438.7 µg/g DW) in *V. vitis-idaea* var. *leucocarpum* sample, the contribution of this compound to total identified triterpenoids was the lowest (4.2%) and similar to content found in the sample of the typical variety (Labanoras (b)).

Overall, total amounts of identified phenolics ranged from 68,853.8 µg/g up to 144,096.5 µg/g in samples from Bitėnai and Smėlynė, respectively, whereas the highest content of total identified triterpenoids was found in a sample from Labanoras (a) (10,486.1 µg/g DW), followed by 1.6-fold lower content in Šilinė and the lowest amount in Kūprė sample (2937.5 µg/g DW). Although phytochemical markers of lingonberry leaves—arbutin, (+)-catechin, procyanidins B3, A1, avicularin, and α-amyrin—can be considered, some variance in phenolic and triterpenoids profiles was observed, suggesting chemophenetic differences. Our results go beyond the previous report, showing that the content of triterpenoids is strongly influenced by genotype, while habitat differences accounting more for variation in phenolics [46].

#### 2.2.2. Principal Component Analysis of Phenolic and Triterpenic Compounds

**Figure 7:**
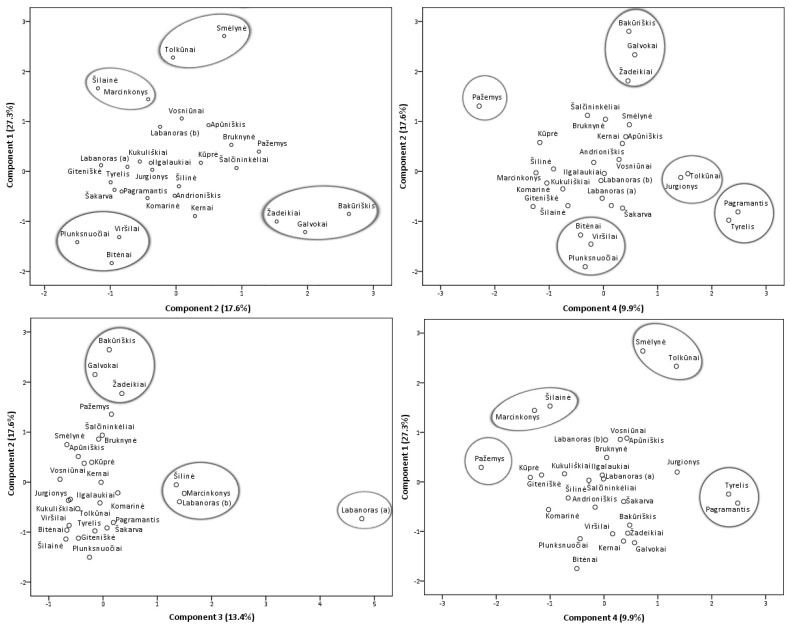
PCA scores estimating contents of phenolic and triterpenic compounds in lingonberry leaves based on their collecting locations.

Principal component analysis (PCA) model was constructed in order to featured similarities and differences between samples from different collecting locations (Figure 7) and additionally, a heatmap (Supplementary Figure S2) was constructed to obtain a preliminary overview. Four principal components, PC1, PC2, PC3, and PC4, explaining 68.2% of the total data variance, were obtained. The first principal component had the greatest influence on the scores of the samples and described 27.3% of the total variance. PC1 differentiated samples containing the highest levels of minor flavonol glycosides (with the strongest correlation with isoquercitrin, astragalin, rutin, and nicotiflorin), flavonol aglycones, (+)-catechin, and procyanidins A1, A4, B3. The second principal component accounted for 17.6% of the total variance and positively correlated with major flavonols of lingonberry leaves—avicularin, hyperoside, quercetin-HMG-rhamnoside, also phenolic acids, mainly neochlorogenic and cryptochlorogenic, betulin, and β-sitosterol. The PC3 component constituted 13.4% of the variance and highly correlated with positive loadings of quercitrin and most triterpenoids (with the strongest correlation to oleanolic acid, erythrodiol, uvaol, and α-amyrin), also negatively correlated with quercetin-HMG-rhamnoside, (–)-epicatechin, and 2-*O*-caffeoylarbutin. The fourth principal component described the lowest part of the total variance (9.9%) and differentiated samples containing the highest contents of arbutin and procyanidins A2, B2, and C1.

The study of Iwanycki Ahlstrand et al. [47] highlighted geographic region, distance, and habitat type as main factors leading to differences in chemical phenotypes of plants. The PCA score plots of samples of lingonberry leaves showed their arrangement into distinct groups and indicated possible different lingonberry chemophenotypes in Lithuania, related to different location conditions. Samples from Tolkūnai and Smėlynė were distanced from all others and were grouped at the positive side of the first and fourth principal components due to high contents of isoquercitrin, rutin, astragalin, and procyanidin C1. Even though these samples were from different regions of Lithuania, locations of these samples were distinguished by higher than average altitude and pH value of soil. On the positive side of PC1, samples from South Lithuania—Marcinkonys and Šilainė were also located due to the predominance of flavonol aglycones. Samples collected from the North or North-East Lithuania, namely Žadeikiai, Galvokai, and Bakūriškis, were located on the positive side plots of the second principal component and the negative side of the first principal component. These samples were distinguished by high loadings of quercetin-HMG-rhamnoside, hyperoside, cryptochlorogenic, neochlorogenic acids but very low contents of rutin, nicotiflorin, and astragalin. Additionally, these samples were characterized by a very low pH of the soil (pH < 3.1). The Plunksnuočiai, Viršilai samples from North-East Lithuania, and the Bitėnai sample from West Lithuania were characterized by lower than average values of macronutrients in soil and were grouped at the negative sides of PC1 and PC2 mainly because of low contents of flavonols, (+)-catechin, and procyanidin B3. A unique sample from Labanoras (a) was located on the right-hand plot of the third and negative side of the second principal components. This sample was clearly distinguished by the highest contents of triterpenoids and quercitrin but only trace levels of quercetin-HMG-rhamnoside, (–)-epicatechin, and 2-*O*-caffeoylarbutin. As far as we know, this is the first report characterizing wild-growing *V. vitis-idaea* var. *leucocarpum* leaves, indicating that this variety is distinguished not only by very low content of anthocyanins in fruits [48] but also by notable differences in phenolic and triterpenoid profiles. Most closely related to this variety was a sample from the same location, Labanoras (b), but with typical variety. Samples from Marcinkonys and Šilinė can be expected to have similar properties due to the positive location of PC3 and negative side of PC2. Lingonberry leaves collected from West Lithuanian forests of Pagramantis and Tyrelis were located at the positive side of the fourth and negative side of second principal components due to the highest levels of procyanidins A2, B2, but low content of hyperoside and cryptochlorogenic acid. One of the highest levels of nitrogen (N) and magnesium (Mg) was found in the soil of these samples. The positive location on PC4 of Tolkūnai and Jurgionys samples from South and South-East Lithuania, respectively, was related to high loadings of arbutin. These samples were also characterized by the highest levels of phosphorus (P) in soil. The Pažemys sample from North-East Lithuania was distanced from all other samples at the negative side of PC4 and positive of PC2, due to having the lowest content of arbutin but the highest level of betulin.

#### 2.2.3. Location and Soil Effects Analysis

Our study showes that samples from different locations differ in phytochemical composition, suggesting that location and edaphic conditions have an impact on the biosynthesis of secondary metabolites. Latitudinal, longitudinal, and altitudinal gradients, which are closely related to environmental factors, in addition to soil fertility and salinity, are factors that may lead plants to better adapt themselves, favor growth and development, and may affect metabolite levels of different geographical origins [21,39].

Latitude, longitude, and altitude have an impact on sunshine duration, temperature and precipitation, hence they indirectly affect the accumulation of secondary metabolites in plants. More precipitation, a decrease in yearly temperatures and sunshine duration at higher latitudes, and lower air temperatures at higher altitudes are expected [27,49]. Negative significant correlations between latitudes of lingonberry leaf collecting locations and amounts of quercetin, kaempferol, astragalin, and nicotiflorin (*r* = −0.621, −0.587, −0.630, and −0.657, respectively, *p* < 0.05) in samples were determined, with other correlations being insignificant. Surprisingly, flavonol aglycones and kaempferol glycosides were the only ones that correlated positively with sunshine duration when analyzing correlations between seasonal factors and identified compounds content in lingonberry leaves throughout the year (2.1.3.), thus supporting higher demand of solar radiation for these compounds.

Since longitudes and altitudes were closely related (*r* = 0.768, *p* < 0.05) they correlated positively with same compounds—catechins and proanthocyanidins—with the strongest correlation between altitudes and contents of (+)-catechin and procyanidin A4 (*r* = 0.769 and 0.723, accordingly, *p* < 0.05). It is noteworthy that these groups of phenolics were also negatively correlated with sunshine duration and temperatures throughout the year (2.1.3.). Additionally, some positive moderate correlations between altitudes of collecting locations and total phenolic content, most quercetin glycosides, and β-sitosterol (*r* = 0.524–0.666, *p* < 0.05) were determined in the present study. Our results match well with previous studies wherein plants growing in higher altitudes and latitudes resulted in higher contents of bioactive compounds in leaves and greater biological activity [50,51].

All plants require different nutrients not only for their growth but also for the biosynthesis of secondary metabolites [39]. Lingonberries tolerate low fertility soils and have lower nutritional requirements compared to many other plants; however, unfavorable conditions can easily lead to leaf damage, poor growth, and yield [2]. As well as a deficit, excessive amounts of nutrients and chemicals may negatively affect plant productivity and cause plant dieback [52]. In the light of the previous report, the level of mineral nutrition in the soil is closely related to the natural habitats of wild flora. *Pinetum Sphagnosum* type of forest was characterized by higher contents of macronutrients, particularly by the highest N level, while commonly found *Pinetum vaccinio-myrtillosum* was characterized by one of the lowest contents of N, P, potassium (K), and calcium (Ca) in soil [23]. Since the roots of *Vaccinium* species are known to be in symbiotic association with ericoid mycorrhizal fungi and form ericoid mycorrhizas [53], this relationship also can be one of the factors positively influencing secondary metabolite production in lingonberry leaves. In the present study, the strongest correlation to total macronutrients level was determined for arbutin and its derivatives, with the most important macronutrient being phosphorus (*r* = 0.727, *p* < 0.05). Additionally, strong positive correlations were found between N level in soil and procyanidin A2 (*r* = 0.621, *p* < 0.05) and between Mg and (–)-epicatechin (*r* = 0.694, *p* < 0.05) content in lingonberry leaves. These macronutrients are one of the key factors, which play an important role in plant growth and development: N is attributed as the main constituent, involved in secondary metabolite metabolism, P plays a role in energy transfer, while Mg is required for photosynthesis and protein synthesis [21,52]. Research by Karlsons et al. [2] has provided evidence for a positive correlation between lingonberry plant yield and fertilization with macronutrients and indicated that fertilization can be used as a practical method to provide adequate particular component concentrations in lingonberry leaves.

Lingonberries have been reported to grow in acidic soils from pH 2.9 to 5.5, with the most favorable conditions ranging from pH 3.5 to 4.5 [54,55]. Even though pH may influence the availability of nutrients and regulate the growth of secondary metabolites [2], no significant correlations were found between soil pH and bioactive compounds content in lingonberry samples in the present study, probably due to the relatively narrow pH range. Of all collecting locations, just Kukuliškiai stood out the most in terms of pH (4.8), and the sample from this location was distinguished by lower than average values of most identified phenolics and triterpenoids.

Electrical conductivity (EC) reflects the content of mineral salts, especially sodium (Na) and chloride (Cl) levels in the soil. Excessive salinization is a major factor contributing to the loss of productivity of cultivated soils and can decrease the uptake of water and nutrients, reduce growth and photosynthesis level in the plants, and can also cause toxic effects like oxidative stress and dehydration of plant cells, and thus reduce the content of secondary metabolites [56,57]. Electrical conductivity in our study negatively correlated with most identified compounds; however, correlations were not significant. Only a chloride level, as related to the electrical conductivity factor, significantly correlated with flavonols, with the strongest negative correlations with contents of rutin and quercetin (*r* up to –0.629, *p* < 0.05). Furthermore, a noticeable lowest electrical conductivity and chloride level was found in the soil of the Smėlynė sample, which was distinguished by the highest total phenolics content. Overall, this suggests that chloride-free fertilizers should be chosen for lingonberries growing to avoid salt stress [39].

## 3. Materials and Methods

### 3.1. Chemicals

The following substances and solvents were used in the study: procyanidins B1, B2, C1, A1, A2, A4, (+)-catechin, (–)-epicatechin, quercetin, quercitrin (quercetin-3-*O*-rhamnoside), isoquercitrin (quercetin-3-*O*-glucoside), avicularin (quercetin-3-*O*-arabinofuranoside), guaiaverin (quercetin-3-*O*-arabinopyranoside), rutin (quercetin-3-*O*-rutinose), reynoutrin (quercetin-3-*O*-xyloside), kaempferol, nicotiflorin (kaempferol-3-*O*-rutinoside), afzelin (kaempferol-3-*O*-rhamnoside), astragalin (kaempferol-3-*O*-glucoside), chlorogenic acid (3-*O*-caffeoylquinic acid), neochlorogenic acid (5-*O*-caffeoylquinic acid), cryptochlorogenic acid (4-*O*-caffeoylquinic acid), *p*-coumaric acid, arbutin, hydroquinone, α-amyrin, β-amyrin, β-sitosterol, lupeol, erythrodiol, maslinic acid, oleanolic acid, acetonitrile, acetone, and methanol, which were purchased from Sigma–Aldrich (Steinheim, Germany); hyperoside (quercetin-3-*O*-galactoside), procyanidin B3, uvaol, friedelin, 6″-*O*-acetylisoquercitrin (quercetin-3-*O*-(6″-acetylglucoside)), betulin, and betulinic and corosolic acids from Extrasynthese (Genay, France); ursolic acid from Carl Roth (Karlsruhe, Germany); and trifluoroacetic acid from Merck (Darmstadt, Germany). All used chemicals were of HPLC grade. Ultrapure water used in this study was prepared with Milli–Q^®^ 180 (Millipore, Bedford, MA, USA) water purification system.

### 3.2. Plant Material and Growing Conditions

Lingonberry leaves were collected from different natural habitats in Lithuania (Table 1, Supplementary Figure S3) from 10 randomly selected shrubs in each habitat at the end of September 2019. Almost all forests were predominated by Scots pine (*Pinus sylvestris* L.); only Šalčininkėliai, Bruknynė, and Šilinė were characterized by European spruce (*Picea abies* (L.) H. Karst.) and Pagramantis by Silver birch (*Betula pendula* Roth) as main tree species. Samples from Labanoras were divided into (a) leaves of unique variety—white-fruited *V. vitis-idaea* var. *leucocarpum*, which is described only in the flora of Lithuania up to date (discovered in 1993 and wild-growing only in this collecting location) and (b) leaves of typical lingonberry variety growing in the same forest plots.

Soil samples of collecting locations were separately taken from the plant root zones in a depth of 0–20 cm, then sieved, air-dried, and analyzed in the Agrochemical Research Laboratory (Lithuanian Research Centre for Agriculture and Forestry). The pH was measured in 1 M KCl extract (1:5, *w/v*), contents of N, Ca, Mg, Cl, and EC were measured in a soil-water extract (1:10, *w/v*), while P and K were analyzed in a 0.5 M acetic acid/sodium acetate buffer (1:10, *w/v*).

Sampling for phenological stage study was conducted at intervals of about 14 days for a period of 12 months (from July to July 2019–2020) in a *Pinetum vaccinio-myrtilosum* type Apuniškis forest (altitude 110 m; 56°00′40.6″ N 25°31′29.4″ E). The environmental factors monitored in this study included average monthly temperature (°C), precipitation (mm), humidity (%), and sunshine duration (h). All data were obtained from the archive of the Lithuanian Hydrometeorological Service under the Ministry of Environment of the Republic of Lithuania and are presented in Supplementary Figure S4. At each time, lingonberry shoots were collected at the same plot (10 m^2^) from 10 shrubs, applying a randomized experimental design. The leaves were detached from the shoots and were grouped into young and old using the following criteria: immature current-year leaves were recognized for their bright green color and their upper position of the stem, whereas fully-matured leaves for a darker color and low position. Collected raw materials were air-dried at room temperature in a dark place and then milled to a fine powder with a Retsch 200 mill (Haan, Germany) and kept in the dark in a sealed containers until extraction. Residual moisture of ground samples was determined by moisture analyzer Precisa 310M (Dietikon, Switzerland) and all further results were expressed as DW.

### 3.3. Preparation of Extracts

Extraction procedures were performed in triplicate (*n* = 3) for each sample. Preparation of phenolic extracts was performed using developed method outlined in our previous study [8] with minor modifications. About 0.2 g of fine powder of dried leaves was extracted with 10 mL 80% *v/v* acetone in water for 25 min at 80 kW and 100% power intensity in an ultrasonic bath Elmasonic P (Singen, Germany). After centrifugation at 3000× *g* for 30 min in a Biofuge Stratos centrifuge (Hanau, Germany), the supernatants were filtered through 0.22 μm pore size membrane filters (Carl Roth GmbH, Karlsruhe, Germany) and transferred to the dark glass vials.

Extraction of triterpenoids was achieved by extracting 1 g of leaf powder with 10 mL of methanol via ultrasound-assisted extraction for 25 min, followed by centrifugation at 3000× *g* for 30 min [58]. Before the injection into the HPLC system, the extracts were filtered by using 0.22 μm pore size membrane filters.

### 3.4. HPLC-PDA Conditions

Analysis of phenolic and triterpenic compounds was conducted with HPLC-PDA (Waters e2695 Alliance system, Waters, Milford, MA, USA) system. The identification was made by comparison of retention times and spectra with those of commercially available standard compounds. For quantification, linear regression models were obtained using the standard dilution method. Validation data and representative chromatograms of methods presented below are given in our previous papers [8,58].

For analysis of phenolic compounds, an ACE Super C18 (250 mm × 4.6 mm, 3 µm) reversed-phase column (ACT, Aberdeen, UK), maintained at 35 °C was used. The mobile phase consisted of 0.1% trifluoroacetic acid (eluent A) and acetonitrile (eluent B) with gradient elution formed as follows: 0 min, 90% A; 0–40 min, 70% A; 40–60 min, 30% A; 60–64 min, 10% A; 64–70 min, 90% A. The flow rate was 0.5 mL/min, and the volume of analyzed extract–10 µL.

For evaluation of triterpenoid acids (maslinic, corosolic, betulinic, oleanolic, ursolic acids) and neutral triterpenoids with chromophores (betulin, erythrodiol, uvaol), 10 µL of sample extracts were injected on an ACE C18 (150 mm × 4.6 mm, 3 µm) reversed-phase column (ACT, Aberdeen, UK) with column temperature set at 20 °C. Acetonitrile and water (89:11, *v/v*) were used as mobile phases, delivered in the isocratic mode at a flow rate of 0.7 mL/min.

Chromatographic separation of neutral triterpenoids, which lack chromophores (lupeol, α-amyrin, β-amyrin, friedelin) and phytosterol (β-sitosterol) was carried out on the same ACE C18 (150 mm × 4.6 mm, 3 µm) reversed-phase column with isocratic elution mode, consisting of acetonitrile and methanol (10:90, *v/v*) at a flow rate of 1 mL/min. The sample injection volume was 10 µL, and the column temperature was maintained at 35 °C.

### 3.5. Statistical Analysis

All of the analyses were done in triplicate, and the data were expressed as means ± standard deviation (SD). The significant differences between extracts of lingonberry leaves were calculated by one-way ANOVA, followed by Tukey post hoc comparison test. PCA and HCA, using the nearest neighbor cluster method with squared Euclidean distances, were performed to confirm differences and similarities between samples. Correlations were tested by using the Pearson correlation test. The *p*-values less than 0.05 were considered statistically significant. All statistical calculations were performed using IBM SPSS Statistics version 26.0 package and Microsoft Office Excel 2016 software.

## 4. Conclusions

Differences in physiological and climatic conditions along with different geographical locations have an impact on the adaptation of lingonberry leaves, with variation in phytochemical properties. Our results provided evidence for continuous biosynthesis and storage of 30 identified phenolic and 13 triterpenic compounds throughout the year, and possible seasonal availability of lingonberry leaves. Results also indicated that harsh weather conditions and cold acclimation have a positive impact on the content of most compounds, so lingonberry leaves should preferentially be collected during the autumn–first half of spring. Different lingonberry chemophenotypes, distinguished by particular levels of secondary metabolites in a correlation to habitat differences, have been found in Lithuania, thus indicating that macronutrient status, soil quality, light, temperature, and humidity regimes may be employed to manipulate the phenolic and triterpenoid content in lingonberry leaves. However, it is worthwhile noting that observed variance of phytochemical profiles, should be understood not strictly based on one factor, but as the result of the complex of biotic and abiotic ones.

## Figures and Tables

**Table 1:** Description of lingonberry leaves collecting locations and soil data.

Forest	Location Parameters			Soil Parameters							
	Latitude, °	Longitude, °	Altitude, m	pH_KCl_	N, mg/L	P, mg/L	K, mg/L	Ca, mg/L	Mg, mg/L	Cl, mg/L	EC, mS/cm
Žadeikiai	56.01	24.47	52	3.0	6	2	36	12	5	18	0.17
Vosniūnai	55.90	24.69	77	3.1	27	7	65	41	14	18	0.67
Galvokai	56.06	24.89	80	3.1	40	3	29	19	7	23	0.38
Viršilai	56.09	25.31	102	3.1	28	7	50	55	8	18	0.64
Plunksnuočiai	56.08	25.49	107	3.4	16	2	32	15	5	18	0.36
Ilgalaukiai	55.99	25.43	107	2.9	30	8	51	57	8	19	0.47
Apūniškis	56.01	25.52	110	4.2	116	6	48	60	23	18	1.10
Bakūriškis	56.06	25.68	123	2.9	39	10	66	61	15	23	0.83
Andrioniškis	55.60	25.04	111	3.6	50	10	91	24	6	18	0.56
Pažemys	55.66	26.00	171	3.5	114	3	87	23	9	23	0.94
Giteniškė	55.60	26.13	173	4.1	7	0.2	20	9	4	18	0.19
Smėlynė	55.40	26.15	187	3.9	60	6	64	26	5	15	0.14
Šakarva	55.31	26.06	149	3.6	6	2	31	10	4	23	1.68
Labanoras (a,b)	55.16	25.81	165	3.3	12	0.9	20	12	4	18	0.27
Šalčininkėliai	54.38	25.38	171	3.1	73	17	101	24	9	20	0.90
Bruknynė	54.35	25.39	189	3.2	80	17	113	26	11	20	0.95
Jurgionys	54.46	24.50	139	3.3	89	28	116	25	18	22	1.11
Tolkūnai	54.27	24.41	145	3.8	70	18	88	31	12	19	0.52
Marcinkonys	54.07	24.44	137	3.2	9	1	26	12	4	12	0.21
Šilainė	54.08	23.71	134	2.9	11	2	30	17	6	23	0.29
Bitėnai	55.06	22.05	22	3.2	27	4	36	13	6	17	0.99
Šilinė	55.19	22.31	39	3.3	57	12	45	13	6	18	0.25
Komarinė	55.19	22.45	47	2.9	48	6	56	22	8	18	0.62
Pagramantis	55.39	22.22	89	3.2	105	12	76	32	15	20	0.85
Tyrelis	55.32	22.17	59	3.2	166	10	96	49	16	20	0.97
Kūprė	55.58	22.82	145	3.8	8	0.4	15	9	4	23	0.22
Kukuliškiai	55.78	21.09	34	4.8	52	7	61	10	13	20	1.70
Kernai	56.23	21.49	44	4.0	185	13	113	76	27	23	1.13

## Data Availability

All data generated during this study are included in this article.

## Supplementary Materials

The following are available online at https://www.mdpi.com/article/10.3390/plants10101986/s1, Table S1: Contents of simple phenolics and A-type proanthocyanidins (μg/g DW ± SD) in young and old lingonberry leaves, collected throughout one year, Table S2: Contents of catechins and B-type proanthocyanidins (μg/g DW ± SD) in young and old lingonberry leaves, collected throughout one year, Table S3: Contents of flavonol aglycones and phenolic acids (μg/g DW ± SD) in young and old lingonberry leaves, collected throughout one year, Table S4: Contents of kaempferol and quercetin glycosides (μg/g DW ± SD) in young and old lingonberry leaves, collected throughout one year, Table S5: Contents of quercetin glycosides (μg/g DW ± SD) in young and old lingonberry leaves, collected throughout one year, Table S6: Contents of triterpenoid acids and sterols (μg/g DW ± SD) in young and old lingonberry leaves, collected throughout one year, Table S7: Contents of neutral triterpenoids (μg/g DW ± SD) in young and old lingonberry leaves, collected throughout one year, Figure S1: Variation of total identified secondary metabolites (μg/g DW) in lingonberry leaves, collected throughout one year, Table S8: Contents of simple phenolics and A-type proanthocyanidins (μg/g DW ± SD) in lingonberry leaves, collected at different locations, Table S9: Contents of catechins and B-type proanthocyanidins (μg/g DW ± SD) in lingonberry leaves, collected at different locations, Table S10: Contents of flavonol aglycones and phenolic acids (μg/g DW ± SD) in lingonberry leaves, collected at different locations, Table S11: Contents of kaempferol and quercetin glycosides (μg/g DW ± SD) in lingonberry leaves, collected at different locations, Table S12: Contents of quercetin glycosides (μg/g DW ± SD) in lingonberry leaves, collected at different locations, Table S13: Contents of triterpenoid acids and sterols (μg/g DW ± SD) in lingonberry leaves, collected at different locations, Table S14: Contents of neutral triterpenoids (μg/g DW ± SD) in lingonberry leaves, collected at different locations, Figure S2: Heatmap estimating contents of phenolic and triterpenic compounds in lingonberry leaves based on their collecting locations, Figure S3: Collecting locations of lingonberry leaves samples in Lithuania, Figure S4: Dynamics of meteorological factors during one year testing period in Lithuania.

## Appendix A

Abbreviations of months that were included in Figure 1, Figure 2 and Figure 3, Supplementary Figures S1 and S4, Supplementary Tables S1–S7: January (Jan.), February (Feb.), March (Mar.), April (Apr.), May (not abbreviated), June (Jun.), July (Jul.), August (Aug.), September (Sep.), October (Oct.), November (Nov.), December (Dec.).

## References

[1] Debnath S.C., Arigundam U. (2020). In vitro propagation strategies of medicinally important berry crop, lingonberry (*Vaccinium vitis-idaea* L.). Agronomy.

[2] Karlsons A., Tomsone S., Lazdāne M., Osvalde A. (2021). Effect of fertilization on growth of lingonberry (*Vaccinium vitis-idaea* L.). Agron. Res..

[3] Ross K.A., Godfrey D., Fukumoto L. (2015). The chemical composition, antioxidant activity and α-glucosidase inhibitory activity of water-extractable polysaccharide conjugates from northern manitoba lingonberry. Cogent Food Agric..

[4] Ștefănescu B.E., Szabo K., Mocan A., Crişan G. (2019). Phenolic compounds from five Ericaceae species leaves and their related bioavailability and health benefits. Molecules.

[5] Kowalska K., Dembczyński R., Gołąbek A., Olkowicz M., Olejnik A. (2021). ROS modulating effects of lingonberry (*Vaccinium vitis-idaea* L.) polyphenols on obese adipocyte hypertrophy and vascular endothelial dysfunction. Nutrients.

[6] Ferlemi A.-V., Lamari F.N. (2016). Berry leaves: An alternative source of bioactive natural products of nutritional and medicinal value. Antioxidants.

[7] Páscoa R.N.M.J., Gomes M.J., Sousa C. (2019). Antioxidant activity of blueberry (*Vaccinium* spp.) cultivar leaves: Differences across the vegetative stage and the application of near infrared spectroscopy. Molecules.

[8] Vilkickyte G., Raudone L., Petrikaite V. (2020). Phenolic fractions from *Vaccinium vitis-idaea* L. and their antioxidant and anticancer activities assessment. Antioxidants.

[9] Alam Z., Roncal J., Peña-Castillo L. (2018). Genetic variation associated with healthy traits and environmental conditions in *Vaccinium vitis-idaea* L.. BMC Genom..

[10] Bujor O.-C., Tanase C., Popa M.E. (2019). Phenolic antioxidants in aerial parts of wild *Vaccinium* species: Towards pharmaceutical and biological properties. Antioxidants.

[11] Ștefănescu B.-E., Călinoiu L.F., Ranga F., Fetea F., Mocan A., Vodnar D.C., Crișan G. (2020). Chemical composition and biological activities of the Nord-West Romanian wild bilberry (*Vaccinium myrtillus* L.) and lingonberry (*Vaccinium vitis-idaea* L.) leaves. Antioxidants.

[12] Szakiel A., Pączkowski C., Koivuniemi H., Huttunen S. (2012). Comparison of the triterpenoid content of berries and leaves of lingonberry *Vaccinium vitis-idaea* L. from Finland and Poland. J. Agric. Food Chem..

[13] Tian Y., Liimatainen J., Alanne A.-L., Lindstedt A., Liu P., Sinkkonen J., Kallio H., Yang B. (2017). Phenolic compounds extracted by acidic aqueous ethanol from berries and leaves of different berry plants. Food Chem..

[14] Isah T. (2019). Stress and defense responses in plant secondary metabolites production. Biol. Res..

[15] Vrancheva R., Ivanov I., Dincheva I., Badjakov I., Pavlov A. (2021). Triterpenoids and other non-polar compounds in leaves of wild and cultivated *Vaccinium* species. Plants.

[16] Chen Y., Zhu Z., Guo Q., Zhang L., Zhang X. (2012). Variation in concentrations of major bioactive compounds in *Prunella vulgaris* L. related to plant parts and phenological stages. Biol. Res..

[17] Radusiene J., Karpaviciene B., Stanius Ž. (2012). Effect of external and internal factors on secondary metabolites accumulation in St. John’s Worth. Bot. Lith..

[18] Li Y., Kong D., Fu Y., Sussman M.R., Wu H. (2020). The effect of developmental and environmental factors on secondary metabolites in medicinal plants. Plant Physiol. Biochem..

[19] Bergman M.E., Davis B., Phillips M.A. (2019). medically useful plant terpenoids: Biosynthesis, occurrence, and mechanism of action. Molecules.

[20] Obata T. (2019). Metabolons in plant primary and secondary metabolism. Phytochem. Rev..

[21] Verma N., Shukla S. (2015). Impact of various factors responsible for fluctuation in plant secondary metabolites. J. Appl. Res. Med. Aromat. Plants.

[22] Alqahtani A., Tongkao-on W., Li K.M., Razmovski-Naumovski V., Chan K., Li G.Q. (2015). Seasonal variation of triterpenes and phenolic compounds in Australian *Centella asiatica* (L.) Urb. Phytochem. Anal. PCA.

[23] Bandzaitiene Z., Daubaras R., Labokas J. (2007). Brukne: Vaccinium vitis-idaea L..

[24] Bujor O.-C., Ginies C., Popa V.I., Dufour C. (2018). Phenolic compounds and antioxidant activity of lingonberry (*Vaccinium vitis-idaea* L.) leaf, stem and fruit at different harvest periods. Food Chem..

[25] Bujor O.-C., Le Bourvellec C., Volf I., Popa V.I., Dufour C. (2016). Seasonal variations of the phenolic constituents in bilberry (*Vaccinium myrtillus* L.) leaves, stems and fruits, and their antioxidant activity. Food Chem..

[26] Sommavilla V., Haidacher-Gasser D., Sgarbossa M., Zidorn C. (2012). Seasonal variation in phenolics in leaves of *Celtis australis* (Cannabaceae). Biochem. Syst. Ecol..

[27] Solanki T., Aphalo P.J., Neimane S., Hartikainen S.M., Pieristè M., Shapiguzov A., Porcar-Castell A., Atherton J., Heikkilä A., Robson T.M. (2019). UV-screening and springtime recovery of photosynthetic capacity in leaves of *Vaccinium vitis-idaea* above and below the snow pack. Plant Physiol. Biochem..

[28] Rahajanirina V., Faramalala M., Edmond R., Zebrowski C., Leong J., Tsy J.-M., Danthu P. (2016). Effects of harvest frequency on leaf biomass and triterpenoid content of *Centella asiatica* (L.) Urb from Madagascar. J. Med. Biol. Res..

[29] Dashbaldan S., Becker R., Pączkowski C., Szakiel A. (2019). Various patterns of composition and accumulation of steroids and triterpenoids in cuticular waxes from screened Ericaceae and Caprifoliaceae berries during fruit development. Molecules.

[30] Olofsson J., Ericson L., Torp M., Stark S., Baxter R. (2011). Carbon balance of Arctic tundra under increased snow cover mediated by a plant pathogen. Nat. Clim. Chang..

[31] Shitan N. (2016). Secondary metabolites in plants: Transport and self-tolerance mechanisms. Biosci. Biotechnol. Biochem..

[32] Murthy H.N., Lee E.-J., Paek K.-Y. (2014). Production of secondary metabolites from cell and organ cultures: Strategies and approaches for biomass improvement and metabolite accumulation. Plant Cell Tissue Organ Cult. PCTOC.

[33] Ribeiro D.A., Camilo C.J., de Fátima Alves Nonato C., Rodrigues F.F.G., Menezes I.R.A., Ribeiro-Filho J., Xiao J., de Almeida Souza M.M., da Costa J.G.M. (2020). Influence of seasonal variation on phenolic content and in vitro antioxidant activity of *Secondatia floribunda* A. DC. (Apocynaceae). Food Chem..

[34] He M., He C.-Q., Ding N.-Z. (2018). Abiotic stresses: General defenses of land plants and chances for engineering multistress tolerance. Front. Plant Sci..

[35] Bertolucci S.K.V., Pereira A.B.D., Pinto J.E.B.P., Oliveira A.B., Braga F.C. (2013). Seasonal variation on the contents of coumarin and kaurane-type diterpenes in *Mikania laevigata* and *M. glomerata* leaves under different shade levels. Chem. Biodivers..

[36] Taylor A.O. (1965). Some Effects of Photoperiod on the biosynthesis of phenylpropane derivatives in *Xanthium*. Plant Physiol..

[37] Wu J., Drappier J., Hilbert G., Guillaumie S., Dai Z., Geny-Denis L., Delrot S., Darriet P., Thibon C., Pieri P. (2019). The effects of a moderate grape temperature increase on berry secondary metabolites. OENO One.

[38] Vallat A., Gu H., Dorn S. (2005). How rainfall, relative humidity and temperature influence volatile emissions from apple trees in situ. Phytochemistry.

[39] Yang L., Wen K.-S., Ruan X., Zhao Y.-X., Wei F., Wang Q. (2018). Response of plant secondary metabolites to environmental factors. Molecules.

[40] Kirakosyan A., Kaufman P., Warber S., Zick S., Aaronson K., Bolling S., Chul Chang S. (2004). Applied environmental stresses to enhance the levels of polyphenolics in leaves of hawthorn plants. Physiol. Plant..

[41] Oksanen E., Lihavainen J., Keinänen M., Keski-Saari S., Kontunen-Soppela S., Sellin A., Sõber A., Cánovas F.M., Lüttge U., Matyssek R., Pretzsch H. (2019). Northern forest trees under increasing atmospheric humidity. Progress in Botany.

[42] Migas P., Krauze-Baranowska M. (2015). The significance of arbutin and its derivatives in therapy and cosmetics. Phytochem. Lett..

[43] Scarano A., Chieppa M., Santino A. (2018). Looking at flavonoid biodiversity in horticultural crops: A colored mine with nutritional benefits. Plants.

[44] Okoye N.N., Ajaghaku D.L., Okeke H.N., Ilodigwe E.E., Nworu C.S., Okoye F.B.C. (2014). beta-Amyrin and alpha-amyrin acetate isolated from the stem bark of *Alstonia boonei* display profound anti-inflammatory activity. Pharm. Biol..

[45] Choi H.S., Han J.Y., Choi Y.E. (2020). Identification of triterpenes and functional characterization of oxidosqualene cyclases involved in triterpene biosynthesis in lettuce (*Lactuca sativa*). Plant Sci..

[46] Sun C., Shang X., Ding H., Cao Y., Fang S. (2020). Natural variations in flavonoids and triterpenoids of *Cyclocarya paliurus* leaves. J. For. Res..

[47] Iwanycki Ahlstrand N., Havskov Reghev N., Markussen B., Bruun Hansen H.C., Eiriksson F.F., Thorsteinsdóttir M., Rønsted N., Barnes C.J. (2018). Untargeted metabolic profiling reveals geography as the strongest predictor of metabolic phenotypes of a cosmopolitan weed. Ecol. Evol..

[48] Vilkickyte G., Motiekaityte V., Vainoriene R., Liaudanskas M., Raudone L. (2021). Development, validation, and application of UPLC-PDA method for anthocyanins profiling in *Vaccinium* L. berries. J. Berry Res..

[49] Guo L., Wang S., Zhang J., Yang G., Zhao M., Ma W., Zhang X., Li X., Han B., Chen N. (2013). Effects of ecological factors on secondary metabolites and inorganic elements of *Scutellaria baicalensis* and analysis of geoherblism. Sci. China Life Sci..

[50] Ullah N., Khurram M., Amin M., Khan T., Khayyam S., Farhat A., Khan F., Najeeb U., Ullah S. (2012). Impact of geographical locations on *Mentha spicata* antibacterial activities. J. Med. Plants Res..

[51] Vyas P., Curran N., Igamberdiev A., Debnath S. (2015). Antioxidant properties of lingonberry (*Vaccinium vitis-idaea* L.) leaves within a set of wild clones and cultivars. Can. J. Plant Sci..

[52] Hawkesford M., Horst W., Kichey T., Lambers H., Schjoerring J., Møller I.S., White P. (2012). Marschner’s Mineral Nutrition of Higher Plants.

[53] Baba T., Hirose D., Sasaki N., Watanabe N., Kobayashi N., Kurashige Y., Karimi F., Ban T. (2016). Mycorrhizal formation and diversity of endophytic fungi in hair roots of *Vaccinium oldhamii* Miq. in Japan. Microbes Environ..

[54] Paal T. (2006). Lingonberry (*Vaccinium vitis-idaea* L.) research in Estonia: An overview. Acta Hortic..

[55] Šimala D. (2004). Some experiments on the ecological cultivation of the lingonberry (*Vaccinium vitis-idaea* L.) in a mountainous region of Slovakia. J. Fruit Ornam. Plant Res..

[56] Machado R.M.A., Serralheiro R.P. (2017). Soil salinity: Effect on vegetable crop growth. management practices to prevent and mitigate soil salinization. Horticulturae.

[57] Shrivastava P., Kumar R. (2015). Soil Salinity: A serious environmental issue and plant growth promoting bacteria as one of the tools for its alleviation. Saudi J. Biol. Sci..

[58] Vilkickyte G., Raudone L. (2021). Optimization, validation and application of HPLC-PDA methods for quantification of triterpenoids in *Vaccinium vitis-idaea* L.. Molecules.

