# Supplementary material for: Phenological and Geographical Effects on Phenolic and Triterpenoid Content in Vaccinium vitis-idaea L. Leaves
Source: Plants (Basel). 2021 Sep 23;10(10):1986. doi: 10.3390/plants10101986 (PMC8539284; doi:10.3390/plants10101986)
Supplement: Supplementary file 1 [file plants-10-01986-s001.zip › plants-1376200-supplementary.pdf]

**Table S1.** Contents of simple phenolics and A-type proanthocyanidins (μg/g DW ± SD) in young and old lingonberry leaves, collected throughout one year.

| **Date** | **Arbutin** | | **Hydroquinone** | | **2-*O*-Caffeoylarbutin** | | **Procyanidin A1** | | **Procyanidin A2** | | **Procyanidin A4** | |
|---|---|---|---|---|---|---|---|---|---|---|---|---|
| | **Young leaves** | **Old leaves** | **Young leaves** | **Old leaves** | **Young leaves** | **Old leaves** | **Young leaves** | **Old leaves** | **Young leaves** | **Old leaves** | **Young leaves** | **Old leaves** |
| **Jan. 11** | 64151.3 ± 1090.6* | 67158.7 ± 2552.0 | 22.5 ± 0.8 | 0.4 ± 0.0 | 3516.5 ± 42.2 | 2859.6 ± 108.7 | 7206.4 ± 158.5* | 5917.4 ± 59.2 | 987.7 ± 15.8* | 1310.4 ± 32.8* | 154.6 ± 8.0* | 136.1 ± 1.4* |
| **Jan. 24** | 60261.1 ± 2531.0 | 67049.2 ± 2413.8 | 5.3 ± 0.2 | 2.9 ± 0.1 | 3510.2 ± 147.4 | 3079.4 ± 110.9 | 6449.7 ± 141.9 | 5666.3 ± 164.3 | 923.5 ± 30.5 | 964.6 ± 28.0 | 141.5 ± 3.1* | 88.7 ± 2.6 |
| **Feb. 7** | 60655.6 ± 1941.0 | 68945.1 ± 1999.4 | 8.6 ± 0.3 | 5.3 ± 0.2 | 3373.3 ± 107.9 | 3260.7 ± 94.6* | 6344.5 ± 190.3 | 5601.1 ± 61.6 | 826.8 ± 27.3 | 988.7 ± 28.7 | 107.9 ± 3.2 | 88.8 ± 1.0 |
| **Feb. 21** | 65851.7 ± 1909.7* | 62239.1 ± 2053.9 | 55.6 ± 2.2 | 15.2 ± 0.5 | 3587.6 ± 104.0 | 3069.7 ± 101.3 | 6381.4 ± 287.2 | 5505.1 ± 187.2 | 830.9 ± 24.1 | 1033.4 ±34.1 | 98.7 ± 4.4 | 91.5 ± 3.1 |
| **Mar. 7** | 59887.9 ± 2215.9 | 61940.2 ± 1114.9 | 29.8 ± 1.1 | 15.5 ± 0.3 | 3634.7 ± 134.5 | 2629.1 ± 23.7 | 5387.1 ± 188.5 | 3970.7 ± 87.4 | 849.6 ± 14.4 | 910.5 ± 16.4 | 98.9 ± 3.5 | 77.7 ± 1.7 |
| **Mar. 21** | 62091.3 ± 1862.7 | 61380.8 ± 1534.5 | 41.7 ± 1.2 | 9.5 ± 0.2 | 3623.7 ± 108.7 | 2604.0 ± 65.1 | 5721.6 ± 206.0 | 4065.2 ± 182.9 | 853.4 ± 25.6 | 951.2 ± 23.8 | 99.8 ± 3.6 | 76.9 ± 3.5 |
| **Apr. 4** | 60535.3 ± 1816.1 | 58170.2 ± 988.9 | 31.5 ± 0.9 | 15.1 ± 0.3 | 3600.1 ± 108.0 | 2608.4 ± 70.4 | 5398.5 ± 118.8 | 4137.1 ± 157.2 | 750.6 ± 22.5 | 941.6 ± 23.5 | 79.9 ± 2.6 | 67.2 ± 2.6 |
| **Apr. 17** | 58508.5 ± 1462.7 | 62416.8 ± 1872.5 | 17.0 ± 0.4 | 8.4 ± 0.3 | 3618.6 ± 90.5 | 2669.1 ± 80.1 | 4631.0 ± 194.5 | 4355.2 ± 156.8 | 729.9 ± 7.3 | 951.4 ± 16.2 | 85.0 ± 3.6 | 78.5 ± 2.8 |
| **May 1** | 22669.7 ± 430.7 | 61517.3 ± 1845.5 | 2.8 ± 0.1 | 9.2 ± 0.3 | 3293.5 ± 72.5 | 2261.9 ± 13.6 | 2040.4 ± 65.3 | 4172.3 ± 121.0 | 366.8 ± 9.2 | 1006.2 ± 30.2 | 15.7 ± 0.5 | 86.9 ± 2.5 |
| **May 15** | 26987.7 ± 593.7 | 60209.7 ± 1746.1 | 16.0 ± 0.4 | 10.4 ± 0.2 | 3569.3 ± 78.5 | 2343.1 ± 67.9 | 2164.6 ± 41.1 | 4158.9 ± 95.7 | 416.6 ± 7.9 | 1049.4 ± 31.5 | 20.9 ± 0.4 | 90.2 ± 3.0 |
| **May 29** | 28991.5 ± 869.7 | 60189.2 ± 1986.2 | 169.6 ± 5.9 | 115.6 ± 3.5 | 3836.0 ± 115.1 | 1933.9 ± 44.5 | 1950.8 ± 60.5 | 4302.3 ± 94.7 | 402.9 ± 10.1 | 1086.5 ± 25.0 | 19.7 ± 0.6 | 84.0 ± 1.8 |
| **Jun. 13** | 29163.1 ± 904.1 | 63852.9 ± 2873.4 | 434.8 ± 17.8* | 154.9 ± 7.0 | 4158.3 ± 128.9 | 2333.4 ± 105.0 | 2005.4 ± 20.1 | 4133.1 ± 103.3 | 457.0 ± 5.0 | 1132.1 ± 53.2 | 22.5 ± 0.2 | 86.4 ± 3.0 |
| **Jun. 28** | 30708.5 ± 1044.1 | 62728.7 ± 2195.5 | 372.8 ± 12.7 | 144.0 ± 5.0 | 4267.4 ± 145.1 | 2434.9 ± 85.2 | 2043.9 ± 45.0 | 4277.1 ± 141.1 | 305.4 ± 10.4 | 1079.0 ± 18.3 | 21.5 ± 0.5 | 86.8 ± 3.7 |
| **Jul. 15** | 34773.3 ± 973.7 | 63875.4 ± 2235.6 | 370.7 ± 10.4 | 129.6 ± 4.5 | 4602.9 ± 197.9 | 2511.9 ± 105.5 | 2104.7 ± 71.6 | 4397.5 ± 70.4 | 386.5 ± 7.0 | 1090.5 ± 16.4 | 29.2 ± 1.0 | 81.7 ± 2.1 |
| **Jul. 29** | 35342.4 ± 565.5 | 65875.5 ± 1778.6 | 368.6 ± 9.6 | 150.2 ± 4.1 | 4646.7 ± 199.8 | 2728.9 ± 87.3 | 2160.3 ± 23.8 | 4384.1 ± 65.8 | 367.2 ± 10.7 | 1117.8 ± 33.5 | 28.3 ± 0.3 | 85.3 ± 1.3 |
| **Aug. 11** | 41493.8 ± 1286.3 | 64573.3 ± 2324.6 | 371.8 ± 11.5 | 143.6 ± 5.2 | 5045.3 ± 196.8 | 2665.5 ± 96.0 | 3777.7 ± 117.1 | 4455.8 ± 187.1 | 484.1 ± 12.1 | 1137.7 ± 26.2 | 28.6 ± 0.9 | 80.1 ± 3.4 |
| **Aug. 24** | 41701.9 ± 625.5 | 72877.4 ± 2405.0* | 384.4 ± 5.8 | 174.3 ± 5.8 | 5724.7 ± 143.1* | 2659.6 ± 87.8 | 4059.4 ± 105.5 | 4461.6 ± 142.8 | 448.6 ± 6.7 | 1032.6 ± 16.5 | 32.1 ± 0.8 | 84.0 ± 2.7 |
| **Sep. 6** | 55664.9 ± 1391.6 | 71075.8 ± 2132.3* | 286.6 ± 7.2 | 177.3 ± 5.3 | 4747.6 ± 118.7 | 2822.3 ± 84.7 | 4715.0 ± 89.6 | 4954.8 ± 143.7 | 474.3 ± 16.6 | 1058.9 ± 22.2 | 35.5 ± 0.7 | 79.3 ± 2.0 |
| **Sep. 20** | 66370.7 ± 1858.4* | 71798.8 ± 3015.5* | 277.6 ± 7.8 | 191.6 ± 8.0* | 4474.0 ± 125.3 | 2891.0 ± 121.4 | 4998.0 ± 115.0 | 4744.3 ± 128.1 | 591.0 ± 17.1 | 1082.7 ± 34.6 | 87.7 ± 2.0 | 88.5 ± 3.3 |
| **Oct. 4** | 64099.5 ± 1025.6* | 71628.9 ± 1934.0* | 222.0 ± 3.6 | 89.4 ± 2.4 | 3956.3 ± 63.3 | 2794.8 ± 75.5 | 5014.2 ± 90.3 | 4895.6 ± 122.4 | 591.3 ± 9.5 | 1160.0 ± 44.1 | 87.7 ± 1.6 | 91.7 ± 2.3 |
| **Oct. 19** | 63837.3 ± 1979.0* | 71893.0 ± 2588.1* | 225.3 ± 7.0 | 39.4 ± 1.8 | 4066.1 ± 126.0 | 2756.9 ± 71.7 | 5829.2 ± 180.7 | 5042.6 ± 95.8 | 815.5 ± 34.3 | 1071.0 ± 46.1 | 96.5 ± 2.0 | 87.1 ± 1.7 |
| **Nov. 1** | 70777.2 ± 1061.7* | 73650.4 ± 2430.5* | 54.7 ± 2.5 | 13.2 ± 0.4 | 4058.0 ± 60.9 | 2803.6 ± 92.5 | 6189.3 ± 185.7 | 5087.3 ± 127.2 | 815.3 ± 26.1 | 1077.9 ± 42.0 | 106.6 ± 3.2 | 88.1 ± 2.2 |
| **Nov. 16** | 72168.0 ± 1371.2* | 69164.0 ± 2282.4* | 60.5 ± 3.0 | 14.6 ± 0.6 | 4056.7 ± 77.1 | 2899.7 ± 95.7 | 6465.3 ± 148.7 | 5071.5 ± 177.5 | 918.9 ± 26.6 | 1181.8 ± 39.0 | 116.3 ± 2.7 | 94.4 ± 1.4 |
| **Nov. 29** | 67706.9 ± 2843.7* | 69502.0 ± 2988.6* | 23.2 ± 1.0 | 8.2 ± 0.4 | 3907.3 ± 164.1 | 2959.6 ± 127.3 | 6286.0 ± 276.6 | 6298.7 ± 69.3* | 910.5 ± 15.5 | 1408.8 ± 49.3* | 115.0 ± 5.1 | 132.7 ± 1.5* |
| **Dec. 13** | 66027.7 ± 2112.9* | 67314.7 ± 1952.1 | 20.1 ± 0.6 | 3.0 ± 0.1 | 3969.1 ± 127.0 | 2868.2 ± 83.2 | 6673.4 ± 233.6 | 6048.8 ± 163.3* | 992.4 ± 11.9* | 1394.5 ± 54.4* | 117.2 ± 1.8 | 137.0 ± 3.7* |
| **Dec. 28** | 69436.7 ± 2013.7* | 68303.3 ± 2254.0 | 20.7 ± 0.6 | 3.2 ± 0.1 | 3636.1 ± 105.4 | 2886.1 ± 95.2 | 6713.8 ± 235.0 | 6160.4 ± 184.8* | 961.1 ± 27.9* | 1385.7 ± 47.1* | 122.5 ± 3.1 | 135.9 ± 4.1* |

Results are presented as means ± SD of the samples collected at different dates in a certain forest plot. Values marked with * in the same column indicate the highest ($p < 0.05$) amounts in samples.

**Table S2.** Contents of catechins and B-type proanthocyanidins (μg/g DW ± SD) in young and old lingonberry leaves, collected throughout one year.

| Date | (+)-Catechin | | (−)-Epicatechin | | Procyanidin B1 | | Procyanidin B2 | | Procyanidin B3 | | Procyanidin C1 | |
|---|---|---|---|---|---|---|---|---|---|---|---|---|
| | Young leaves | Old leaves | Young leaves | Old leaves | Young leaves | Old leaves | Young leaves | Old leaves | Young leaves | Old leaves | Young leaves | Old leaves |
| **Jan. 11** | 16561.6 ± 281.6* | 14760.7 ± 560.9* | 2616.7 ± 44.5 | 2938.0 ± 111.5 | 6336.9 ± 190.1* | 5351.9 ± 133.8 | 2595.4 ± 44.1* | 3532.9 ± 63.6 | 9594.8 ± 163.1* | 7680.5 ± 291.9 | 4782.1 ± 81.3* | 4584.4 ± 128.4 |
| **Jan. 24** | 15309.4 ± 643.0 | 12113.6 ± 436.1 | 2442.3 ± 102.6 | 1813.6 ± 65.3 | 5462.5 ± 125.6 | 4258.1 ± 149.0 | 2290.8 ± 96.2 | 2828.6 ± 101.8 | 7926.0 ± 332.9 | 6194.1 ± 223.0 | 4050.4 ± 170.1 | 3997.6 ±143.9 |
| **Feb. 7** | 15108.0 ± 483.5 | 13672.3 ± 533.2 | 2167.0 ± 69.3 | 1823.6 ± 71.1 | 5374.7 ± 236.5 | 3964.6 ± 43.6 | 2169.6 ± 69.4 | 2757.8 ± 80.0 | 8018.2 ± 256.6 | 6456.5 ± 187.2 | 4077.1 ± 130.5 | 3836.6 ± 149.6 |
| **Feb. 21** | 15009.6 ± 435.3 | 13210.1 ± 435.9 | 2097.4 ± 60.8 | 1959.1 ± 64.7 | 5358.7 ± 241.1 | 3981.7 ± 135.4 | 2201.8 ± 63.9 | 2824.2 ± 93.2 | 7715.7 ± 146.6 | 6436.4 ± 212.4 | 4201.1 ± 121.8 | 3778.2 ± 124.7 |
| **Mar. 7** | 15105.9 ± 558.9 | 10307.0 ± 185.5 | 2097.6 ± 77.6 | 1990.6 ± 35.8 | 5045.5 ± 176.6 | 4028.2 ± 88.6 | 2200.9 ± 81.4 | 2769.1 ± 49.8 | 6386.7 ± 236.3 | 4753.4 ± 85.6 | 4022.8 ± 148.8 | 3750.7 ± 67.5 |
| **Mar. 21** | 15097.3 ± 452.9 | 10198.3 ± 255.0 | 2172.3 ± 65.2 | 2020.4 ± 50.5 | 5080.7 ± 182.9 | 4693.1 ± 211.2 | 2138.9 ± 64.2 | 2813.3 ± 70.3 | 6257.1 ± 187.7 | 4819.7 ± 120.5 | 3920.0 ± 117.6 | 3472.1 ± 121.5 |
| **Apr. 4** | 13346.1 ± 440.4 | 10480.2 ± 199.1 | 2167.8 ± 65.0 | 2085.0 ± 52.1 | 4974.1 ± 109.4 | 4749.5 ± 180.5 | 2164.2 ± 64.9 | 2753.9 ± 68.8 | 6486.3 ± 214.0 | 5462.4 ± 103.8 | 3954.4 ± 87.0 | 3674.3 ± 95.5 |
| **Apr. 17** | 13330.1 ± 439.9 | 12884.8 ± 541.2 | 1707.4 ± 51.2 | 2208.2 ± 37.5 | 4755.8 ± 199.7 | 4614.4 ± 166.1 | 2079.4 ± 62.4 | 3202.8 ± 54.4 | 5693.5 ± 187.9 | 6197.6 ± 260.3 | 3790.8 ± 113.7 | 4010.1 ± 68.2 |
| **May 1** | 11273.5 ± 484.8 | 11386.4 ± 250.5 | 796.7 ± 19.9 | 2277.3 ± 68.3 | 2044.2 ± 65.4 | 4532.6 ± 86.1 | 1047.3 ± 26.2 | 3139.1 ± 94.2 | 2919.6 ± 125.5 | 5754.7 ± 126.6 | 1477.8 ± 37.0 | 2897.8 ± 86.9 |
| **May 15** | 11280.3 ± 248.2 | 11356.0 ± 329.3 | 1062.5 ± 20.2 | 2303.7 ± 69.1 | 1728.8 ± 50.1 | 4815.4 ± 158.9 | 864.3 ± 16.4 | 3066.7 ± 92.0 | 2915.5 ± 64.1 | 5664.8 ± 220.9 | 1934.1 ± 36.8 | 3195.0 ± 47.9 |
| **May 29** | 4043.9 ± 121.3 | 7118.8 ± 163.7 | 1086.1 ± 27.2 | 2313.9 ± 53.2 | 1889.0 ± 69.9 | 4712.8 ± 160.2 | 900.6 ± 22.5 | 3074.9 ± 70.7 | 3065.2 ± 92.0 | 5787.1 ± 133.1 | 2162.8 ± 75.7 | 3415.5 ± 95.6 |
| **Jun. 13** | 2329.2 ± 72.2 | 8407.1 ± 378.3 | 1062.1 ± 32.9 | 2339.4 ± 110.0 | 972.7 ± 15.6 | 4816.5 ± 53.0 | 659.7 ± 7.3 | 3060.8 ± 143.9 | 1591.9 ± 49.3 | 5676.1 ± 255.4 | 2634.5 ± 39.5 | 3525.6 ± 102.2 |
| **Jun. 28** | 2094.5 ± 71.2 | 8347.9 ± 308.9 | 1010.8 ± 34.4 | 2288.0 ± 84.7 | 998.3 ± 15.0 | 4683.5 ± 145.2 | 765.3 ± 26.0 | 3126.3 ± 53.1 | 1055.6 ± 35.9 | 5496.5 ± 148.4 | 2635.6 ± 29.0 | 3771.9 ± 94.3 |
| **Jul. 15** | 3072.1 ± 86.0 | 7213.5 ± 180.3 | 1051.3 ± 29.4 | 2161.7 ± 54.0 | 1492.3 ± 32.8 | 4817.5 ± 144.5 | 1116.4 ± 31.3 | 3168.3 ± 79.2 | 1276.4 ± 35.7 | 5779.7 ± 86.7 | 2787.4 ± 64.1 | 3867.1 ± 120.0 |
| **Jul. 29** | 2952.0 ± 47.2 | 6926.5 ± 187.0 | 1060.6 ± 30.7 | 2135.2 ± 64.1 | 1497.9 ± 67.4 | 4607.9 ± 101.4 | 1125.3 ± 32.6 | 2970.3 ± 89.1 | 1407.1 ± 22.5 | 5874.6 ± 158.6 | 3012.7 ± 141.6 | 3943.5 ± 134.1 |
| **Aug. 11** | 3238.9 ± 100.4 | 6889.2 ± 248.0 | 1053.7 ± 26.3 | 2140.3 ± 49.2 | 3190.7 ± 121.2 | 4560.0 ± 191.5 | 1393.3 ± 34.8 | 3015.8 ± 69.4 | 2766.3 ± 85.8 | 5917.9 ± 213.0 | 3210.8 ± 54.6 | 4125.4 ± 94.9 |
| **Aug. 24** | 4521.5 ± 67.8 | 9091.1 ± 327.3 | 1205.9 ± 18.1 | 2190.3 ± 78.9 | 3810.7 ± 99.1 | 4479.9 ± 143.4 | 1384.6 ± 20.8 | 3074.8 ± 49.2 | 4764.9 ± 71.5 | 6135.4 ± 220.9 | 3672.6 ± 55.1 | 3953.7 ± 142.3 |
| **Sep. 6** | 8525.8 ± 298.4 | 11284.9 ± 338.5 | 1271.5 ± 44.5 | 2275.1 ± 68.3 | 4196.5 ± 79.7 | 4370.8 ± 126.8 | 1459.3 ± 51.1 | 3352.2 ± 70.4 | 5171.7 ± 181.0 | 6489.6 ± 194.7 | 4070.7 ± 142.5 | 4321.3 ± 129.6 |
| **Sep. 20** | 13126.1 ± 367.5 | 11414.7 ± 479.4 | 1773.5 ± 51.4 | 2375.0 ± 99.8 | 5056.2 ± 116.3 | 4450.3 ± 164.7 | 1708.7 ± 49.6 | 3311.1 ± 139.1 | 6758.1 ± 121.6 | 6518.9 ± 273.8 | 4505.9 ± 130.7 | 4293.4 ± 128.8 |
| **Oct. 4** | 13877.8 ± 222.0 | 14155.1 ± 523.7 | 1755.7 ± 28.1 | 2747.1 ± 101.6 | 5301.2 ± 95.4 | 6260.1 ± 156.5 | 1671.4 ± 26.7 | 3605.0 ± 133.4 | 6763.8 ± 108.2 | 6606.3 ± 244.4 | 4492.3 ± 71.9 | 4478.1 ± 120.9 |
| **Oct. 19** | 15313.4 ± 520.7 | 12931.9 ± 465.6 | 2495.2 ± 84.8 | 2703.1 ± 97.3 | 5289.0 ± 158.7 | 4345.7 ± 117.3 | 2264.0 ± 77.0 | 3545.4 ± 127.6 | 7901.5 ± 268.7 | 6649.6 ± 239.4 | 4646.9 ± 58.0 | 4433.6 ± 159.6 |
| **Nov. 1** | 15938.9 ± 239.1 | 13411.6 ± 442.6 | 2578.5 ± 38.7 | 2757.7 ± 91.0 | 5404.1 ± 135.1 | 5285.0 ± 158.6 | 2317.9 ± 34.8 | 3506.0 ± 115.7 | 8522.8 ± 127.8 | 7109.4 ± 234.6 | 4573.1 ± 68.6 | 4539.1 ± 149.8 |
| **Nov. 16** | 16154.6 ± 468.5* | 13917.0 ± 459.3 | 2870.0 ± 83.2 | 2778.3 ± 91.7 | 6140.7 ± 135.1 | 5351.3 ± 32.1 | 2599.3 ± 75.4* | 3737.8 ± 123.3* | 8632.0 ± 250.3 | 7314.8 ± 241.4 | 4713.5 ± 96.7* | 4800.3 ± 158.4 |
| **Nov. 29** | 16110.9 ± 676.7* | 15211.5 ± 654.1* | 2931.3 ± 99.1* | 3441.6 ± 148.0* | 5557.6 ± 122.3 | 6733.8 ± 195.3* | 2634.9 ± 110.7* | 3998.4 ± 171.9* | 8394.2 ± 352.6 | 8357.4 ± 359.4* | 4793.9 ± 201.3* | 5016.6 ± 215.7* |
| **Dec. 13** | 16211.4 ± 518.8* | 13869.0 ± 540.9 | 3014.1 ± 96.5* | 3029.6 ± 118.2 | 6067.9 ± 182.0 | 6259.7 ± 156.5 | 2642.3 ± 84.6* | 3801.2 ± 148.2* | 8542.4 ± 273.4 | 8250.6 ± 321.8* | 4960.2 ± 158.7* | 5004.7 ± 195.2* |
| **Dec. 28** | 16248.8 ± 471.2* | 14483.2 ± 477.9* | 2759.5 ± 80.0 | 3041.7 ± 130.8 | 6435.9 ± 135.2* | 5499.2 ± 104.5 | 2646.7 ± 76.8* | 3805.1 ± 163.6* | 9112.6 ± 264.3 | 8059.2 ± 266.0 | 4886.4 ± 117.3* | 4804.1 ± 62.5 |

Results are presented as means ± SD of the samples collected at different dates in a certain forest plot. Values marked with * in the same column indicate the highest ($p < 0.05$) amounts in samples.

**Table S3.** Contents of flavonol aglycones and phenolic acids (μg/g DW ± SD) in young and old lingonberry leaves, collected throughout one year.

| **Date** | **Quercetin** | | **Kaempferol** | | **Chlorogenic acid** | | **Cryptochlorogenic acid** | | **Neochlorogenic acid** | | ***p*-Coumaric acid** | |
|---|---|---|---|---|---|---|---|---|---|---|---|---|
| | **Young leaves** | **Old leaves** | **Young leaves** | **Old leaves** | **Young leaves** | **Old leaves** | **Young leaves** | **Old leaves** | **Young leaves** | **Old leaves** | **Young leaves** | **Old leaves** |
| **Jan. 11** | 68.8 ± 1.9 | 61.9 ± 1.7 | NQ | NQ | 495.5 ± 8.4 | 531.8 ± 9.6 | 1166.5 ± 18.7 | 811.8 ± 12.2 | 59.7 ± 0.7 | 80.9 ± 3.1 | 308.4 ± 5.2 | 171.8 ± 2.1 |
| **Jan. 24** | 71.5 ± 3.0 | 70.4 ± 2.5 | NQ | NQ | 490.5 ± 5.9 | 530.3 ± 13.8 | 1102.8 ± 36.4 | 772.3 ± 22.4 | 61.5 ± 2.6 | 77.6 ± 2.8 | 277.7 ± 12.5 | 177.7 ± 6.4 |
| **Feb. 7** | 70.8 ± 2.3 | 67.1 ± 2.6 | NQ | NQ | 541.5 ± 11.9 | 513.5 ± 14.9 | 1020.4 ± 43.9 | 793.9 ± 23.0 | 71.0 ± 1.6* | 67.9 ± 0.6 | 294.7 ± 12.4 | 179.9 ± 5.2 |
| **Feb. 21** | 71.1 ± 2.8 | 69.7 ± 1.6 | NQ | NQ | 546.6 ± 10.4 | 446.1 ± 5.8 | 999.8 ± 39.0 | 779.2 ± 17.9 | 59.5 ± 1.7 | 62.4 ± 2.1 | 323.9 ± 12.6 | 177.9 ± 4.1 |
| **Mar. 7** | 72.3 ± 2.7 | 67.7 ± 1.2 | NQ | NQ | 463.7 ± 7.9 | 474.4 ± 13.3 | 883.3 ± 15.0 | 678.2 ± 12.2 | 52.0 ± 1.9 | 62.3 ± 0.6 | 325.7 ± 12.1 | 187.3 ± 3.4 |
| **Mar. 21** | 67.3 ± 2.0 | 67.3 ± 1.7 | NQ | NQ | 682.5 ± 14.3 | 448.2 ± 15.7 | 896.9 ± 26.9 | 677.0 ± 16.9 | 48.6 ± 1.5 | 68.7 ± 1.7 | 332.2 ± 11.3 | 165.7 ± 2.5 |
| **Apr. 4** | 68.3 ± 2.3 | 66.7 ± 1.3 | NQ | NQ | 393.1 ± 5.1 | 424.7 ± 8.1 | 531.7 ± 11.7 | 651.6 ± 16.3 | 48.8 ± 1.5 | 73.0 ± 2.0 | 277.3 ± 11.6 | 156.3 ± 3.9 |
| **Apr. 17** | 66.6 ± 2.2 | 67.9 ± 2.8 | NQ | NQ | 234.1 ± 5.4 | 419.4 ± 13.4 | 545.6 ± 12.0 | 661.8 ± 11.3 | 35.1 ± 0.9 | 74.5 ± 2.2 | 149.9 ± 4.5 | 122.6 ± 2.1 |
| **May 1** | 59.1 ± 0.8 | 65.8 ± 2.1 | 3.5 ± 0.1 | NQ | 217.1 ± 5.0 | 394.4 ± 16.6 | 433.8 ± 11.3 | 628.1 ± 15.7 | 35.6 ± 0.8 | 76.1 ± 1.2 | 107.2 ± 1.6 | 147.3 ± 6.0 |
| **May 15** | 67.3 ± 1.5 | 68.5 ± 1.3 | 3.6 ± 0.1 | NQ | 355.1 ± 7.8 | 391.6 ± 15.3 | 791.1 ± 14.2 | 632.0 ± 9.5 | 42.1 ± 0.9 | 80.6 ± 2.3 | 203.1 ± 3.7 | 190.7 ± 2.9 |
| **May 29** | 72.2 ± 2.2 | 69.9 ± 1.6 | 4.0 ± 0.1 | 3.7 ± 0.1 | 416.2 ± 12.5 | 399.8 ± 9.2 | 872.1 ± 34.0 | 613.6 ± 18.4 | 45.0 ± 0.9 | 80.6 ± 1.9 | 303.1 ± 11.5 | 182.9 ± 6.4 |
| **Jun. 13** | 100.2 ± 4.1 | 80.4 ± 2.0 | 10.3 ± 0.2* | 4.2 ± 0.1* | 765.5 ± 23.7 | 460.3 ± 11.5 | 1143.6 ± 40.0 | 697.3 ± 23.0 | 44.7 ± 1.4 | 80.3 ± 3.6 | 701.7 ± 17.4* | 215.6 ± 4.5 |
| **Jun. 28** | 161.6 ± 7.1* | 82.5 ± 2.2 | 13.1 ± 0.4* | 4.3 ± 0.1* | 714.7 ± 30.0 | 544.9 14.7 | 1131.8 ± 39.6 | 694.1 ± 25.0 | 46.8 ± 1.6 | 79.2 ± 2.7 | 614.3 ± 9.2 | 229.3 ± 3.0 |
| **Jul. 15** | 130.5 ± 3.7 | 94.7 ± 1.4* | 4.6 ± 0.2 | 4.4 ± 0.1* | 797.9 ± 30.3* | 478.7 ± 7.2 | 1277.7 ± 17.1* | 713.6 ± 15.7 | 50.2 ± 2.2 | 82.7 ± 3.5 | 579.5 ± 8.7 | 231.0 ± 2.8 |
| **Jul. 29** | 132.5 ± 2.1 | 90.3 ± 2.4* | 4.6 ± 0.1 | 4.4 ± 0.1* | 782.2 ± 20.3* | 526.7 ± 9.0 | 1266.3 ± 44.3* | 720.4 ± 16.6 | 50.3 ± 0.7 | 80.3 ± 2.6 | 611.0 ± 21.4 | 239.6 ± 7.2 |
| **Aug. 11** | 126.6 ± 5.2 | 87.8 ± 2.3* | 4.8 ± 0.2 | 4.1 ± 0.1 | 797.1 ± 24.7* | 597.6 ± 15.5 | 1275.6 ± 39.5* | 767.7 ± 20.7 | 55.6 ± 2.2 | 78.1 ± 2.8 | 599.6 ± 10.2 | 238.1 ± 3.6 |
| **Aug. 24** | 117.8 ± 4.1 | 88.9 ± 2.3* | 4.8 ± 0.2 | 4.1 ± 0.1 | 809.7 ± 36.4* | 579.7 ± 15.1 | 1338.9 ± 45.5* | 835.9 ± 30.9 | 51.7 ± 1.3 | 73.1 ± 2.4 | 651.1 ± 22.7 | 220.9 ± 5.7 |
| **Sep. 6** | 107.4 ± 3.8 | 92.6 ± 2.8* | 4.7 ± 0.2 | 4.0 ± 0.1 | 814.7 ± 28.5* | 599.7 ± 12.6 | 1329.4 ± 46.5* | 871.9 ± 18.3 | 55.7 ± 1.4 | 75.7 ± 2.3 | 533.1 ± 20.3 | 257.9 ± 3.1 |
| **Sep. 20** | 93.4 ± 3.5 | 79.7 ± 2.5 | 4.2 ± 0.1 | 4.1 ± 0.1 | 787.4 ± 14.2* | 584.6 ± 24.6 | 1330.0 ± 38.6* | 893.4 ± 28.6 | 57.1 ± 1.6 | 79.3 ± 3.3 | 499.5 ± 14.5 | 239.2 ± 5.3 |
| **Oct. 4** | 83.6 ± 1.3 | 73.5 ± 2.7 | 3.9 ± 0.1 | 4.2 ± 0.2* | 733.1 ± 19.1 | 670.7 ± 18.1* | 1308.1 ± 20.9* | 936.0 ± 16.8* | 55.6 ± 0.9 | 80.4 ± 2.2 | 430.2 ± 5.6 | 274.8 ± 3.3 |
| **Oct. 19** | 82.7 ± 2.8 | 69.6 ± 2.5 | 3.9 ± 0.1 | NQ | 756.2 ± 25.7 | 667.3 ± 17.4* | 1291.1 ± 54.2* | 837.0 ± 19.3 | 56.9 ± 1.8 | 80.4 ± 2.1 | 443.6 ± 14.6 | 249.1 ± 3.0 |
| **Nov. 1** | 82.7 ± 1.2 | 71.0 ± 2.3 | 3.5 ± 0.1 | 3.6 ± 0.1 | 764.3 ± 11.5 | 634.7 ± 8.3 | 1309.6 ± 41.9* | 808.3 ± 15.4 | 57.7 ± 0.9 | 93.1 ± 3.1* | 413.2 ± 12.0 | 302.3 ± 7.3 |
| **Nov. 16** | 83.0 ± 2.4 | 72.2 ± 2.4 | 3.5 ± 0.1 | NQ | 768.7 ± 24.6 | 625.2 ± 14.4 | 1280.2 ± 37.1* | 753.4 ± 24.9 | 56.6 ± 1.1 | 79.1 ± 2.6 | 387.2 ± 8.9 | 376.3 ± 6.4* |
| **Nov. 29** | 76.9 ± 3.2 | 69.1 ± 3.0 | NQ | NQ | 754.3 ± 31.7 | 673.0 ± 15.5* | 1118.7 ± 19.0 | 982.0 ± 14.7* | 59.8 ± 2.5 | 81.6 ± 3.5 | 378.1 ± 6.8 | 398.8 ± 9.2* |
| **Dec. 13** | 76.3 ± 2.4 | 66.1 ± 1.3 | NQ | NQ | 767.4 ± 26.1 | 650.1 ± 12.4* | 1217.5 ± 51.1 | 963.6 ± 18.3* | 65.7 ± 2.8 | 80.5 ± 2.3 | 363.5 ± 8.0 | 283.2 ± 5.4 |
| **Dec. 28** | 72.2 ± 2.1 | 64.6 ± 2.1 | NQ | NQ | 518.2 ± 15.0 | 551.4 ± 12.7 | 1172.4 ± 57.4 | 889.5 ± 21.3 | 62.3 ± 1.8 | 79.5 ± 2.6 | 362.4 ± 10.5 | 264.5 ± 6.1 |

Results are presented as means ± SD of the samples collected at different dates in a certain forest plot. Values marked with * in the same column indicate the highest ($p < 0.05$) amounts in samples. ND—not detected, NQ—not quantified (amount below the limits of quantification (LOQ)).

**Table S4.** Contents of kaempferol and quercetin glycosides (μg/g DW ± SD) in young and old lingonberry leaves, collected throughout one year.

| Date | Nicotiflorin | | Astragalin | | Afzelin | | Quercitrin | | Quercetin-HMG-rhamnoside | | 6”-*O*-acetylisoquercitrin | |
|---|---|---|---|---|---|---|---|---|---|---|---|---|
| | Young leaves | Old leaves | Young leaves | Old leaves | Young leaves | Old leaves | Young leaves | Old leaves | Young leaves | Old leaves | Young leaves | Old leaves |
| **Jan. 11** | 74.0 ± 1.2 | 54.8 ± 1.4 | 65.3 ± 1.0 | 51.1 ± 1.3 | 73.4 ± 1.2 | 73.8 ± 3.1 | 1110.9 ± 52.2 | 1338.6 ± 37.5 | 1407.4 ± 38.0 | 1278.0 ± 23.0 | 75.4 ± 1.2 | 60.9 ± 0.9 |
| **Jan. 24** | 66.4 ± 2.2 | 55.1 ± 1.6 | 63.9 ± 2.1 | 55.3 ± 1.6 | 73.1 ± 3.3 | 68.4 ± 2.5 | 1161.2 ± 48.8 | 906.6 ± 32.6 | 1061.0 ± 12.7 | 1178.5 ± 30.6 | 77.2 ± 2.5 | 60.0 ± 1.7 |
| **Feb. 7** | 68.5 ± 2.3 | 65.5 ± 1.9 | 69.0 ± 3.0 | 60.3 ± 1.7 | 73.2 ± 3.1 | 68.5 ± 2.0 | 1043.2 ± 33.4 | 1494.8 ± 23.3* | 1077.1 ± 23.7 | 1252.5 ± 36.3 | 77.3 ± 3.3 | 56.7 ± 1.6 |
| **Feb. 21** | 85.9 ± 3.3 | 61.6 ± 1.4 | 67.8 ± 2.6 | 62.6 ± 1.4 | 77.9 ± 3.0 | 66.9 ± 1.5 | 1117.3 ± 32.4 | 1146.0 ± 37.8 | 1126.1 ± 21.4 | 1284.8 ± 42.4 | 77.6 ±3.0 | 57.4 ± 1.3 |
| **Mar. 7** | 83.2 ± 1.4 | 64.3 ± 1.2 | 72.4 ± 1.2 | 65.4 ± 1.2 | 88.8 ± 3.3 | 63.4 ± 1.1 | 1081.4 ± 40.0 | 969.5 ± 17.5 | 1179.7 ± 20.1 | 1200.3 ± 33.6 | 73.9 ± 1.3 | 42.0 ± 0.8 |
| **Mar. 21** | 102.3 ± 3.1 | 65.1 ± 1.6 | 94.2 ± 2.8 | 66.7 ± 1.7 | 94.8 ± 3.2* | 60.6 ± 0.9 | 1216.5 ± 36.5 | 982.7 ± 24.6 | 1042.9 ± 21.9 | 1169.1 ± 40.9 | 82.0 ± 1.5* | 39.9 ± 1.0 |
| **Apr. 4** | 103.4 ± 3.1 | 65.0 ± 1.6 | 101.9 ± 3.1 | 66.0 ± 1.6 | 84.5 ± 3.5 | 56.1 ± 1.4 | 972.6 ± 21.4 | 946.4 ± 24.6 | 1097.9 ± 14.3 | 1112.7 ± 21.1 | 65.6 ± 2.0 | 39.5 ± 1.0 |
| **Apr. 17** | 105.1 ± 4.4 | 74.5 ± 2.0 | 87.7 ± 3.7 | 71.7 ± 1.2 | 79.1 ± 2.4 | 55.5 ± 0.9 | 966.9 ± 29.0 | 1408.6 ± 23.9 | 1126.6 ± 37.2 | 1059.6 ± 23.3 | 66.6 ± 2.8 | 44.5 ± 0.8 |
| **May 1** | 88.2 ± 3.1 | 67.4 ± 2.0 | 88.5 ± 2.3 | 67.9 ± 1.7 | 32.5 ± 0.5 | 44.4 ± 1.8 | 354.0 ± 8.8 | 1025.9 ± 30.8 | 1081.2 ± 24.9 | 1193.7 ± 26.3 | 24.0 ± 0.6 | 46.4 ± 1.2 |
| **May 15** | 94.8 ± 2.8 | 74.9 ± 2.2 | 89.7 ± 1.6 | 76.5 ± 1.1 | 24.9 ± 0.4 | 46.2 ± 1.2 | 348.2 ± 6.6 | 1115.0 ± 16.7 | 1064.3 ± 23.4 | 1114.5 ± 43.5 | 25.8 ± 0.5 | 49.2 ± 0.7 |
| **May 29** | 99.9 ± 3.5 | 74.1 ± 1.7 | 95.8 ± 3.7 | 69.7 ± 2.1 | 25.7 ± 0.7 | 45.0 ± 1.6 | 404.8 ± 14.2 | 819.0 ± 22.9 | 1372.3 ± 41.2 | 948.0 ± 21.8 | 26.2 ± 1.0 | 45.5 ± 1.4 |
| **Jun. 13** | 240.8 ± 9.9* | 82.0 ± 2.2 | 212.3 ± 9.6* | 70.0 ± 1.6 | 24.7 ± 1.0 | 52.4 ± 1.1 | 473.1 ± 7.1 | 1235.4 ± 35.8 | 2415.9 ± 74.9 | 983.9 ± 24.6 | 28.4 ± 1.0 | 48.7 ± 1.6 |
| **Jun. 28** | 235.7 ± 8.0* | 77.9 ± 1.3 | 161.1 ± 7.2 | 66.1 ± 1.1 | 24.3 ± 0.4 | 59.1 ± 2.0 | 513.9 ± 5.7 | 1226.6 ± 30.7 | 2644.7 ± 111.1* | 1382.6 ± 37.3 | 25.6 ± 0.4 | 56.7 ± 2.0 |
| **Jul. 15** | 184.2 ± 3.3 | 81.1 ± 1.2 | 112.7 ± 3.3 | 65.4 ± 2.0 | 18.3 ± 0.3 | 59.7 ± 2.5 | 548.3 ± 12.6 | 1124.8 ± 34.9 | 2464.5 ± 93.7 | 1435.3 ± 21.5 | 30.0 ± 0.9 | 55.7 ± 2.3 |
| **Jul. 29** | 127.2 ± 5.0 | 78.0 ± 2.3 | 107.6 ± 3.8 | 71.6 ± 1.6 | 21.5 ± 0.5 | 57.0 ± 1.7 | 511.5 ± 18.9 | 1155.1 ± 39.3 | 2101.6 ± 54.6 | 1405.8 ± 23.9 | 29.2 ± 1.0 | 54.2 ± 1.2 |
| **Aug. 11** | 139.2 ± 3.5 | 84.4 ± 1.9 | 118.6 ± 4.9 | 63.0 ± 1.7 | 38.3 ± 0.7 | 59.0 ± 0.9 | 649.1 ± 11.0 | 1021.7 ± 23.5 | 2067.9 ± 64.1 | 1406.7 ± 36.6 | 43.9 ± 1.8 | 52.9 ± 1.4 |
| **Aug. 24** | 150.3 ± 6.8 | 83.2 ± 1.3 | 121.3 ± 4.1 | 65.0 ± 1.1 | 59.9 ± 1.3 | 66.3 ± 2.4 | 695.1 ± 10.4 | 927.0 ± 33.4 | 1741.5 ± 78.4 | 1477.1 ± 38.4 | 52.4 ± 1.8 | 59.1 ± 2.2 |
| **Sep. 6** | 125.1 ± 4.4 | 95.7 ± 2.0* | 95.9 ± 3.4 | 88.1 ± 1.9 | 56.4 ± 2.1 | 62.7 ± 2.6 | 711.0 ± 24.9 | 1187.7 ± 35.6 | 1437.9 ± 50.3 | 1578.5 ± 33.1 | 51.6 ± 1.8 | 59.2 ± 1.2 |
| **Sep. 20** | 116.4 ± 3.4 | 95.3 ± 3.1* | 101.5 ± 2.9 | 104.9 ± 3.4* | 66.2 ± 1.9 | 62.6 ± 2.6 | 708.6 ± 20.5 | 966.6 ± 29.0 | 1436.9 ± 25.9 | 1586.6 ± 66.6 | 53.6 ± 1.6 | 60.4 ± 1.9 |
| **Oct. 4** | 94.9 ± 1.5 | 60.8 ± 1.1 | 90.0 ± 1.4 | 105.7 ± 1.9* | 60.8 ± 0.8 | 65.1 ± 2.7 | 690.0 ± 11.0 | 1231.9 ± 33.3 | 1410.1 ± 36.7 | 1409.1 ± 38.0 | 54.4 ± 0.9 | 58.4 ± 1.1 |
| **Oct. 19** | 88.5 ± 3.7 | 50.9 ± 2.2 | 86.2 ± 3.6 | 95.4 ± 4.1 | 69.7 ± 2.3 | 67.7 ± 0.8 | 962.3 ± 32.7 | 984.2 ± 35.4 | 1483.1 ± 50.4 | 1414.8 ± 36.8 | 67.7 ± 2.8 | 63.1 ± 2.7 |
| **Nov. 1** | 97.9 ± 3.1 | 48.0 ± 0.9 | 100.3 ± 3.2 | 91.8 ± 1.7 | 76.8 ± 2.2 | 67.8 ± 2.0 | 1037.7 ± 15.6 | 1106.3 ± 36.5 | 1635.4 ± 24.5 | 1383.1 ± 18.0 | 69.9 ± 2.2 | 65.6 ± 1.2 |
| **Nov. 16** | 93.0 ± 2.7 | 51.7 ± 1.7 | 97.9 ± 2.8 | 67.7 ± 2.2 | 77.6 ± 1.8 | 68.4 ± 1.2 | 1072.9 ± 31.1 | 968.8 ± 32.0 | 1890.9 ± 60.5 | 1519.1 ± 34.9 | 68.5 ± 2.0 | 63.0 ± 2.1 |
| **Nov. 29** | 91.2 ± 1.6 | 50.7 ± 1.8 | 83.8 ± 1.4 | 70.8 ± 2.5 | 73.5 ± 1.3 | 84.1 ± 1.9* | 1057.2 ± 44.4 | 1305.9 ± 56.2 | 1634.2 ± 68.6 | 1735.1 ± 39.9* | 69.8 ± 1.2 | 71.2 ±2.5* |
| **Dec. 13** | 79.8 ± 3.4 | 45.0 ± 0.9 | 78.1 ± 3.3 | 50.4 ± 1.0 | 77.2 ± 1.7 | 73.8 ± 2.9 | 1367.1 ± 43.7* | 1114.2 ± 21.2 | 1538.1 ± 52.3 | 1390.3 ± 26.4 | 74.0 ± 3.1 | 62.0 ± 1.2 |
| **Dec. 28** | 62.8 ± 3.1 | 50.5 ± 1.2 | 64.0 ± 3.1 | 51.3 ± 1.2 | 73.7 ± 2.1 | 76.7 ± 1.8 | 1310.6 ± 57.7* | 1220.8 ± 15.9 | 1465.6 ± 42.5 | 1380.7 ± 45.6 | 74.9 ± 3.7 | 62.0 ± 1.5 |

Results are presented as means ± SD of the samples collected at different dates in a certain forest plot. Values marked with * in the same column indicate the highest ($p < 0.05$) amounts in samples.

**Table S5.** Contents of quercetin glycosides (μg/g DW ± SD) in young and old lingonberry leaves, collected throughout one year.

| Date | Rutin | | Hyperoside | | Isoquercitrin | | Reynoutrin | | Guaiaverin | | Avicularin | |
|---|---|---|---|---|---|---|---|---|---|---|---|---|
| | Young leaves | Old leaves | Young leaves | Old leaves | Young leaves | Old leaves | Young leaves | Old leaves | Young leaves | Old leaves | Young leaves | Old leaves |
| **Jan. 11** | 727.4 ± 12.4 | 444.0 ± 12.4 | 2573.7 ± 43.8 | 1906.1 ± 34.3 | 751.3 ± 39.1 | 640.6 ± 6.4 | 592.5 ± 10.1 | 507.5 ± 9.1 | 1062.4 ± 18.1 | 911.4 ± 16.4 | 3379.9 ± 98.0 | 2286.0 ± 75.4 |
| **Jan. 24** | 806.0 ± 33.9 | 676.5 ± 24.4 | 2625.6 ± 110.3 | 2019.2 ± 72.7 | 801.2 ± 17.6 | 680.7 ± 19.7 | 567.4 ± 23.8 | 546.7 ± 19.7 | 1146.9 ± 48.2 | 925.2 ± 24.1 | 3126.0 ± 146.9 | 2159.2 ± 54.0 |
| **Feb. 7** | 853.9 ± 27.3 | 692.2 ± 27.0 | 2734.7 ± 114.9 | 1926.9 ± 55.9 | 712.3 ± 21.4 | 888.4 ± 9.8* | 626.5 ± 26.3 | 581.8 ± 16.9 | 1177.7 ± 49.5 | 849.1 ± 24.6 | 3500.9 ± 115.5 | 2182.6 ± 63.3 |
| **Feb. 21** | 907.1 ± 26.3 | 715.1 ± 23.6 | 2876.8 ± 83.4 | 1780.9 ± 58.8 | 727.5 ± 32.7 | 645.2 ± 15.5 | 680.0 ± 19.7 | 528.7 ± 17.4 | 1431.2 ± 41.5 | 872.5 ± 20.1 | 3783.5 ± 87.6* | 2153.4 ± 49.5 |
| **Mar. 7** | 947.1 ± 35.0 | 670.1 ± 12.1 | 3240.0 ± 119.9* | 1703.0 ± 47.7 | 747.2 ± 26.2 | 631.2 ± 7.6 | 671.3 ± 24.8 | 441.1 ± 7.9 | 1568.9 ± 73.7 | 783.1 ± 21.9 | 2881.9 ± 49.0 | 1842.8 ± 33.2 |
| **Mar. 21** | 1220.3 ± 36.6 | 696.8 ± 17.4 | 3754.9 ± 127.7* | 1794.8 ± 44.9 | 819.0 ± 37.7 | 642.3 ± 28.9 | 841.3 ± 28.6* | 412.7 ± 6.2 | 1770.2 ± 77.9* | 829.0 ± 12.4 | 2914.4 ± 122.4 | 1945.1 ± 48.6 |
| **Apr. 4** | 1202.9 ± 36.1 | 667.1 ± 16.7 | 2722.9 ± 81.7 | 1529.9 ± 38.2 | 739.6 ± 23.7 | 622.5 ± 17.4 | 628.1 ± 26.4 | 398.8 ± 10.0 | 1305.1 ± 39.2 | 710.2 ± 17.8 | 2911.7 ± 87.3 | 1698.2 ± 25.5 |
| **Apr. 17** | 1095.2 ± 32.9 | 751.4 ± 12.8 | 2262.4 ± 67.9 | 1489.9 ± 25.3 | 813.4 ± 34.2 | 686.6 ± 24.7 | 551.2 ± 16.5 | 413.1 ± 7.0 | 1101.8 ± 45.2 | 817.5 ± 13.9 | 2558.0 ± 107.4 | 2039.2 ± 55.1 |
| **May 1** | 1054.4 ± 26.4 | 681.4 ± 20.4 | 867.5 ± 21.7 | 1514.3 ± 45.4 | 303.2 ± 3.6 | 516.0 ± 20.1 | 223.2 ± 3.3 | 408.0 ± 16.7 | 432.8 ± 6.5 | 775.7 ± 32.6 | 992.3 ± 15.9 | 2050.7 ± 71.8 |
| **May 15** | 1186.1 ± 22.5 | 670.6 ± 7.4 | 823.1 ± 15.6 | 1967.2 ± 21.6 | 763.5 ± 14.5 | 596.4 ± 19.7 | 267.9 ± 5.1 | 438.7 ± 9.2 | 441.8 ± 8.4 | 897.7 ± 27.8 | 1065.5 ± 19.2 | 2072.0 ± 31.1 |
| **May 29** | 1314.1 ± 32.9 | 613.0 ± 14.1 | 974.3 ± 24.4 | 1986.9 ± 49.7 | 819.6 ± 27.0 | 546.4 ± 9.3 | 245.0 ± 6.1 | 437.7 ± 15.3 | 488.7 ± 12.2 | 822.6 ± 20.6 | 1130.6 ± 44.1 | 2042.3 ± 61.3 |
| **Jun. 13** | 2312.4 ± 71.7* | 726.5 ± 34.1 | 1052.3 ± 31.6 | 1870.1 ± 35.5 | 1436.5 ± 44.5* | 547.3 ± 8.8 | 221.6 ± 6.6 | 480.8 ± 9.1 | 455.5 ± 13.7 | 856.1 ± 24.8 | 1098.5 ± 27.5 | 2073.1 ± 47.7 |
| **Jun. 28** | 2218.8 ± 119.8* | 723.6 ± 26.8 | 1051.2 ± 11.6 | 1963.2 ± 49.1 | 1204.3 ± 20.5 | 550.6 ± 8.3 | 224.7 ± 2.5 | 485.9 ± 17.0 | 413.8 ± 4.6 | 918.0 ± 32.1 | 1161.5 ± 52.3 | 2120.2 ±33.9 |
| **Jul. 15** | 1518.2 42.5 | 726.0 ± 25.4 | 1039.7 ± 15.6 | 1880.3 ± 67.7 | 1068.3 ± 36.3 | 682.1 ± 13.0 | 272.4 ± 7.9 | 485.8 ± 7.3 | 435.5 ± 6.5 | 876.7 ± 31.6 | 1101.0 ± 31.9 | 2017.1 ± 86.7 |
| **Jul. 29** | 1524.4 ± 59.5 | 704.1 ± 14.8 | 1141.8 ± 43.4 | 2017.1 ± 44.4 | 1108.2 ± 38.8 | 657.8 ± 8.6 | 256.2 ± 10.0 | 463.6 ± 9.7 | 500.2 ± 19.0 | 932.1 ± 20.5 | 1035.7 ± 36.2 | 2114.2 ± 48.6 |
| **Aug. 11** | 1597.9 ± 39.9 | 674.3 ± 15.5 | 2123.8 ± 74.3 | 1926.8 ± 28.9 | 1110.5 ± 18.9 | 627.7 ± 11.3 | 409.9 ± 6.1 | 371.7 ± 8.5 | 927.4 ± 29.7 | 904.6 ± 13.6 | 2045.2 ± 83.9 | 2201.1 ± 59.4 |
| **Aug. 24** | 1598.4 ± 24.0 | 768.7 ± 27.7 | 2057.5 ± 57.6 | 1992.8 ± 29.9 | 1075.9 ± 26.9 | 730.8 ± 8.0 | 445.9 ± 6.7 | 476.4 ± 17.1 | 993.2 ± 27.8 | 879.6 ± 13.2 | 2161.3 ± 73.5 | 2040.9 ± 34.7 |
| **Sep. 6** | 1573.9 ± 59.8 | 1026.4 ±22.6* | 2347.5 ± 91.6 | 2347.3 ± 49.3 | 1078.8 ± 20.5 | 734.6 ± 18.4 | 458.4 ± 17.4 | 516.2 ± 11.4 | 1027.5 ± 29.8 | 1054.5 ± 43.2 | 2233.3 ± 78.2 | 2085.9 ± 43.8 |
| **Sep. 20** | 1463.6 ± 42.4 | 959.7 ± 40.3* | 2717.2 ± 78.8 | 2314.7 ± 97.2 | 1190.4 ± 39.3 | 735.2 ± 12.5 | 578.6 ± 16.8 | 554.2 ± 23.3 | 1048.2 ± 40.9 | 966.9 ± 40.6 | 2769.4 ± 108.0 | 2109.8 ± 67.5 |
| **Oct. 4** | 1224.4 ± 19.6 | 842.5 ± 31.2 | 2540.2 ± 61.0 | 2257.3 ± 38.4 | 1046.4 ± 18.8 | 723.7 ± 10.9 | 561.0 ± 13.5 | 532.8 ± 22.4 | 1210.7 ± 29.1 | 974.4 ± 16.6 | 2660.6 ± 42.6 | 2315.3 ± 41.7 |
| **Oct. 19** | 1160.4 ± 39.5 | 823.6 ± 29.6 | 2800.4 ± 123.2 | 2190.0 ± 78.8 | 1018.6 ± 21.4 | 680.3 ± 12.9 | 633.6 ± 14.6 | 538.5 ± 11.8 | 1281.7 ± 56.4 | 985.0 ± 35.5 | 3022.3 ± 126.9 | 2413.5 ± 55.5 |
| **Nov. 1** | 1283.1 ± 19.2 | 772.8 ± 25.5 | 2826.0 ± 98.9 | 2277.4 ± 29.6 | 1066.0 ± 32.0 | 698.6 ± 10.5 | 703.6 ± 27.4 | 503.0 ± 14.6 | 1293.2 ± 45.3 | 1032.9 ± 13.4 | 3273.3 ± 104.7 | 2498.0 ± 47.5 |
| **Nov. 16** | 1202.0 ± 34.9 | 772.3 ± 25.5 | 2843.8 ± 139.3 | 2453.2 ± 81.0* | 1123.4 ± 25.8 | 651.3 ± 9.8 | 679.9 ± 22.4 | 539.5 ± 9.2 | 1396.2 ± 68.4 | 1068.5 ± 35.3 | 3165.7 ± 91.8 | 2683.0 ± 88.5* |
| **Nov. 29** | 1039.5 ± 43.7 | 734.3 ± 31.6 | 2790.7 ± 117.2 | 2583.8 ± 59.4* | 1046.1 ± 46.0 | 721.7 ± 7.9 | 647.9 ± 27.2 | 651.3 ± 15.0* | 1221.9 ± 51.3 | 1226.4 ± 15.9* | 3172.5 ± 149.1 | 2793.7 ± 69.9* |
| **Dec. 13** | 1006.5 ± 32.2 | 521.4 ± 20.3 | 2778.9 ± 88.9 | 1435.5 ± 56.0 | 973.5 ± 24.3 | 683.7 ± 11.6 | 640.5 ± 26.9 | 561.0 ± 16.3 | 1141.3 ± 36.5 | 1241.2 ± 48.4* | 3122.2 ± 131.1 | 2355.7 ± 54.2 |
| **Dec. 28** | 692.2 ± 20.1 | 502.5 ± 6.5 | 2526.2 ± 73.3 | 1624.2 ± 21.1 | 821.4 ± 20.5 | 721.4 ± 10.1 | 561.3 ± 16.3 | 560.7 ± 7.3 | 1158.4 ± 33.6 | 902.1 ± 11.7 | 3530.1 ± 113.0 | 2520.1 ± 47.9 |

Results are presented as means ± SD of the samples collected at different dates in a certain forest plot. Values marked with * in the same column indicate the highest ($p < 0.05$) amounts in samples.

**Table S6.** Contents of triterpenoid acids and sterols (μg/g DW ± SD) in young and old lingonberry leaves, collected throughout one year.

| Date | Maslinic acid | | Corosolic acid | | Betulinic acid | | Oleanolic acid | | Ursolic acid | | β-Sitosterol | |
|---|---|---|---|---|---|---|---|---|---|---|---|---|
| | Young leaves | Old leaves | Young leaves | Old leaves | Young leaves | Old leaves | Young leaves | Old leaves | Young leaves | Old leaves | Young leaves | Old leaves |
| **Jan. 11** | 138.8 ± 2.4 | 136.9 ± 5.2 | 68.9 ± 1.2 | 111.0 ± 4.2 | 15.7 ± 0.5 | 31.8 ± 1.1 | 107.9 ± 1.8 | 121.3 ± 2.2 | 504.3 ± 18.7 | 356.7 ± 6.4 | 221.4 ± 3.8 | 105.7 ± 1.9 |
| **Jan. 24** | 128.6 ± 5.4 | 136.8 ± 4.9 | 81.2 ± 1.8* | 152.7 ± 5.5* | 16.4 ± 0.2 | 22.3 ± 0.8 | 96.2 ± 4.0 | 109.2 ± 4.3 | 486.7 ± 20.4 | 435.9 ± 15.7 | 200.1 ± 8.4 | 118.4 ± 3.1 |
| **Feb. 7** | 128.6 ± 4.1 | 129.8 ± 5.1 | 75.4 ± 2.4 | 92.4 ± 3.6 | 7.6 ± 0.3 | 12.9 ± 0.1 | 94.8 ± 3.0 | 75.5 ± 2.2 | 380.8 ± 12.2 | 454.4 ± 13.2 | 140.1 ± 5.9 | 146.6 ± 4.3 |
| **Feb. 21** | 120.1 ± 3.5 | 132.8 ± 4.4 | 59.1 ± 1.7 | 95.8 ± 3.2 | 5.6 ± 0.1 | 13.9 ± 0.5 | 69.1 ± 2.0 | 83.1 ± 2.7 | 366.1 ± 7.0 | 461.2 ± 15.2 | 137.1 ± 2.6 | 161.8 ± 3.7 |
| **Mar. 7** | 122.5 ± 4.5 | 126.6 ± 6.1 | 51.5 ± 1.9 | 88.1 ± 1.6 | NQ | NQ | 55.5 ± 2.1 | 96.9 ± 1.7 | 317.0 ± 5.4 | 453.4 ± 12.7 | 109.4 ± 3.0 | 163.9 ± 4.6 |
| **Mar. 21** | 119.5 ± 3.6 | 121.7 ± 3.0 | 52.5 ± 1.7 | 41.0 ± 0.6 | NQ | NQ | 49.8 ± 1.5 | 93.9 ± 2.3 | 303.7 ± 9.1 | 468.1 ± 11.7 | 102.5 ± 4.5 | 153.8 ± 2.3 |
| **Apr. 4** | 126.6 ± 1.6 | 135.5 ± 3.9 | 48.4 ± 1.5 | 44.5 ± 1.1 | NQ | NQ | 49.5 ± 1.5 | 105.4 ± 2.6 | 306.3 ± 10.1 | 503.4 ± 19.6 | 101.0 ± 2.6 | 141.0 ± 3.5 |
| **Apr. 17** | 111.7 ± 3.7 | 141.6 ± 5.9 | 34.5 ± 1.0 | 47.4 ± 0.8 | NQ | NQ | 45.5 ± 1.4 | 117.4 ± 2.0 | 294.7 ± 9.7 | 543.3 ± 22.8 | 109.3 ± 4.5 | 141.6 ± 2.4 |
| **May 1** | 73.3 ± 3.2 | 98.5 ± 2.2 | 28 ± 0.7 | 46.4 ± 1.4 | NQ | NQ | 42.8 ± 1.1 | 119.8 ± 3.6 | 270.9 ± 11.7 | 624.9 ± 13.7 | 76.7 ± 1.2 | 143.8 ± 4.6 |
| **May 15** | 61.9 ± 1.4 | 80.6 ± 2.3 | 29.2 ± 0.6 | 58.1 ± 1.7 | NQ | NQ | 49.6 ± 0.9 | 103.5 ± 3.1 | 297.4 ± 6.5 | 569.2 ± 22.2 | 62.7 ± 1.2 | 153.5 ± 4.8 |
| **May 29** | 16.2 ± 0.4 | 23.6 ± 0.5 | 8.5 ± 0.2 | 44.2 ± 1.0 | NQ | NQ | 49.4 ± 1.2 | 106.4 ± 2.4 | 181.0 ± 5.4 | 588.2 ± 13.5 | 52.6 ± 1.3 | 162.0 ± 5.7 |
| **Jun. 13** | 3.2 ± 0.1 | 36.6 ± 1.6 | 9.7 ± 0.3 | 59.9 ± 2.9 | NQ | NQ | 62.3 ± 0.7 | 99.8 ± 4.7 | 286.2 ± 8.9 | 624.5 ± 28.1 | 196.1 ± 4.1 | 207.8 ± 8.1 |
| **Jun. 28** | 4.8 ± 0.2 | 45.8 ± 1.2 | 8.8 ± 0.2 | 62.7 ± 2.9 | NQ | NQ | 64.8 ± 2.2 | 111.9 ± 5.3 | 363.4 ± 8.7 | 647.2 ± 17.5 | 221.0 ± 6.9 | 200.3 ± 7.0 |
| **Jul. 15** | 6.3 ± 0.2 | 45.6 ± 1.6 | 10.6 ± 0.2 | 85.9 ± 2.1 | NQ | NQ | 70.6 ± 1.3 | 117.9 ± 2.9 | 369.5 ± 6.7 | 658.9 ± 29.7 | 228.4 ± 8 | 192.1 ± 3.1 |
| **Jul. 29** | 10.0 ± 0.2 | 52.2 ± 1.4 | 10.4 ± 0.3 | 86.5 ± 2.6 | NQ | NQ | 74.3 ± 2.2 | 120.6 ± 3.6 | 374.9 ± 6.0 | 638.0 ± 17.2 | 246.5 ± 9.4 | 218.0 ± 4.8 |
| **Aug. 11** | 17.7 ± 0.5 | 48.7 ± 1.8 | 22.4 ± 0.6 | 76.1 ± 1.8 | NQ | 8.5 ± 0.4 | 80.3 ± 2.0 | 131.0 ± 3.7 | 447.6 ± 13.9 | 636.1 ± 22.9 | 244.8 ± 7.8 | 208.9 ± 3.1 |
| **Aug. 24** | 18.9 ± 0.3 | 36.8 ± 1.3 | 19.3 ± 0.3 | 79.4 ± 2.9 | NQ | 9.8 ± 0.3 | 82.7 ± 1.2 | 132.9 ± 2.1 | 474.6 ± 7.1 | 685.7 ± 24.7 | 226.2 ± 6.3 | 206.3 ± 3.1 |
| **Sep. 6** | 16.1 ± 0.4 | 36.2 ± 1.1 | 22.2 ± 0.8 | 95.2 ± 3.5 | NQ | 10.7 ± 0.3 | 78.8 ± 1.2 | 142.6 ± 3.0 | 453.9 ± 15.9 | 690.7 ± 20.7 | 245.9 ± 7.1 | 255.1 ± 10.5 |
| **Sep. 20** | 17.7 ± 0.5 | 41.2 ± 1.7 | 21.5 ± 0.6 | 98.2 ± 4.1 | 13.1 ± 0.3 | 21.3 ± 0.8 | 93.0 ± 2.7 | 139.9 ± 5.9 | 500.9 ± 9.0 | 697.9 ± 29.3 | 259.3 ± 10.1 | 252.9 ± 10.6 |
| **Oct. 4** | 15.9 ± 0.3 | 43.3 ± 1.6 | 27.8 ± 0.4 | 88.0 ± 3.3 | 14.1 ± 0.3 | 21.2 ± 0.5 | 99.3 ± 1.6 | 164.6 ± 6.1 | 505.7 ± 8.1 | 832.1 ± 30.8 | 267.8 ± 3.7 | 294.6 ± 5.0 |
| **Oct. 19** | 18.2 ± 0.6 | 76.3 ± 2.7 | 25.7 ± 0.9 | 84.8 ± 3.1 | 11.2 ± 0.3 | 30.1 ± 0.8 | 101.2 ± 3.4 | 191.7 ± 6.9* | 554.9 ± 18.9 | 902.7 ± 32.5* | 276.2 ± 12.2 | 390.7 ± 18.0* |
| **Nov. 1** | 43.0 ± 0.6 | 67.6 ± 2.2 | 30.4 ± 0.5 | 81.0 ± 2.7 | 12.8 ± 0.3 | 28.0 ± 0.8 | 102.6 ± 1.5 | 185.0 ± 6.1* | 582.2 ± 8.7* | 834.8 ± 27.5 | 294.3 ± 4.4 | 375.5 ± 12.4* |
| **Nov. 16** | 62.1 ± 1.8 | 81.6 ± 2.7 | 36.3 ± 1.1 | 100.2 ± 3.3 | 18.6 ± 0.2 | 29.2 ± 0.5 | 102.2 ± 3.0 | 170.5 ± 7.6 | 559.9 ± 16.2 | 754.2 ± 24.9 | 431.2 ± 21.1* | 351.2 ± 8.1 |
| **Nov. 29** | 64.6 ± 2.7 | 154.3 ± 7.4 | 60.4 ± 1.9 | 100.3 ± 4.3 | 21.3 ± 0.5 | 29.8 ± 0.9 | 104.3 ± 4.0 | 178.1 ± 7.7 | 587.7 ± 24.7* | 722.6 ± 31.1 | 401.7 ± 16.9* | 314.8 ± 4.1 |
| **Dec. 13** | 104.6 ± 3.3 | 174.3 ± 3.8* | 56.4 ± 1.8 | 90.6 ± 3.5 | 33.7 ± 1.0* | 54.8 ± 1.4* | 125.7 ± 2.8* | 169.3 ± 6.6 | 606.6 ± 19.4* | 729.6 ± 28.5 | 307.4 ± 12.9 | 243.6 ± 9.5 |
| **Dec. 28** | 155.0 ± 7.6* | 136.9 ± 4.5 | 69.3 ± 2.0 | 88.8 ± 3.8 | 29.8 ± 0.6* | 50.8 ± 1.5* | 124.6 ± 3.6* | 142.4 ± 6.1 | 499.9 ± 14.5 | 526.0 ± 17.4 | 277.0 ± 8.0 | 159.1 ± 2.1 |

Results are presented as means ± SD of the samples collected at different dates in a certain forest plot. Values marked with * in the same column indicate the highest ($p < 0.05$) amounts in samples. NQ—not quantified (amount below LOQ).

**Table S7.** Contents of neutral triterpenoids (μg/g DW ± SD) in young and old lingonberry leaves, collected throughout one year.

| Date | Betulin | | Erythrodiol | | Uvaol | | Lupeol | | α-Amyrin | | β-Amyrin | | Friedelin | |
|---|---|---|---|---|---|---|---|---|---|---|---|---|---|---|
| | Young leaves | Old leaves | Young leaves | Old leaves | Young leaves | Old leaves | Young leaves | Old leaves | Young leaves | Old leaves | Young leaves | Old leaves | Young leaves | Old leaves |
| **Jan. 11** | 192.7 ± 3.3 | 131.5 ± 2.4 | 78.4 ± 1.3 | 95.5 ± 2.7 | 110.0 ± 4.6 | 129.4 ± 1.3 | 57.9 ± 0.9 | 118.8 ± 3 | 760.6 ± 14.5* | 456.8 ± 15.1 | 82.2 ± 1.4 | 52.3 ± 0.9 | 127.5 ± 6.0* | 38.0 ± 1.1 |
| **Jan. 24** | 206.5 ± 8.7 | 92.5 ± 2.4 | 78.4 ± 3.3 | 107.0 ± 3.9* | 124.5 ± 2.7* | 125.6 ± 3.6 | 41.4 ± 1.4 | 74.2 ± 2.2 | 801.8 ± 37.7* | 874.5 ± 21.9 | 91.6 ± 3.8 | 91.5 ± 3.3 | 93.1 ± 3.9 | 51.2 ± 1.8 |
| **Feb. 7** | 138.2 ± 4.4 | 92.0 ± 3.6 | 36.9 ± 1.2 | 47.9 ± 1.9 | 74.8 ± 2.2 | 143.0 ± 1.6 | 26.0 ± 0.9 | 8.1 ± 0.2 | 781.8 ± 25.8* | 857.9 ± 24.9 | 79.0 ± 3.3 | 89.4 ± 2.6 | 64.6 ± 2.1 | 61.5 ± 1.8 |
| **Feb. 21** | 141.9 ± 4.1 | 106.6 ± 3.5 | 24.6 ± 0.7 | 42.8 ± 1.4 | 33.8 ± 0.8 | 142.8 ± 3.4 | 21.6 ± 0.8 | 10.1 ± 0.1 | 721.5 ± 28.1 | 697.0 ± 16.0 | 68.5 ± 2.0 | 94.7 ± 3.1 | 60.7 ± 1.8 | 75.5 ± 2.5 |
| **Mar. 7** | 104.9 ± 3.9 | 90.7 ± 1.6 | 19.3 ± 0.7 | 43.1 ± 1.6 | 31.6 ± 0.8 | 151.5 ± 1.8 | 17.1 ± 0.3 | 10.5 ± 0.2 | 496.5 ± 8.4 | 911.5 ± 16.4 | 58.5 ± 1.6 | 94.4 ± 1.7 | 51.7 ± 1.9 | 68.7 ± 1.2 |
| **Mar. 21** | 99.0 ± 3.0 | 92.4 ± 2.3 | 14.6 ± 0.4 | 17.8 ± 0.4 | 24.5 ± 1.1 | 166.4 ± 7.5 | 18.6 ± 0.4 | 21.0 ± 0.5 | 481.4 ± 20.2 | 975.8 ± 24.4 | 59.1 ± 2.0 | 107.1 ± 1.6 | 41.9 ± 1.3 | 65.7 ± 1.6 |
| **Apr. 4** | 102.9 ± 3.1 | 90.8 ± 2.3 | 14.9 ± 0.3 | 20.3 ± 0.5 | 30.8 ± 1.0 | 155.6 ± 4.4 | 21.3 ± 0.6 | 23.4 ± 0.6 | 435.2 ± 13.1 | 979.1 ± 14.7 | 63.5 ± 2.7 | 118.9 ± 3.0 | 63.7 ± 1.4 | 70.7 ± 1.8 |
| **Apr. 17** | 100.1 ± 3.0 | 103.4 ± 1.8 | 14.1 ± 0.4 | 41.3 ± 0.7 | 28.8 ± 1.2 | 154.1 ± 5.5 | 21.9 ± 0.7 | 36.0 ± 1.0 | 279.0 ± 11.7 | 955.6 ± 25.8 | 50.5 ± 1.2 | 117.3 ± 2.0 | 43.4 ± 1.3 | 75.8 ± 1.3 |
| **May 1** | 81.3 ± 2.0 | 102.3 ± 3.1 | 13.4 ± 0.3 | 54.6 ± 1.6 | 23.6 ± 0.3 | 163.1 ± 6.4 | 20.7 ± 0.7 | 46.9 ± 1.4 | 297.0 ± 4.8 | 1059.0 ± 26.5 | 42.5 ± 0.6 | 111.8 ± 4.6 | 35.4 ± 0.9 | 66.0 ± 2.0 |
| **May 15** | 93.9 ± 1.8 | 82.9 ± 0.9 | 12.9 ± 0.2 | 33.8 ± 0.4 | 22.0 ± 0.4 | 116.7 ± 3.8 | 13.2 ± 0.3 | 48.0 ± 1.4 | 297.9 ± 5.4 | 870.1 ± 13.1 | 46.2 ± 0.9 | 106.1 ± 2.2 | 25.4 ± 0.5 | 71.4 ± 1.1 |
| **May 29** | 56.1 ± 1.4 | 40.2 ± 0.9 | 6.8 ± 0.2 | 31.6 ± 1.1 | 33.0 ± 1.1 | 144.8 ± 2.5 | 8.8 ± 0.3 | 42.9 ± 1.0 | 260.2 ± 7.5 | 936.3 ± 28.1 | 14.6 ± 0.4 | 114.0 ± 4.0 | 15.0 ± 0.5 | 76.4 ± 2.1 |
| **Jun. 13** | 64.4 ± 2.0 | 42.3 ± 1.6 | 5.8 ± 0.2 | 27.8 ± 0.5 | 26.1 ± 0.8 | 133.8 ± 3.5 | 12.0 ± 0.5 | 36.5 ± 1.0 | 239.5 ± 6.0 | 1308.9 ± 43.2 | 14.7 ± 0.2 | 114.3 ± 2.2 | 9.7 ± 0.1 | 76.6 ± 2.2 |
| **Jun. 28** | 51.4 ± 2.8 | 40.8 ± 1.5 | 5.6 ± 0.1 | 30.6 ± 1.1 | 51.5 ± 0.9 | 148.4 ± 2.2 | 25.9 ± 0.6 | 39.1 ± 0.7 | 338.6 ± 15.2 | 1341.5 ± 21.5 | 16.1 ± 0.2 | 131.3 ± 1.9* | 32.0 ± 0.4 | 107.7 ± 3.8 |
| **Jul. 15** | 53.2 ± 1.5 | 28.0 ± 1.0 | 5.0 ± 0.1 | 34.6 ± 1.2 | 50.8 ± 1.7 | 128.6 ± 5 | 22.1 ± 0.4 | 47.8 ± 0.7 | 345.7 ± 10.0 | 1327.6 ± 57.1 | 18.4 ± 0.5 | 107.5 ± 1.6 | 43.9 ± 1.0 | 93.0 ± 2.9 |
| **Jul. 29** | 47.7 ± 1.9 | 44.8 ± 0.9 | 4.8 ± 0.2 | 37.1 ± 0.8 | 46.6 ± 1.2 | 128.4 ± 4.2 | 22.9 ± 0.9 | 65.5 ± 2.0 | 300.3 ± 10.5 | 1389.5 ± 32.0 | 17.2 ± 0.7 | 107.5 ± 2.3 | 42.6 ± 0.7 | 93.0 ± 3.2 |
| **Aug. 11** | 65.9 ± 1.6 | 46.7 ± 1.1 | 6.2 ± 0.2 | 37.3 ± 0.6 | 44.5 ± 0.8 | 136.5 ± 2.5 | 29.9 ± 0.7 | 59.9 ± 1.4 | 368.0 ± 15.1 | 1336.0 ± 36.1 | 30.0 ± 0.4 | 120.6 ± 2.8 | 66.1 ± 1.1 | 104.0 ± 2.4 |
| **Aug. 24** | 63.7 ± 1.6 | 45.5 ± 0.7 | 36.8 ± 0.7 | 35.6 ± 1.2 | 54.9 ± 1.4 | 151.9 ± 4.7 | 36.4 ± 0.9 | 70.3 ± 2.5 | 381.6 ± 13.0 | 1417.1 ± 24.1 | 30.6 ± 0.5 | 122.2 ± 4.4 | 72.1 ± 1.1 | 101.8 ± 3.7 |
| **Sep. 6** | 53.0 ± 1.0 | 53.2 ± 2.2 | 42.3 ± 1.2 | 80.3 ± 1.7 | 55.8 ± 1.1 | 272.7 ± 6.8* | 42.4 ± 1.1 | 83.6 ± 1.8 | 366.6 ± 9.2 | 1954.7 ± 60.6* | 32.0 ± 1.2 | 126.8 ± 2.4 | 71.6 ± 1.8 | 143.3 ± 4.7 |
| **Sep. 20** | 80.1 ± 2.3 | 67.6 ± 1.5 | 51.3 ± 1.5 | 96.0 ± 4.0 | 68.0 ± 2.2 | 278.6 ± 9.9* | 54.3 ± 1.6 | 84.6 ± 3.6 | 469.4 ± 18.3 | 1450.0 ± 46.4 | 45.2 ± 1.3 | 119.7 ± 5.0 | 82.7 ± 1.6 | 141.8 ± 4.3 |
| **Oct. 4** | 83.3 ± 1.3 | 71.0 ± 1.2 | 49.8 ± 1.2 | 98.2 ± 1.7 | 69.0 ± 1.2 | 267.0 ± 6.7* | 53.4 ± 0.9 | 88.4 ± 1.6 | 465.8 ± 7.5 | 1198.8 ± 21.6 | 48.9 ± 1.2 | 117.6 ± 3.8 | 88.4 ± 1.4 | 142.9 ± 3.9 |
| **Oct. 19** | 120.6 ± 4.1 | 101.0 ± 3.6 | 59.8 ± 2.6 | 109.6 ± 3.9* | 82.5 ± 1.7 | 234.8 ± 4.5 | 66.1 ± 2.8 | 80.9 ± 3.5 | 611.8 ± 13.5 | 1226.4 ± 40.5 | 53.8 ± 1.2 | 122.8 ± 2.7 | 87.7 ± 2.1 | 150.6 ± 2.4* |
| **Nov. 1** | 115.8 ± 2.9 | 98.2 ± 1.3 | 56.3 ± 1.4 | 108.1 ± 3.6* | 81.2 ± 1.8 | 215.3 ± 7.5 | 52.1 ± 1.7 | 90.2 ± 1.7 | 621.9 ± 19.9 | 1324.3 ± 25.2 | 72.4 ± 2.8 | 120.5 ± 3.5 | 83.6 ± 1.3 | 141.9 ± 4.7 |
| **Nov. 16** | 179.3 ± 5.2 | 101.8 ± 3.4 | 53.3 ± 2.6 | 106.7 ± 3.5* | 89.0. ± 2.0 | 171.9 ± 2.6 | 94.4 ± 2.7* | 145.4 ± 4.8* | 683.9 ± 19.8 | 1250.4 ± 41.3 | 98.4 ± 3.2* | 107.4 ± 1.8 | 87.2 ± 2.5 | 93.2 ± 3.1 |
| **Nov. 29** | 179.3 ± 7.5 | 129.1 ± 4.3 | 53.4 ± 1.2 | 107.3 ± 3.5* | 78.6 ± 1.9 | 147.9 ± 4.6 | 94.4 ± 1.6* | 141.3 ± 4.9* | 710.8 ± 33.4 | 696.2 ± 17.4 | 98.6 ± 4.1* | 110.5 ± 2.5 | 95.3 ± 4.0 | 72.9 ± 3.1 |
| **Dec. 13** | 272.8 ± 8.7* | 163.7 ± 3.1* | 88.9 ± 2.8* | 93.9 ± 3.7 | 98.4 ± 1.5 | 131.7 ± 2.2 | 96.8 ± 2.1* | 145.4 ± 5.7* | 717.1 ± 8.6 | 783.3 ± 18.0 | 84.8 ± 3.6 | 107.3 ± 3.1 | 102.0 ± 3.3 | 74.2 ± 1.4 |
| **Dec. 28** | 201.6 ± 5.8 | 138.9 ± 1.8 | 85.8 ± 1.6* | 100.8 ± 3.3 | 105.3 ± 1.6 | 117.3 ± 1.6 | 69.0 ± 2.7 | 122.5 ± 5.4 | 785.9 ± 25.1* | 692.2 ± 13.2 | 89.4 ± 2.6 | 82.3 ± 1.1 | 135.1 ± 4.6* | 63.0 ± 0.8 |

Results are presented as means ± SD of the samples collected at different dates in a certain forest plot. Values marked with * in the same column indicate the highest ($p < 0.05$) amounts in samples.

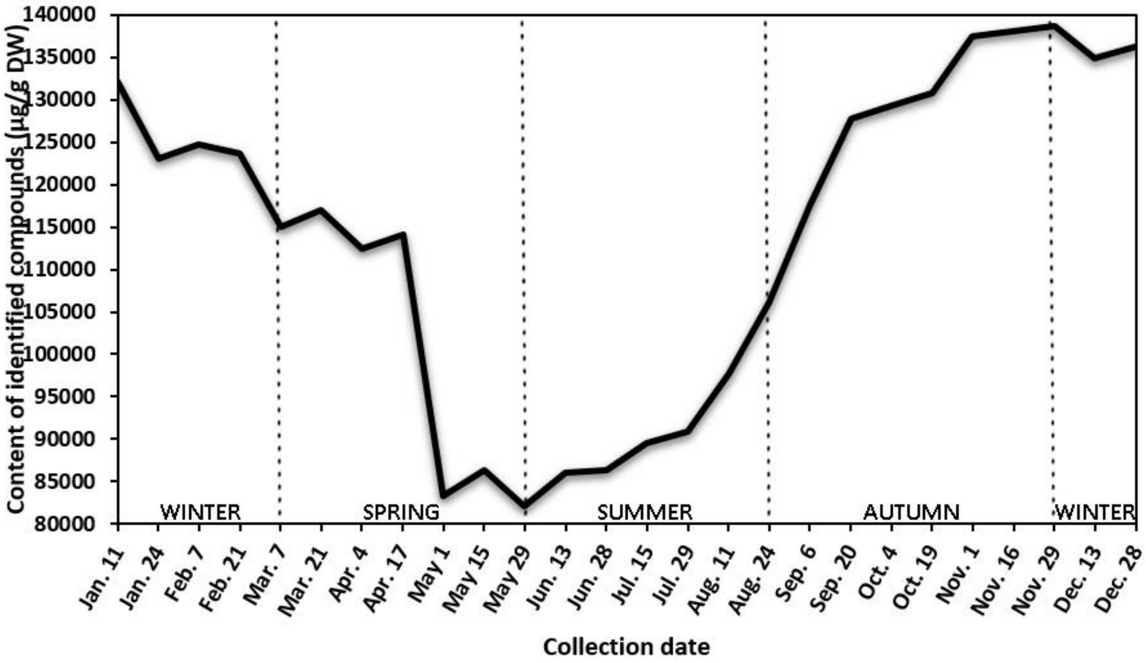


**Figure S1.** Variation of total identified secondary metabolites (µg/g DW) in lingonberry leaves, collected throughout one year.

**Table S8.** Contents of simple phenolics and A-type proanthocyanidins (μg/g DW ± SD) in lingonberry leaves, collected at different locations.

| Location | Arbutin | Hydroquinone | 2-*O*-Caffeoylarbutin | Procyanidin A1 | Procyanidin A2 | Procyanidin A4 |
|---|---|---|---|---|---|---|
| **Apūniškis** | 65172.6 ± 1448.0 | 121.9 ± 2.5 | 3375.1 ± 152.8 | 5079.8 ± 36.8 | 797.4 ± 52.7 | 72.9 ± 3.0 |
| **Plunksnuočiai** | 58029.1 ± 2840.6 | 423.2 ± 14.4* | 1564.5 ± 77.3 | 4421.8 ± 169.4 | 1009.8 ± 25.6 | 37.8 ± 3.9 |
| **Šakarva** | 63442.3 ± 2851.0 | 128.6 ± 2.7 | 2649.2 ± 92.3 | 4515.8 ± 271.6 | 1115.3 ± 72.9 | 63.8 ± 1.8 |
| **Andrioniškis** | 62054.5 ± 2476.8 | 160.9 ± 2.9 | 2078.9 ± 98.4 | 3965.0 ± 251.5 | 824.0 ± 66.1 | 51.5 ± 2.1 |
| **Jurgionys** | 98000.9 ± 497.9* | 269.3 ± 16.3 | 4039.4 ± 171.3 | 5025.8 ± 315.7 | 1187.7 ± 66.5 | 56.1 ± 3.3 |
| **Kernai** | 70986.5 ± 1830.9 | 215.6 ± 6.7 | 4288.6 ± 98.5* | 4103.8 ± 84.1 | 622.0 ± 19.4 | 36.0 ± 0.8 |
| **Žadeikiai** | 67230.0 ± 3094.0 | 83.7 ± 2.6 | 3265.8 ± 193.4 | 4082.7 ± 226.1 | 802.9 ± 45.0 | 62.1 ± 3.6 |
| **Galvokai** | 75229.3 ± 1447.8 | 92.3 ± 0.3 | 4507.0 ± 239.3* | 3592.1 ± 60.9 | 334.9 ± 0.8 | 25.4 ± 0.4 |
| **Giteniškė** | 59305.5 ± 1944.2 | 449.6 ± 2.1* | 842.7 ± 36.7 | 4943.0 ± 182.7 | 722.3 ± 18.7 | 99.9 ± 3.7* |
| **Šalčininkėliai** | 67886.3 ± 2689.8 | 93.2 ± 0.1 | 2338.2 ± 73.9 | 4573.5 ± 168.8 | 762.2 ± 38.6 | 67.9 ± 2.6 |
| **Bitėnai** | 46681.1 ± 423.1 | 217.8 ± 0.9 | 1742.4 ± 18.7 | 3411.4 ± 22.3 | 540.1 ± 18.1 | 31.0 ± 0.4 |
| **Pagramantis** | 70039.5 ± 1212.1 | 92.6 ± 2.9 | 2517.5 ± 53.1 | 3298.2 ± 2.6 | 4677.8 ± 0.3* | 68.5 ± 2.1 |
| **Kūprė** | 54883.3 ± 1653.7 | 260.9 ± 13.2 | 1481.6 ± 13.9 | 4927.3 ± 118.1 | 682.9 ± 4.4 | 95.6 ± 4.1 |
| **Bruknynė** | 78305.7 ± 2069.3 | 144.6 ± 5.9 | 2886 ± 133.9 | 5235.8 ± 97.3* | 959.5 ± 51.6 | 78.0 ± 4.9 |
| **Viršilai** | 68555.9 ± 743.6 | 179.0 ± 3.9 | 1902.9 ± 51.1 | 5193.5 ± 158.7* | 1125.0 ± 42.6 | 53.3 ± 0.9 |
| **Labanoras (a)** | 61384.8 ± 811.0 | 259.4 ± 7.5 | 40.7 ± 0.1 | 3985.0 ± 59.5 | 1661.7 ± 42.5 | 96.6 ± 4.0* |
| **Labanoras (b)** | 65985.3 ± 859.9 | 141.5 ± 2.6 | 3117.3 ± 118.7 | 4669.9 ± 60.8 | 936.0 ± 20.5 | 84.3 ± 3.3 |
| **Komarinė** | 51452.3 ± 616.9 | 210.9 ± 1.4 | 1581.8 ± 34.7 | 3829.6 ± 37.3 | 662.3 ± 2.5 | 37.4 ± 1.5 |
| **Marcinkonys** | 58928.3 ± 2507.3 | 246.6 ± 5.2 | 1583.7 ± 58.1 | 4000.9 ± 23.3 | 782.2 ± 4.6 | 47.4 ± 2.3 |
| **Šilainė** | 70974 ± 3069.6 | 289.8 ± 14.2 | 525.9 ± 28.4 | 5060.3 ± 182.6 | 625.1 ± 10.0 | 88.8 ± 5.1 |
| **Smėlynė** | 75292.1 ± 4063.1 | 123.6 ± 6.9 | 3252.9 ± 13.6 | 4748.3 ± 192.9 | 1680.1 ± 73.4 | 85.3 ± 0.5 |
| **Ilgalaukiai** | 64711.6 ± 2566.1 | 235.2 ± 12.2 | 2572.0 ± 67.1 | 5723.2 ± 243.0* | 1271.1 ± 66.7 | 76.7 ± 5.2 |
| **Vosniūnai** | 70645.2 ± 2058.3 | 72.3 ± 1.5 | 3526.4 ± 120.2 | 4621.1 ± 77.6 | 508.2 ± 16.3 | 63.1 ± 0.8 |
| **Kukuliškiai** | 61503.5 ± 2353.3 | 173.3 ± 8.9 | 1507.6 ± 66.1 | 4426.7 ± 207.9 | 630.3 ± 43.9 | 44.3 ± 2.4 |
| **Tolkūnai** | 85325.6 ± 2614.3 | 190.1 ± 5.4 | 3015.7 ± 131.2 | 5096.4 ± 208.5 | 1220.6 ± 99.8 | 85.8 ± 1.8 |
| **Bakūriškis** | 70383.1 ± 2201.9 | 231.9 ± 5.2 | 3394.7 ± 115.7 | 2474.9 ± 86.1 | 2280.1 ± 120.2 | 44.0 ± 1.3 |
| **Šilinė** | 62953.5 ± 1657.3 | 215.4 ± 5.0 | 1874.8 ± 85.3 | 3553.1 ± 84.1 | 499.6 ± 3.9 | 36.2 ± 1.5 |
| **Tyrelis** | 71061.4 ± 1029.7 | 139.5 ± 2.0 | 2684.5 ± 75.3 | 3257.1 ± 92.2 | 4546.9 ± 88.6* | 107.9 ± 4.4* |
| **Pažemys** | 40280.9 ± 1071.5 | 152.9 ± 1.5 | 1403.5 ± 68.5 | 4411.7 ± 130.2 | 625.7 ± 16.7 | 62.3 ± 1.0 |

Results are presented as means ± SD of the samples collected at different locations at the end of September 2019. Values marked with * in the same column indicate the highest ($p < 0.05$) amounts in samples.

**Table S9.** Contents of catechins and B-type proanthocyanidins (µg/g DW ± SD) in lingonberry leaves, collected at different locations.

| Location | (+)-Catechin | (−)-Epicatechin | Procyanidin B1 | Procyanidin B2 | Procyanidin B3 | Procyanidin C1 |
|---|---|---|---|---|---|---|
| Apūniškis | 13297.9 ± 124.7* | 3375.1 ± 152.8 | 5810.5 ± 23.7* | 2294.4 ± 63.0 | 6874.0 ± 214.4* | 4130.0 ± 148.6 |
| Plunksnuočiai | 6652.1 ± 158.9 | 1564.5 ± 77.3 | 3118.3 ± 82.1 | 2079.4 ± 41.4 | 4209.0 ± 213.8 | 2929.4 ± 138.9 |
| Šakarva | 9848.1 ± 397.9 | 2649.2 ± 15.5 | 4422.0 ± 28.6 | 2597.2 ± 107.1 | 4975.3 ± 160.1 | 3024.7 ± 195.1 |
| Andrioniškis | 7432.6 ± 189.1 | 2078.9 ± 98.4 | 3325.9 ± 129.1 | 2183.5 ± 43.3 | 4225.0 ± 22.4 | 2762.6 ± 21.1 |
| Jurgionys | 10043.1 ± 357.6 | 4039.4 ± 225.0 | 3748.7 ± 147.7 | 2564.2 ± 125.8 | 4240.0 ± 212.5 | 3465.8 ± 44.5 |
| Kernai | 7360.1 ± 48.9 | 4288.6 ± 98.5* | 5100.0 ± 182.7 | 1401.5 ± 19.1 | 3624.6 ± 179.0 | 2927.1 ± 4.3 |
| Žadeikiai | 11232.0 ± 119.6 | 3265.8 ± 116.6 | 3243.8 ± 118.8 | 2934.2 ± 153.7 | 5990.0 ± 331.2 | 3307.7 ± 146 |
| Galvokai | 11192.2 ± 497.8 | 4506.9 ± 85.7* | 3743.5 ± 133.5 | 1428.9 ± 3.5 | 5087.3 ± 88.1 | 2423.3 ± 46.6 |
| Giteniškė | 9930.6 ± 235.3 | 842.7 ± 36.7 | 3835.5 ± 138.6 | 1283.9 ± 20.1 | 4258.7 ± 201.3 | 2404.7 ± 100.4 |
| Šalčininkėliai | 12078.2 ± 428.1 | 2338.2 ± 73.9 | 4248.1 ± 188.6 | 2548.7 ± 62.7 | 6231.6 ± 297.1 | 2861.8 ± 121.0 |
| Bitėnai | 3429.6 ± 127 | 1742.4 ± 18.7 | 1112.4 ± 19.7 | 1430.4 ± 40.5 | 2818.6 ± 16.7 | 1818.4 ± 15.1 |
| Pagramantis | 7353.1 ± 253.3 | 2517.5 ± 53.1 | 4843.7 ± 231.5 | 6990.2 ± 129.2* | 3822.8 ± 55.6 | 4392.1 ± 80.6 |
| Kūprė | 9301.8 ± 259.8 | 1481.6 ± 90.6 | 5099.7 ± 207.8 | 1750.0 ± 97.1 | 5937.2 ± 100.2 | 3375.7 ± 162.3 |
| Bruknynė | 12828.7 ± 239.2 | 2886.0 ± 133.9 | 4831.9 ± 123.8 | 2874.0 ± 59.0 | 6767.8 ± 83.1* | 3293.7 ± 162.7 |
| Viršilai | 7535.7 ± 227.3 | 1957.2 ± 51.2 | 3842.0 ± 177.8 | 1891.5 ± 63.9 | 3389.3 ± 170.6 | 2433.5 ± 70.2 |
| Labanoras (a) | 9255.6 ± 26.4 | 40.7 ± 0.1 | 5172.6 ± 133.0 | 4281.5 ± 130.4 | 5193.4 ± 89.4 | 2519.4 ± 126.6 |
| Labanoras (b) | 11614.9 ± 444.7 | 3117.2 ± 42.0 | 6181.6 ± 110.4* | 2629.2 ± 49.0 | 5834.7 ± 144.6 | 3837.1 ± 82.5 |
| Komarinė | 6188.1 ± 154.2 | 1581.8 ± 34.7 | 2853.5 ± 64.2 | 1615.7 ± 33.9 | 3304.7 ± 33.1 | 2529.6 ± 0.2 |
| Marcinkonys | 8306.6 ± 263.9 | 1583.7 ± 58.1 | 4465.1 ± 187.2 | 2599.6 ± 55.5 | 4966.6 ± 44.3 | 3233.2 ± 71.8 |
| Šilainė | 14101.3 ± 443.6* | 525.9 ± 28.4 | 4684.7 ± 88.3 | 2403.5 ± 80.1 | 7135.9 ± 202.6* | 2894.2 ± 191.2 |
| Smėlynė | 13235.7 ± 187.9* | 3252.9 ± 90.4 | 6044.4 ± 143.5* | 3832.2 ± 168.9 | 6213.6 ± 330.9 | 5053.3 ± 137.9* |
| Ilgalaukiai | 12446.1 ± 143.9 | 2572.0 ± 67.1 | 4069.5 ± 176.9 | 2479.9 ± 99.7 | 5732.8 ± 125.2 | 3178.9 ± 142.6 |
| Vosniūnai | 11493.0 ± 148.5 | 3526.4 ± 43.4 | 4354.3 ± 98.6 | 1483.1 ± 33.6 | 4640.4 ± 182.6 | 3719.1 ± 95.2 |
| Kadagynai | 7637.5 ± 347.7 | 1507.6 ± 66.1 | 3815.7 ± 76.4 | 1640.7 ± 39.4 | 4798.2 ± 242.2 | 3048.2 ± 146.4 |
| Tolkūnai | 11483.9 ± 178.1 | 3015.7 ± 131.2 | 4864.7 ± 119.4 | 2877.0 ± 146.7 | 4870.3 ± 199.4 | 4620.5 ± 59.8* |
| Bakūriškis | 6335.4 ± 198.9 | 3394.7 ± 115.7 | 3670.7 ± 149.1 | 5318.1 ± 163.1 | 3690.7 ± 83.5 | 4292.3 ± 234.8 |
| Šilinė | 7711.0 ± 141.8 | 1847.7 ± 46.9 | 4305.2 ± 50.8 | 1652.5 ± 88.8 | 4717.8 ± 65.8 | 2777.6 ± 122.3 |
| Tyrelis | 7951.3 ± 262.5 | 2684.5 ± 75.3 | 5120.1 ± 142.6 | 6883.3 ± 225.3* | 3768.3 ± 91.3 | 4237.6 ± 78.3 |
| Pažemys | 10598.9 ± 148.9 | 1403.5 ± 68.5 | 3418.8 ± 161.3 | 1736.4 ± 69.5 | 5933.4 ± 64.3 | 2697.2 ± 134.8 |

Results are presented as means ± SD of the samples collected at different locations at the end of September 2019. Values marked with * in the same column indicate the highest ($p < 0.05$) amounts in samples.

**Table S10.** Contents of flavonol aglycones and phenolic acids (µg/g DW ± SD) in lingonberry leaves, collected at different locations.

| Location | Quercetin | Kaempferol | Chlorogenic acid | Cryptochlorogenic acid | Neochlorogenic acid | *p*-Coumaric acid |
|---|---|---|---|---|---|---|
| **Apūniškis** | 69.1 ± 4.1 | 3.8 ± 0.1 | 553.6 ± 10.4 | 1044.5 ± 11.9 | 58.0 ± 1.9 | 382.9 ± 20.3* |
| **Plunksnuočiai** | 58.8 ± 0.9 | ND | 3382.1 ± 115.2* | 326.8 ± 11.7 | 24.8 ± 0.2 | 351.0 ± 18.1 |
| **Šakarva** | 64.5 ± 0.2 | ND | 2223.7 ± 52.1 | 405.4 ± 22.9 | 30.8 ± 1.0 | 287.1 ± 8.0 |
| **Andrioniškis** | 70.5 ± 2.0 | 4.3 ± 0.1 | 1176.4 ± 8.2 | 936.9 ± 54.4 | 47.6 ± 2.7 | 214.4 ± 3.1 |
| **Jurgionys** | 67.2 ± 3.5 | NQ | 152.4 ± 3.4 | 784.6 ± 16.7 | 59.2 ± 1.1 | 143.4 ± 4.4 |
| **Kernai** | 62.3 ± 0.5 | 3.8 ± 0.0 | 2430.4 ± 7.8 | 798.9 ± 4.4 | 42.7 ± 0.1 | 130.8 ± 1.5 |
| **Žadeikiai** | 75.8 ± 1.9 | NQ | 489.0 ± 19.3 | 1008.8 ± 35.5 | 109.7 ± 4.3 | 129.0 ± 5.2 |
| **Galvokai** | 71.7 ± 2.8 | ND | 464.8 ± 11.4 | 1471.1 ± 14.6 | 138.4 ± 0.1 | 254.2 ± 6.7 |
| **Giteniškė** | 75.5 ± 1.7 | 3.9 ± 0.1 | 906.0 ± 37.7 | 563.8 ± 17.3 | 39.2 ± 0.5 | 262.6 ± 12.5 |
| **Šalčininkėliai** | 73.7 ± 0.6 | 4.2 ± 0.0 | 745.5 ± 26.2 | 963.0 ± 51.5 | 90.2 ± 4.7 | 271.9 ± 2.9 |
| **Bitėnai** | 62.5 ± 0.4 | NQ | 321.7 ± 2.9 | 161.7 ± 0.9 | 21.4 ± 1.4 | 289.6 ± 3.4 |
| **Pagramantis** | 64.1 ± 0.2 | ND | 1481.8 ± 10.8 | 468.1 ± 3.1 | 42.8 ± 0.3 | 254.3 ± 1.7 |
| **Kūprė** | 80.1 ± 3.8 | 4.2 ± 0.1 | 1473.0 ± 41.3 | 947.2 ± 32.4 | 46.3 ± 1.4 | 342.3 ± 8.4 |
| **Bruknynė** | 77.9 ± 0.7 | 4.2 ± 0.3 | 723.7 ± 16.9 | 998.8 ± 1.5 | 97.7 ± 4.4 | 295.5 ± 10.9 |
| **Viršilai** | 62.5 ± 2.7 | NQ | 3188.6 ± 153.0 | 670.8 ± 1.3 | 37.4 ± 3.0 | 156.6 ± 4.0 |
| **Labanoras (a)** | 73.3 ± 0.2 | 3.9 ± 0.2 | 3510.7 ± 22.0* | 386.1 ± 0.4 | 66.7 ± 2.7 | 98.1 ± 0.5 |
| **Labanoras (b)** | 75.6 ± 0.8 | 3.9 ± 0.2 | 3346.6 ± 71.9* | 453.5 ± 8 | 30.6 ± 0.5 | 238 ± 5.7 |
| **Komarinė** | 78.1 ± 1.3 | 4.2 ± 0.2 | 1690.9 ± 46.4 | 450.8 ± 5.5 | 32.7 ± 0.8 | 179.4 ± 1.1 |
| **Marcinkonys** | 121.1 ± 5.9* | 7.8 ± 0.4* | 1485.1 ± 36.7 | 240.0 ± 2.5 | 27.1 ± 0.2 | 199.8 ± 3.1 |
| **Šilainė** | 92.5 ± 1.3 | 5.4 ± 0.2 | 2104.1 ± 122.4 | 598.1 ± 10.5 | 38.5 ± 1.2 | 365.2 ± 22.0 |
| **Smėlynė** | 90.6 ± 1.0 | 4.1 ± 0.1 | 3304.2 ± 163.9* | 1830.1 ± 20.3* | 113.8 ± 0.7 | 353.0 ± 7.4 |
| **Ilgalaukiai** | 69.6 ± 1.5 | 4.1 ± 0.2 | 799.8 ± 36.4 | 991.0 ± 29.6 | 44.6 ± 0.3 | 245.0 ± 8.0 |
| **Vosniūnai** | 70.4 ± 0.9 | 3.7 ± 0.2 | 913.7 ± 26.8 | 849.5 ± 18.0 | 70.5 ± 3.4 | 252.9 ± 7.5 |
| **Kadagynai** | 73.1 ± 1.9 | 4.1 ± 0.1 | 2192.5 ± 89.7 | 693.6 ± 21.1 | 41.0 ± 2.1 | 223.9 ± 4.9 |
| **Tolkūnai** | 82.4 ± 4.0 | 4.1 ± 0.2 | 395.1 ± 16.2 | 470.9 ± 28.8 | 46.3 ± 1.8 | 411.9 ± 10.7* |
| **Bakūriškis** | 94.4 ± 1.1 | 4.5 ± 0.0 | 151.6 ± 0.2 | 1532.4 ± 32.9 | 161.7 ± 1.2* | 400.3 ± 15.5* |
| **Šilinė** | 72.8 ± 1.7 | 3.7 ± 0.2 | 500.2 ± 29.3 | 1002.4 ± 9.9 | 89.7 ± 5.3 | 203.4 ± 10.1 |
| **Tyrelis** | 65.2 ± 0.4 | 3.6 ± 0.1 | 1157.2 ± 10.8 | 543.0 ± 8.8 | 38.3 ± 1.3 | 270.9 ± 6.5 |
| **Pažemys** | 85.8 ± 0.6 | 4.8 ± 0.0 | 1362.7 ± 63.3 | 1477.1 ± 14.3 | 75.5 ± 4.3 | 154.9 ± 3.4 |

Results are presented as means ± SD of the samples collected at different locations at the end of September 2019. Values marked with * in the same column indicate the highest ($p < 0.05$) amounts in samples. ND—not detected, NQ—not quantified (amount below LOQ).

**Table S11.** Contents of kaempferol and quercetin glycosides (μg/g DW ± SD) in lingonberry leaves, collected at different locations.

| Location | Nicotiflorin | Astragalin | Afzelin | Quercitrin | Quercetin-HMG-rhamnoside | 6”-*O*-acetylisoquercitrin |
|---|---|---|---|---|---|---|
| **Apūniškis** | 66.6 ± 3.3 | 73.8 ± 4.2 | 66.8 ± 2.6 | 889.9 ± 45.9 | 1284.6 ± 44.7 | 52.2 ± 2.5 |
| **Plunksnuočiai** | 3.9 ± 0.8 | 10.3 ± 0.1 | 37.2 ± 1.5 | 637.4 ± 32.7 | 1092.3 ± 48.2 | 10.4 ± 0.2 |
| **Šakarva** | 72.5 ± 2.1 | 31.4 ± 1.2 | 101.2 ± 1.4 | 1449.8 ± 40.2 | 1197.2 ± 22.1 | 26.7 ± 0.7 |
| **Andrioniškis** | 104.4 ± 5.3 | 50.8 ± 2.6 | 58.5 ± 0.1 | 1005.9 ± 24.5 | 1327.0 ± 40.8 | 32.5 ± 1.0 |
| **Jurgionys** | 122.1 ± 5.9 | 81.1 ± 2.5 | 51.3 ± 1.3 | 296.2 ± 33.9 | 741.5 ± 9.8 | 26.8 ± 0.6 |
| **Kernai** | 78.7 ± 5.4 | 55.5 ± 2.6 | 124.1 ± 4.0 | 1240.0 ± 60.7 | 1685.1 ± 84.7 | 21.6 ± 1.1 |
| **Žadeikiai** | NQ | 21.6 ± 0.3 | 47.0 ± 3.5 | 1067.6 ± 16.9 | 2022.0 ± 66.3 | 25.4 ± 0.9 |
| **Galvokai** | 3.3 ± 1.8 | 35.0 ± 1.5 | 69.8 ± 1.5 | 950.2 ± 37.9 | 1392.0 ± 46.4 | 20.2 ± 0.1 |
| **Giteniškė** | 52.0 ± 2.7 | 42.8 ± 2.4 | 40.9 ± 2.4 | 840.9 ± 42.5 | 1092.8 ± 68.1 | 31.7 ± 1.2 |
| **Šalčininkėliai** | 78.6 ± 1.0 | 61.0 ± 0.4 | 70.8 ± 1.9 | 974.1 ± 12.1 | 1551.9 ± 33.7 | 46.6 ± 2.8 |
| **Bitėnai** | 34.5 ± 1.2 | 18.0 ± 0.0 | 26.5 ± 1.1 | 703.7 ± 2.3 | 778.2 ± 8.7 | 11.2 ± 0.1 |
| **Pagramantis** | 87.5 ± 4.0 | 60.1 ± 2.5 | 63.4 ± 2.1 | 840.3 ± 36.6 | 1394.2 ± 50.6 | 28.9 ± 1.6 |
| **Kūprė** | 4.1 ± 0.6 | 16.9 ± 0.2 | 58.5 ± 3.6 | 845.1 ± 28.8 | 1738.6 ± 76.6 | 56.7 ± 2.6 |
| **Bruknynė** | 98.4 ± 2.5 | 70.7 ± 1.7 | 74.9 ± 0.9 | 967.9 ± 222.1 | 1584.2 ± 21.8 | 47.9 ± 0.4 |
| **Viršilai** | NQ | 6.6 ± 0.7 | 37.8 ± 2.9 | 699.9 ± 36.9 | 1214.0 ± 20.3 | 18.3 ± 0.3 |
| **Labanoras (a)** | 49.3 ± 0.2 | 32.6 ± 0.2 | 89.4 ± 0.2 | 3125.3 ± 17.8* | 71.3 ± 0.9 | 51.6 ± 0.2 |
| **Labanoras (b)** | 84.5 ± 3.1 | 54.6 ± 1.6 | 141.9 ± 8.1 | 1962.1 ± 76.2 | 898.7 ± 17.3 | 46.6 ± 2.6 |
| **Komarinė** | 157.8 ± 13.1 | 79.7 ± 2.8 | 56.1 ± 0.9 | 980.5 ± 65.3 | 1537.4 ± 28.3 | 23.9 ± 0.9 |
| **Marcinkonys** | 187.1 ± 12.6 | 89.8 ± 1.3 | 96.1 ± 2.0 | 1584.3 ± 88.5 | 1051.9 ± 62.4 | 73.0 ± 2.7 |
| **Šilainė** | 128.4 ± 7.5 | 65.5 ± 4.0 | 28.7 ± 1.6 | 514.5 ± 20.1 | 965.2 ± 26.3 | 39.0. ± 1.4 |
| **Smėlynė** | 329.5 ± 13.5* | 182.7 ± 5.0 | 71.6 ± 0.5 | 873.4 ± 47.9 | 1579.1 ± 34.8 | 90.1 ± 2.0* |
| **Ilgalaukiai** | 132.0 ± 3.2 | 81.3 ± 7.5 | 90.4 ± 3.3 | 1621.4 ± 47.5 | 2005.9 ± 83.9 | 32.3 ± 0.6 |
| **Vosniūnai** | 197.3 ± 9.6 | 100.9 ± 4.6 | 69.4 ± 2.3 | 855.2 ± 57.5 | 1288.5 ± 63.2 | 36.1 ± 0.2 |
| **Kadagynai** | 175.8 ± 6.5 | 80.4 ± 3.3 | 58.9 ± 2.3 | 981.6 ± 13.4 | 1633.4 ± 34.7 | 30.8 ± 1.8 |
| **Tolkūnai** | 293.5 ± 4.9 | 226.9 ± 5.5* | 49.3 ± 1.1 | 1607.3 ± 59.8 | 1948.8 ± 61.9 | 46.4 ± 0.2 |
| **Bakūriškis** | NQ | 28.3 ± 1.8 | 201.0 ± 2.3* | 2437.6 ± 12.8 | 2684.9 ± 12.1* | 31.7 ± 1.0 |
| **Šilinė** | 184.2 ± 9.9 | 69.4 ± 4.0 | 45.3 ± 1.5 | 578.1 ± 20.0 | 882.3 ± 26.6 | 37.8 ± 0.7 |
| **Tyrelis** | 78.4 ± 1.4 | 54.1 ± 3.3 | 58.2 ± 2.1 | 710.5 ± 37.9 | 1226.4 ± 60.1 | 23.7 ± 0.8 |
| **Pažemys** | 167.2 ± 5.0 | 119.5 ± 2.7 | 103.9 ± 3.7 | 1526.7 ± 31.4 | 2127.3 ± 46.0 | 79.7 ± 2.4 |

Results are presented as means ± SD of the samples collected at different locations at the end of September 2019. Values marked with * in the same column indicate the highest ($p < 0.05$) amounts in samples. NQ—not quantified (amount below LOQ).

**Table S12.** Contents of quercetin glycosides (µg/g DW ± SD) in lingonberry leaves, collected at different locations.

| Location | Rutin | Hyperoside | Isoquercitrin | Reynoutrin | Guaiaverin | Avicularin |
|---|---|---|---|---|---|---|
| **Apūniškis** | 806.4 ± 57.0 | 2285.6 ± 136.3 | 714.3 ± 17.9 | 515.1 ± 16.8 | 1043.5 ± 16.9 | 2376.2 ± 69.0 |
| **Plunksnuočiai** | 154.2 ± 11.4 | 213.3 ± 14.1 | 80.9 ± 4.6 | 76.5 ± 1.0 | 98.9 ± 6.7 | 351.9 ± 21.6 |
| **Šakarva** | 595.1 ± 20.6 | 654.0 ± 18.4 | 234.0 ± 6.6 | 250.5 ± 7.0 | 295.2 ± 7.7 | 1067.6 ± 33.4 |
| **Andrioniškis** | 964.4 ± 54.0 | 1037 ± 44.7 | 400.1 ± 25.3 | 388.0 ± 17.8 | 481.1 ± 19.6 | 1420.8 ± 6.7 |
| **Jurgionys** | 974.8 ± 12.4 | 975.1 ± 27.6 | 549.6 ± 22.3 | 455.2 ± 9.6 | 491.6 ± 16.3 | 1327.3 ± 83.9 |
| **Kernai** | 658.2 ± 22.2 | 665.1 ± 32.0 | 304.5 ± 18.2 | 227.8 ± 9.2 | 274.2 ± 8.3 | 906.1 ± 43.6 |
| **Žadeikiai** | 73.0 ± 2.9 | 2136.8 ± 118.3 | 393.4 ± 24.3 | 460.7 ± 25.1 | 951.6 ± 55.3 | 1981.1 ± 38.7 |
| **Galvokai** | 209.4 ± 12.1 | 2325.3 ± 92.3 | 470.8 ± 15.3 | 515.0 ± 25.6 | 1002.9 ± 28.9 | 2097.3 ± 57.0 |
| **Giteniškė** | 525.2 ± 16.5 | 897.5 ± 15.5 | 326.4 ± 4.6 | 323.7 ± 23 | 370.6 ± 13.8 | 1279.1 ± 13.3 |
| **Šalčininkėliai** | 571.4 ± 1.3 | 1963.5 ± 36.6 | 513.8 ± 3.6 | 639.4 ± 14.8 | 1016.5 ± 14.4 | 2170.0 ± 49.3 |
| **Bitėnai** | 402.7 ± 2.1 | 237.3 ± 4.6 | 144.6 ± 1.6 | 120.8 ± 2.3 | 101.5 ± 1.0 | 442.3 ± 5.3 |
| **Pagramantis** | 746.5 ± 42.1 | 783.7 ± 42.8 | 389.6 ± 23.5 | 343.8 ± 18.3 | 320.8 ± 17.9 | 1087.2 ± 55.2 |
| **Kūprė** | 63.3 ± 2.9 | 1773.9 ± 75.8 | 284.2 ± 5.2 | 528.7 ± 24.5 | 731.7 ± 3.2 | 2201.2 ± 36.1 |
| **Bruknynė** | 734.3 ± 29.4 | 1973.8 ± 77.0 | 583.5 ± 19.0 | 663.9 ± 27.8 | 1022.9 ± 18.5 | 2234.1 ± 43.8 |
| **Viršilai** | 15.4 ± 0.1 | 692.4 ± 51.2 | 110.6 ± 3.6 | 168.9 ± 9.1 | 376.1 ± 4.3 | 713.5 ± 18.5 |
| **Labanoras (a)** | 1034.1 ± 1.5 | 998.7 ± 13.2 | 444.1 ± 3.0 | 539.4 ± 6.7 | 557.7 ± 5.6 | 1847.4 ± 15.7 |
| **Labanoras (b)** | 889.0 ± 17.0 | 1924.9 ± 34.4 | 530.3 ± 30.6 | 594.3 ± 32.5 | 878.3 ± 14.5 | 2316.6 ± 117.8 |
| **Komarinė** | 816.4 ± 43.6 | 947.4 ± 13.8 | 407.2 ± 11.4 | 385.7 ± 1.1 | 406.6 ± 30.0 | 1390.7 ± 20.1 |
| **Marcinkonys** | 1474 ± 94.6 | 1684.1 ± 22.8 | 668.7 ± 37.7 | 512.6 ± 12.5 | 893.4 ± 2.8 | 1966.3 ± 70.7 |
| **Šilainė** | 870.2 ± 44.7 | 1348.1 ± 36.9 | 409.6 ± 31.2 | 415.6 ± 16.8 | 619.0 ± 22.5 | 1671.7 ± 62.0 |
| **Smėlynė** | 2136.4 ± 59.7* | 3409.7 ± 108.4* | 1241.3 ± 33.1* | 853.8 ± 39.3* | 1345.3 ± 37.1* | 3473.4 ± 136.6* |
| **Ilgalaukiai** | 777.2 ± 14.0 | 717.9 ± 43.1 | 407.0 ± 7.8 | 319.6 ± 12.1 | 362.3 ± 15.3 | 1187.9 ± 42.0 |
| **Vosniūnai** | 1674.3 ± 89.7 | 2062.1 ± 118.5 | 816.4 ± 44.6 | 494.5 ± 28.2 | 954.0 ± 55.6 | 2087.1 ± 112.2 |
| **Kadagynai** | 1087.3 ± 82.4 | 958.0 ± 5.8 | 468.9 ± 37.5 | 397.6 ± 7.2 | 410.2 ± 34.0 | 1535.6 ± 42.5 |
| **Tolkūnai** | 2094.3 ± 59.4* | 1691.5 ± 93.5 | 1298.0 ± 46.7* | 760.7 ± 2.5 | 1049.6 ± 26.0 | 2739.1 ± 45.4 |
| **Bakūriškis** | 27.9 ± 2.2 | 2724.2 ± 48.5 | 420.7 ± 11.0 | 740.5 ± 29.7 | 1202.2 ± 27.4 | 2756.9 ± 74.3 |
| **Šilinė** | 1346.6 ± 78.8 | 1124.4 ± 80.9 | 502.6 ± 4.4 | 334.0 ± 13.3 | 484.8 ± 19.6 | 1378.3 ± 61.4 |
| **Tyrelis** | 627.7 ± 42.8 | 613.3 ± 21.6 | 327.1 ± 18.1 | 289.7 ± 9.3 | 263.8 ± 7.7 | 927.9 ± 34.7 |
| **Pažemys** | 830.4 ± 21.8 | 2336.4 ± 38.4 | 638.8 ± 14.8 | 649.0 ± 11.5 | 1045.9 ± 35.3 | 2726.7 ± 56.4 |

Results are presented as means ± SD of the samples collected at different locations at the end of September 2019. Values marked with * in the same column indicate the highest ($p < 0.05$) amounts in samples.

**Table S13.** Contents of triterpenoid acids and sterols (µg/g DW) ± SD in lingonberry leaves, collected at different locations.

| Location | Maslinic acid | Corosolic acid | Betulinic acid | Oleanolic acid | Ursolic acid | β-Sitosterol |
|---|---|---|---|---|---|---|
| **Apūniškis** | 26.8 ± 1.3 | 85.4 ± 2.4 | NQ | 157.4 ± 2.7 | 912.2 ± 20.4 | 428.8 ± 12.1* |
| **Plunksnuočiai** | 23.6 ± 0.8 | 58.3 ± 2.1 | NQ | 241.5 ± 0.7 | 947.0 ± 30.2 | 341.4 ± 12.3 |
| **Šakarva** | 15.6 ± 0.2 | 77.7 ± 2.4 | 16.5 ± 0.3 | 287.5 ± 3.7 | 1247.8 ± 28.2 | 347.9 ± 2.9 |
| **Andrioniškis** | 24.1 ± 1.5 | 79.8 ± 1.4 | 6.5 ± 0.6 | 215.3 ± 2.7 | 1132.1 ± 62.7 | 376.6 ± 9.6 |
| **Jurgionys** | 12.1 ± 0.5 | 56.1 ± 0.6 | 20.3 ± 0.7 | 191.5 ± 4.0 | 957.4 ± 15.2 | 440.8 ± 3.9* |
| **Kernai** | 12.0 ± 0.5 | 74.7 ± 2.9 | NQ | 235.3 ± 2.9 | 1197.6 ± 33.9 | 410.5 ± 0.3* |
| **Žadeikiai** | 26.4 ± 0.8 | 91.1 ± 2.7 | ND | 277.7 ± 3.7 | 1220.8 ± 22.6 | 375.8 ± 9.4 |
| **Galvokai** | 13.8 ± 0.2 | 67.1 ± 0.2 | ND | 249.9 ± 5.4 | 1275.9 ± 33.0 | 393.8 ± 18.0 |
| **Giteniškė** | 23.1 ± 1.3 | 47.8 ± 2.0 | 12.7 ± 0.1 | 168.0 ± 1.9 | 805.8 ± 10.7 | 321.5 ± 11.2 |
| **Šalčininkėliai** | 33.6 ± 1.6 | 71.4 ± 3.5 | NQ | 229.3 ± 4.6 | 1158.0 ± 10.6 | 395.4 ± 11.6 |
| **Bitėnai** | 9.1 ± 0.3 | 75.2 ± 2.8 | NQ | 219.9 ± 6.2 | 1118.9 ± 8.6 | 285.8 ± 2.6 |
| **Pagramantis** | 30.6 ± 1.1 | 84.6 ± 4.9 | ND | 309.8 ± 14.5 | 1432.4 ± 72.4 | 346.8 ± 2.6 |
| **Kūprė** | 29.4 ± 0.8 | 47.4 ± 1.8 | 17.0 ± 0.2 | 130.3 ± 2.2 | 580.3 ± 18.4 | 441.1 ± 2.5* |
| **Bruknynė** | 34.7 ± 0.2 | 74.3 ± 4.0 | ND | 228.4 ± 3.7 | 1150.1 ± 24.1 | 410.1 ± 16.7* |
| **Viršilai** | 7.1 ± 0.1 | 38.2 ± 1.9 | NQ | 212.8 ± 0.2 | 1078.8 ± 17.9 | 326.4 ± 12.2 |
| **Labanoras (a)** | 62.8 ± 1.7* | 205.3 ± 4.7* | 84.3 ± 3.2* | 1333.6 ± 48.9* | 1626.3 ± 55.8* | 438.7 ± 13.0* |
| **Labanoras (b)** | 16.1 ± 0.1 | 50.2 ± 1.2 | 23.9 ± 1.9 | 364.3 ± 12.7 | 755.6 ± 21.5 | 373.0 ± 13.5 |
| **Komarinė** | 22.7 ± 1.0 | 81.1 ± 3.5 | ND | 309.5 ± 9.7 | 698.2 ± 35.6 | 330.5 ± 3.9 |
| **Marcinkonys** | 33.7 ± 1.6 | 96.5 ± 2.5 | 16.3 ± 1.1 | 213.0 ± 2.5 | 867.8 ± 11.9 | 319.3 ± 15.7 |
| **Šilainė** | 8.4 ± 0.1 | 51.3 ± 1.2 | 27.3 ± 0.7 | 158.1 ± 5.3 | 704.3 ± 36.9 | 314.7 ± 9.1 |
| **Smėlynė** | 10.2 ± 0.6 | 50.4 ± 0.5 | 4.1 ± 0.5 | 201.2 ± 12.0 | 940.6 ± 4.1 | 391.3 ± 4.0 |
| **Ilgalaukiai** | 12.6 ± 0.4 | 61.4 ± 1.0 | 3.6 ± 0.2 | 247.8 ± 12.8 | 1201.8 ± 62.7 | 302.0 ± 7.0 |
| **Vosniūnai** | 7.1 ± 0.4 | 54.7 ± 3.3 | 40.7 ± 3.1 | 182.5 ± 2.5 | 814.8 ± 4.5 | 311.8 ± 16.2 |
| **Kadagynai** | 11.4 ± 0.4 | 66.4 ± 3.5 | 36.3 ± 0.4 | 208.7 ± 6.9 | 789.2 ± 5.7 | 348.1 ± 19.5 |
| **Tolkūnai** | 32.7 ± 0.9 | 172.0 ± 6.8 | 56.7 ± 1.6 | 269.4 ± 12.4 | 1265.0 ± 62.3 | 408.7 ± 6.0* |
| **Bakūriškis** | 8.6 ± 0.9 | 117.2 ± 5.1 | 30.7 ± 1.3 | 193.9 ± 2.6 | 904.5 ± 32.8 | 396.1 ± 3.1 |
| **Šilinė** | 44.4 ± 1.4 | 193.1 ± 1.2* | 28.3 ± 1.4 | 237.3 ± 3.1 | 1118.0 ± 46.9 | 359.2 ± 17.1 |
| **Tyrelis** | 16.8 ± 0.5 | 89.7 ± 2.8 | ND | 263.5 ± 14.5 | 1265.7 ± 15.6 | 346.7 ± 10.8 |
| **Pažemys** | 17.2 ± 0.1 | 91.2 ± 0.5 | 20.8 ± 1.4 | 361.4 ± 16.8 | 832.9 ± 13.2 | 379.5 ± 13.9 |

Results are presented as means ± SD of the samples collected at different locations at the end of September 2019. Values marked with * in the same column indicate the highest ($p < 0.05$) amounts in samples. ND—not detected, NQ—not quantified (amount below LOQ).

**Table S14.** Contents of neutral triterpenoids (μg/g DW ± SD) in lingonberry leaves, collected at different locations.

| Location | Betulin | Erythrodiol | Uvaol | Lupeol | α-Amyrin | β-Amyrin | Friedelin |
|---|---|---|---|---|---|---|---|
| **Apūniškis** | 128.7 ± 4.0 | 30.8 ± 1.2 | 136.2 ± 3.5 | 157.3 ± 3.0 | 2145.4 ± 14.5 | 148.5 ± 5.4 | 172.7 ± 7.0 |
| **Plunksnuočiai** | 148.8 ± 6.7 | 52.8 ± 1.0 | 102.6 ± 2.0 | 445.7 ± 5.8 | 677.3 ± 14.9 | 1349.9 ± 67.0* | 80.5 ± 3.3 |
| **Šakarva** | 120.4 ± 0.5 | 41.6 ± 1.3 | 165.7 ± 1.2 | 852.5 ± 25.7 | 742.8 ± 15.7 | 1058.4 ± 18.0 | 54.0 ± 2.2 |
| **Andrioniškis** | 186.3 ± 0.6 | 39.0 ± 1.8 | 190 ± 6.8 | 398.9 ± 1.2 | 1462.1 ± 24.1 | 131.9 ± 5.4 | 505.8 ± 13.1 |
| **Jurgionys** | 227.0 ± 5.5 | 57.3 ± 1.5 | 183.2 ± 1.3 | 1142.3 ± 6.6 | 1034.7 ± 23.7 | 101.2 ± 0.7 | 251.1 ± 11.8 |
| **Kernai** | 293.4 ± 5.3 | 31.0 ± 0.3 | 123.5 ± 7.0 | 526.8 ± 16.0 | 963.7 ± 4.1 | 224.4 ± 11.0 | 2020.2 ± 19.5* |
| **Žadeikiai** | 315.7 ± 12.3* | 101.7 ± 5.4 | 328.6 ± 6.9 | 173.4 ± 8.4 | 2303.3 ± 75.1 | 155.5 ± 5.7 | 234.5 ± 12.0 |
| **Galvokai** | 275.8 ± 9.5 | 92.2 ± 1.9 | 129.6 ± 3.8 | 210.8 ± 2.0 | 3266.1 ± 123.5* | 224.3 ± 3.0 | 217.1 ± 4.5 |
| **Giteniškė** | 182.4 ± 1.7 | 35.9 ± 0.7 | 51.9 ± 1.7 | 1489.4 ± 23.8 | 819.6 ± 30.6 | 120.3 ± 0.1 | 127.9 ± 5.4 |
| **Šalčininkėliai** | 280.2 ± 0.4 | 45.1 ± 2.2 | 150.2 ± 2.5 | 346.1 ± 18.6 | 1285.9 ± 7.6 | 160.5 ± 4.3 | 620.7 ± 4.6 |
| **Bitėnai** | 123.9 ± 5.5 | 26.2 ± 1.5 | 81.7 ± 3.8 | 696.1 ± 27.3 | 1018.2 ± 22.1 | 197.7 ± 1.6 | 1255.0 ± 17.0 |
| **Pagramantis** | 212.1 ± 7.6 | 52.7 ± 1.5 | 124.4 ± 6.8 | 152.8 ± 6.5 | 1698.6 ± 54.7 | 267.9 ± 7.4 | 195.5 ± 8.4 |
| **Kūprė** | 248.9 ± 0.4 | 96.2 ± 5.1 | 67.7 ± 0.6 | 144.8 ± 8.3 | 857.6 ± 16.3 | 93.7 ± 3.1 | 183.1 ± 4.0 |
| **Bruknynė** | 298.5 ± 3.5 | 50.0 ± 1.8 | 149.4 ± 8.4 | 569.5 ± 3.9 | 1294.7 ± 9.6 | 135.0 ± 3.0 | 457.8 ± 10.7 |
| **Viršilai** | 128.4 ± 5.6 | 24.4 ± 1.8 | 81.2 ± 1.2 | 220.3 ± 10.9 | 1663.1 ± 53.2 | 244.1 ± 10.8 | 115.8 ± 4.0 |
| **Labanoras (a)** | 218.4 ± 1.8 | 306.4 ± 7.8* | 437.8 ± 9.7* | 1103.0 ± 55.7 | 3249.3 ± 41.3* | 1175.5 ± 7.0 | 244.7 ± 8.3 |
| **Labanoras (b)** | 107.0 ± 3.9 | 64.1 ± 2.0 | 221.8 ± 0.2 | 869.5 ± 22.1 | 1287.7 ± 28.9 | 492.6 ± 23.3 | 324.2 ± 15.8 |
| **Komarinė** | 154.7 ± 3.0 | 67.9 ± 3.9 | 100.5 ± 4.2 | 318.8 ± 7.8 | 1268.0 ± 14.7 | 957.4 ± 38.7 | 186.4 ± 7.0 |
| **Marcinkonys** | 137.1 ± 0.7 | 31.1 ± 2.0 | 145.9 ± 4.6 | 591.7 ± 19.4 | 1743.7 ± 46.2 | 427.5 ± 4.0 | 288.1 ± 12.2 |
| **Šilainė** | 237.1 ± 4.2 | 17.5 ± 0.4 | 72.8 ± 3.7 | 1933.5 ± 19.1* | 1123.8 ± 39.8 | 155.4 ± 1.0 | 82.7 ± 3.7 |
| **Smėlynė** | 171.7 ± 6.6 | 13.3 ± 1.1 | 95.0 ± 3.3 | 851.5 ± 14.4 | 2024.7 ± 37.8 | 148.1 ± 5.9 | 190.5 ± 4.3 |
| **Ilgalaukiai** | 170.1 ± 5.4 | 16.4 ± 0.2 | 100.8 ± 3.3 | 527.5 ± 7.5 | 1055.9 ± 44.7 | 139.1 ± 5.2 | 686.9 ± 23.0 |
| **Vosniūnai** | 110.8 ± 2.2 | 29.4 ± 1.0 | 127.1 ± 0.8 | 183.2 ± 4.1 | 1715.4 ± 28.8 | 121.3 ± 4.1 | 196.9 ± 8.9 |
| **Kadagynai** | 162.3 ± 6.6 | 20.3 ± 0.6 | 47.9 ± 1.8 | 592.1 ± 10.6 | 1147.5 ± 57.5 | 339.8 ± 1.9 | 148.1 ± 0.8 |
| **Tolkūnai** | 146.5 ± 3.0 | 38.3 ± 1.1 | 152.2 ± 2.6 | 141.1 ± 6.2 | 2556.0 ± 57.8 | 112.9 ± 6.4 | 166.1 ± 5.8 |
| **Bakūriškis** | 223.8 ± 8.3 | 16.4 ± 1.1 | 69.9 ± 1.8 | 239.0 ± 9.5 | 2084.8 ± 76.1 | 116.3 ± 2.7 | 268.1 ± 3.9 |
| **Šilinė** | 276.7 ± 9.4 | 25.5 ± 1.6 | 62.0 ± 3.5 | 1232.6 ± 12.3 | 1104.8 ± 23.8 | 113.1 ± 4.6 | 1891.8 ± 49.4 |
| **Tyrelis** | 216.6 ± 2.6 | 22.9 ± 0.9 | 63.3 ± 1.7 | 189.7 ± 3.1 | 1699.2 ± 45.6 | 267.4 ± 6.1 | 272.7 ± 13.2 |
| **Pažemys** | 325.0 ± 16.4* | 64.7 ± 2.7 | 69.5 ± 0.3 | 645.6 ± 1.3 | 978.9 ± 44.5 | 994.7 ± 14.1 | 368.1 ± 1.8 |

Results are presented as means ± SD of the samples collected at different locations at the end of September 2019. Values marked with * in the same column indicate the highest ($p < 0.05$) amounts in samples.

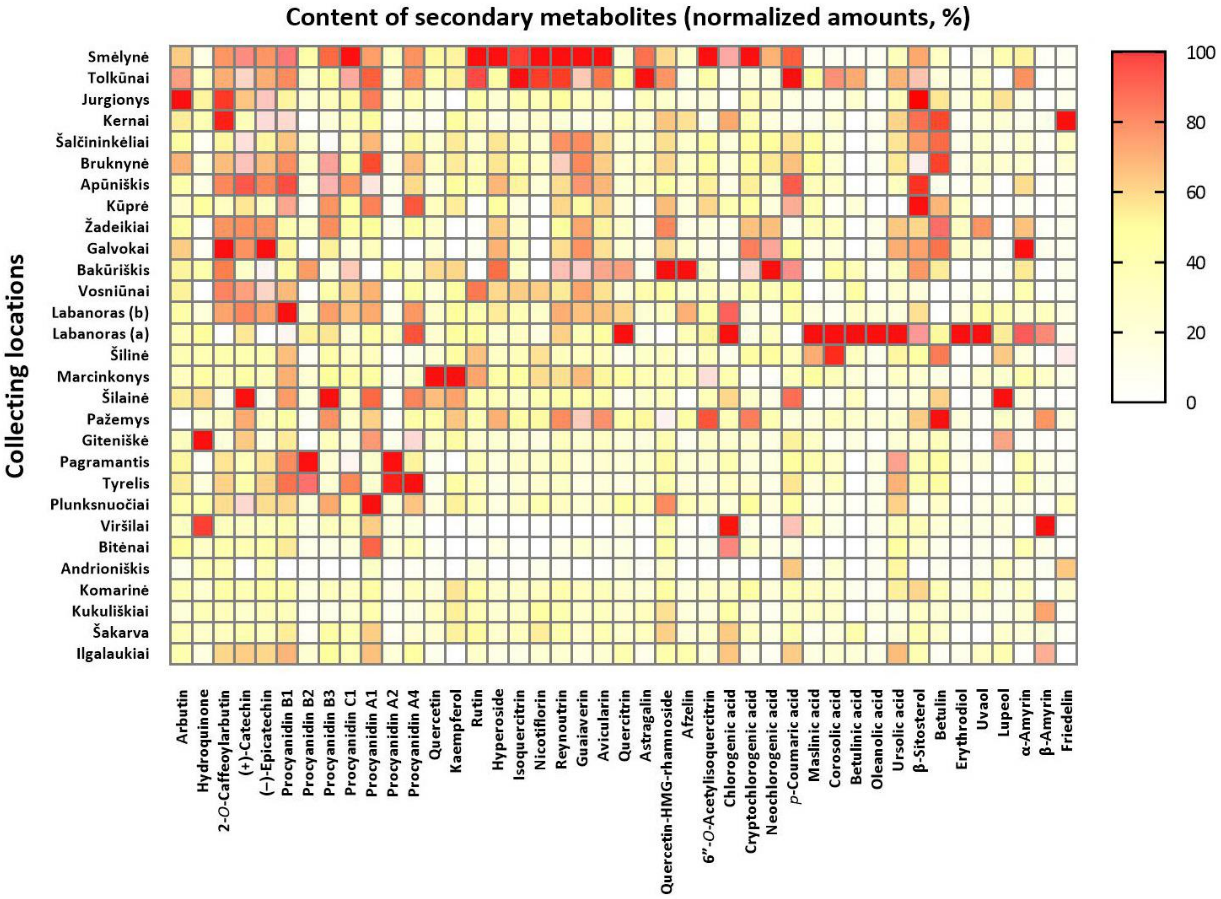


**Figure S2.** Heatmap estimating contents of phenolic and triterpenic compounds in lingonberry leaves based on their collecting locations.

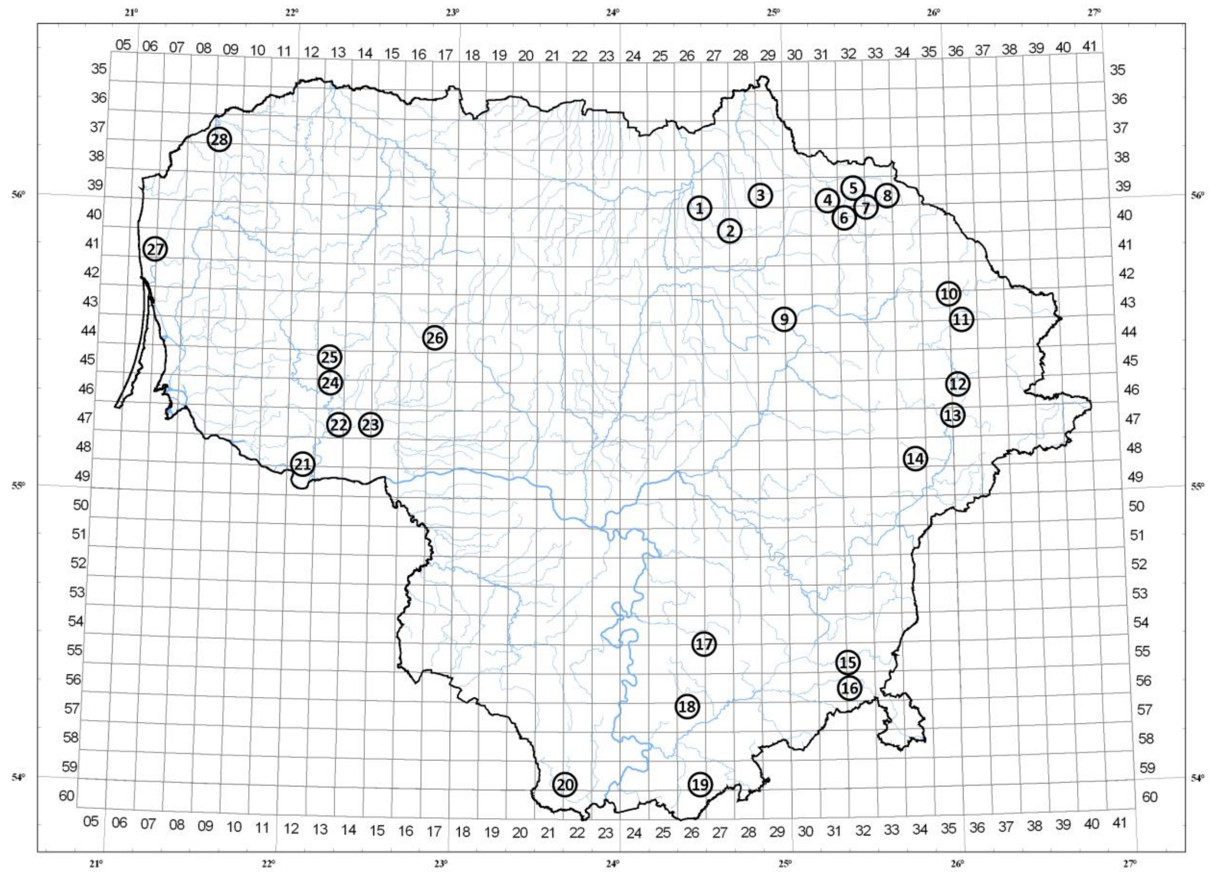


**Figure S3.** Collecting locations of lingonberry leaves samples in Lithuania: (1) Žadeikiai, (2) Vosniūnai, (3) Galvokai, (4) Viršilai, (5) Plunksnuočiai, (6) Ilgalaukiai, (7) Apūniškis, (8) Bakūriškis, (9) Andrioniškis, (10) Pažemys, (11) Giteniškė, (12) Smėlynė, (13) Šakarva, (14) Labanoras (a, b), (15) Šalčininkėliai, (16) Bruknynė, (17) Jurgionys, (18) Tolkūnai, (19) Marcinkonys, (20) Šilainė, (21) Bitėnai, (22) Šilinė, (23) Komarinė, (24) Pagramantis, (25) Tyrelis, (26) Kūprė, (27) Kukuliškiai, (28) Kernai.

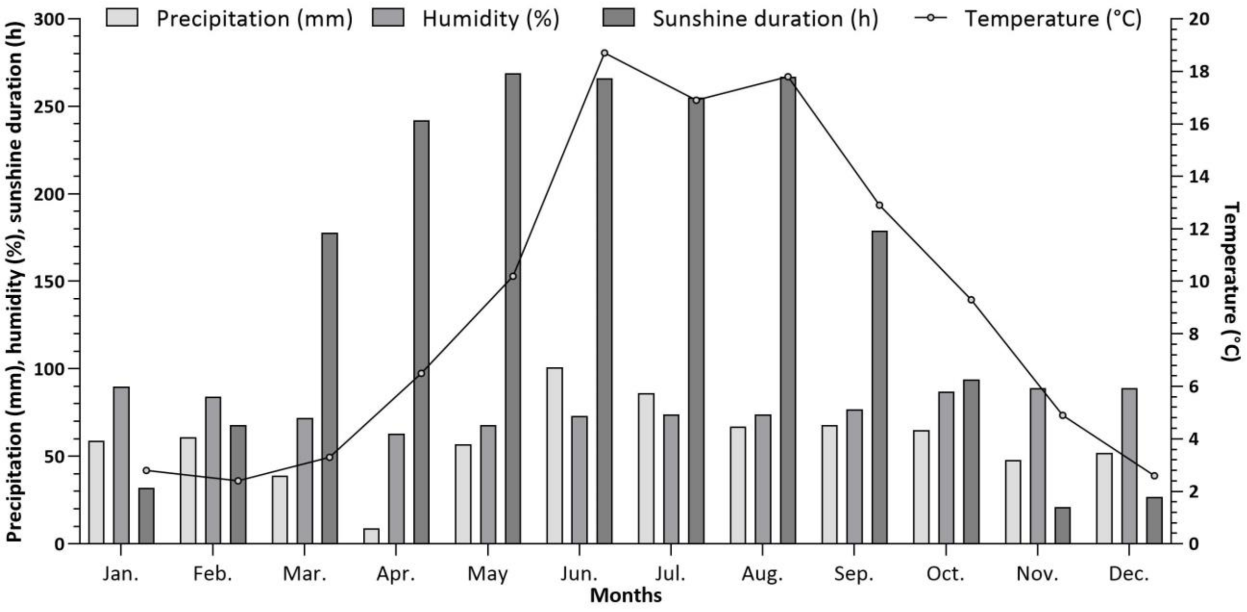


**Figure S4.** Dynamics of meteorological factors during one year testing period in Lithuania.
